# Ultrasound-driven electronically programmable microbubbles

**DOI:** 10.1126/sciadv.aeg5408

**Published:** 2026-10-14

**Authors:** Zhiyuan Zhang, Jinsong Zhang, Alyosha C. Molnar, Itai Cohen

**Affiliations:** ^1^ Laboratory of Atomic and Solid State Physics, Cornell University, Ithaca, NY 14850, USA.; ^2^ Kavli Institute at Cornell for Nanoscale Science, Cornell University, Ithaca, NY 14850, USA.; ^3^ School of Electrical and Computer Engineering, Cornell University, Ithaca, NY 14850, USA.

## Abstract

Ultrasound-actuated microbubbles have served as efficient microscale engines demonstrating wireless actuation, simplified robotic architectures, and rapid response. Despite recent advances, a limitation of many microbubble technologies is that they use passive capturing of bubbles. The lack of control over bubble placement and size hinders the ability to individually address specified bubbles, a feature that would be important for performing multiple functions. Here, we present an on-chip bubble customization platform that enables active control of individual microbubbles. Instead of trapping bubbles, we purposely generate them via electrolysis on electrode arrays. The bubbles, when actuated by an external ultrasound input, create a variety of programmed flows ranging from streaming due to volumetric oscillation of a single bubble to diverse symmetry-broken directional flows when multiple bubbles interact. The bubble configuration can also be altered by simply increasing the electrolysis voltage on specified electrodes to detach the unneeded bubbles. We find that in this regime, when coupled to acoustic oscillations, we can generate a distinct bubble shooting flow pattern whose flow direction is selectively controlled by the incident ultrasound frequency. Collectively, the active bubble reconfiguration ability unlocks a range of previously inaccessible acoustofluidic functions in a single device with unprecedented controllability.

## INTRODUCTION

Ultrasound resonant microbubbles have emerged as a powerful driver for numerous technologies. At the microscale, microbubbles have been used to achieve propulsion, steering, and deformation of actuators (*1*–*3*), swimmers (*4*–*12*), and end-effectors (*13*–*15*). Scaling up, microbubble arrays are used to enhance acoustic radiation forces (*16*–*18*), enable high-throughput microengineering (*19*–*23*), and drive deformation of soft artificial muscles (*24*).

While current ultrasound-microbubble systems have demonstrated advanced functionality, scalability, and rapid response, their lifetime, programmability, and controllability remain limited. For example, under continuous oscillation, microbubbles are prone to rectified diffusion, which leads to uncontrolled growth, resonance drift, and temporal instability (*25*, *26*). As a result, escaping or collapsing microbubbles make acoustic actuation unstable, which, in the long run, leads to device failure. An additional issue hampering systems that use multiple bubbles is that unwanted excitation coupling among bubbles compromises frequency selectivity and independent control (*10*, *11*, *27*). More critically, such couplings make it nearly impossible to selectively address individual bubbles if they are part of an array of similarly sized bubbles (*15*, *16*, *24*, *28*). Many of these limitations arise because microbubbles used in most acoustofluidic systems are passively captured, which does not allow for altering their configurations and rejuvenating devices. One way forward is to change the passive capture paradigm and introduce active, real-time, and in situ microbubble generation and modulation in next-generation ultrasound-microbubble systems.

Bubbles can be artificially generated by many methods, including light stimuli (*29*–*32*), chemical response (*33*, *34*), physical cavitation (*35*, *36*), syringe pumps (*37*, *38*), and boiling (*39*, *40*). However, these methods are often low-throughput, cannot precisely control the bubble generation rate, and cannot dynamically modulate bubble states. Another bubble generation method is electrolysis, which has been widely used across scales for microrobotic systems (*41*–*46*), hydrogen production, electroflotation, heat transfer studies, and wastewater treatment (*47*–*53*). Here, we unite electrolysis-driven bubble generation with ultrasound actuation to create an active microbubble customization platform. We show that the platform is capable of real-time bubble generation and modulation and provides multi–degree-of-freedom flow programming. At its core, it transforms microbubbles from passive elements in a device architecture into active reconfigurable agents, pioneering a previously unidentified paradigm for ultrasound-driven technologies.

## RESULTS

### Design and characterization

In our approach, a single electrode switches the microbubble states between bubble generation and bubble detachment by tuning the voltage of the localized electrolysis (as schematized in Fig. 1A). In an ionic aqueous environment, a hydrogen microbubble will be generated on the electrode via reduction, $2H_{2}O+2e^{-}\to H_{2}↑+2{\text{OH}}^{-}$, when a low-voltage direct current (dc) bias renders the microelectrode cathodic with respect to a remote counter electrode (Fig. 1A, a). At low voltages, electrolysis will lead to a low gas-generation rate and a slow increase in bubble volume over time. Upon turning off the electrolysis, the bubble will stop growing and be trapped on the electrode surface due to the surface tension and pinning force. Once the bubble reaches the desired size, externally incident ultrasound waves will excite oscillations that induce flows (Fig. 1A, b). To clear a bubble, we would simply increase the electrode voltage to generate sufficient hydrogen to detach the bubble (Fig. 1A, c).

**Fig. 1.**
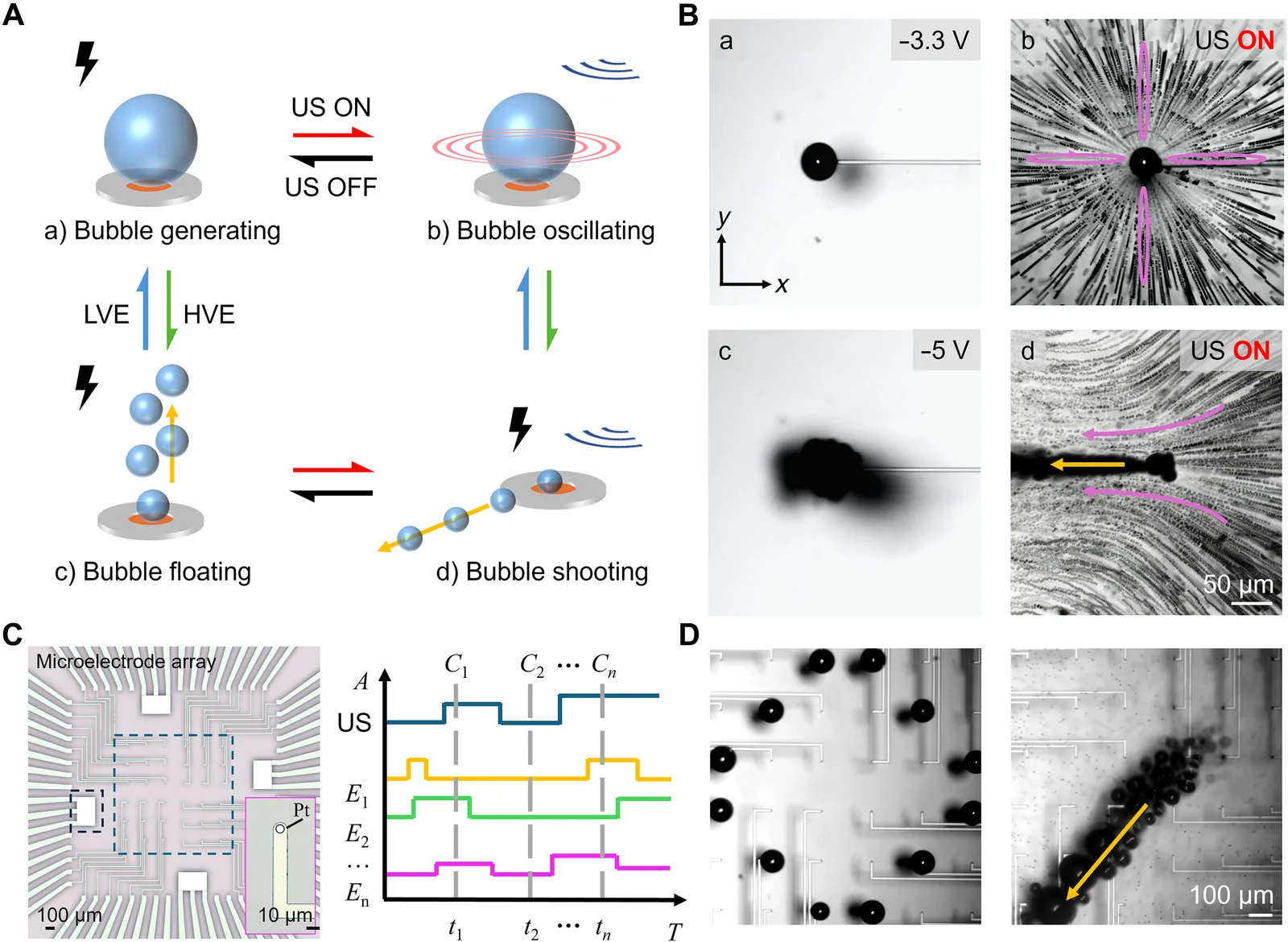
Ultrasound-driven electronically programmable microbubbles. (**A**) Schematic illustration of bubble state switching on a single electrode, including (a) bubble generating, (b) bubble oscillating, (c) bubble floating, and (d) bubble shooting. HVE and LVE denote high-voltage and low-voltage electrolysis, respectively. US denotes ultrasound. (**B**) Representative images corresponding to the four bubble states in (A): (a) generation of a single hydrogen bubble via −3.3 V electrolysis; (b) bubble oscillation under 74 kHz and 10 V_pp_ ultrasound; (c) bubbling induced by −5 V electrolysis; and (d) bubble shooting under −5 V electrolysis combined with 74 kHz and 20 V_pp_ ultrasound. Flow fields were visualized using 5-μm-diameter tracer particles and superimposed frames, with magenta arrows indicating flow directions. Yellow arrows indicate bubble moving directions. (**C**) Left: Photograph of the platform featuring a 6 × 6 microelectrode array. Blue and black dashed boxes indicate the cathode array and counter electrode, respectively. The inset shows a single Pt electrode. Right: Schematic of the time-staggered application of dc voltage pulses to the electrodes and ultrasound excitation to the transducer. *E_i_*, *C_i_*, and *t_i_* represent the electrodes, bubble configurations, and time points, respectively. (**D**) Representative demonstrations of distinct bubble configurations and associated flow functionalities.

We found that this approach worked remarkably well in the experiments. In our apparatus, the electrode was fabricated using standard lithographic techniques. Briefly, we fabricated a platinum (Pt) layer that was covered by an insulating SiO_2_ layer. A small circular region of the Pt was left exposed and acted as the electrode. Through systematic optimization of the electrode dimensions, the best performance was obtained for a 6-μm electrode diameter and 2-μm insulation height, and these dimensions were used for subsequent experiments (fig. S1). To generate acoustic fields, we bonded a cylindrical polydimethylsiloxane (PDMS) acoustic chamber surrounding the region of interest and a circular piezoelectric transducer (PZT) near the acoustic chamber on the same substrate. 1× Phosphate-buffered saline (PBS) served as the electrolyte solution (fig. S2). When we applied a dc bias of −3.3 V, a single microbubble nucleated and grew (Fig. 1B, a, and movie S1). The volume of the generated bubble is linearly related to the electrolysis time, V∝t, consistent with Faraday’s law of electrolysis (*54*). Meanwhile, the resonant frequency of the bubble is inversely proportional to its radius f∝1/R, consistent with the inverse scaling Minnaert relationship (*55*) (fig. S1). Under 74-kHz ultrasound resonance, a 38-μm-diameter bubble resonantly oscillates and, in turn, induces strong localized volume streaming in the surrounding liquid as indicated by tracer particles (Fig. 1B, b). Details of the microfabrication process, experimental setup, and bubble characterization are provided in Materials and Methods. Control experiments showed that, during bubble generation without ultrasound excitation, no pronounced streaming was observed around the bubble (see initial stages in movie S1). We further simulated the temperature distribution throughout the acoustic chamber and in the vicinity of the generated bubble, both without and with ultrasound excitation (fig. S3). Simulations showed that, under the experimental operating conditions, the temperature field remained spatially uniform and close to the ambient temperature of 20°C. These results indicate that the observed streaming is not attributable to electrolysis-driven flow or local heating. We also found that when the driving field does not induce oscillations in the bubble, i.e., when the bubble is static, the acoustic field on its own is not sufficient to drive streaming of the tracer particles (fig. S4).

To eliminate the bubble, we applied a dc bias of −5 V. The electrode exhibited continuous bubbling. Bubbles then floated to the fluid surface due to buoyant forces in the *z* direction (Fig. 1B, c). The bubble size and generation rate versus the electrolysis voltage in this regime are shown in fig. S5. If the dc bias was removed, the bubbling stopped, and the electrode became bubble-free. If the bubbling was maintained and ultrasound was applied at 74 kHz, we observed bubbles being actively ejected and transported in the *x-y* plane, which we termed “bubble shooting” (Fig. 1A, d). This shooting also generated a long-distance shear flow in the liquid (Fig. 1B, d).

This microbubble reprogramming approach embodies a universal framework that can be adapted to multiple electrodes in arrays. Toward this end, we fabricated a 6 × 6 electrode array where each electrode was individually wired and controlled (left panel of Fig. 1C). Because the bubble state on each electrode could be individually tuned, the generated bubbles could be used alone or in combination by controlling a series of time-staggered dc voltage pulses for each electrode along with the globally applied ultrasound amplitude and frequency (right schematic of Fig. 1C). As such, the device allows for the customization of diverse bubble configurations, which in turn allows us to access various acoustofluidic topologies within a single device (Fig. 1D and movie S2). To highlight the platform’s potential capabilities, we demonstrated four acoustofluidic operations, including modulating bubble size, inducing bubble interactions, programming modular bubbles, and generating shooting bubbles.

### Bubble-size modulation and flow scaling

Controlling the oscillation intensity of microbubbles is complex due to their strongly nonlinear dynamic characteristics. Even minute differences in the ultrasound frequency and drive amplitude could lead to drastically different behaviors, challenging modeling and experiments (*56*–*58*). To achieve stable oscillation-intensity modulation, we used our platform to dynamically adjust the bubble size in real time as an independent control knob. As a proof of concept, we first generated a single hydrogen microbubble by applying a dc bias (−3.3 V for 1 s) at the center electrode of a 3 × 3 array (pitch 300 μm) and subsequently excited it with ultrasound at 62 kHz and 10 V_pp_ (peak-to-peak excitation voltage). When the bubble was small with a diameter of 41 μm, the oscillation was relatively weak, and the streaming was confined near its interface, which is consistent with off-resonant forced oscillation (Fig. 2A, a, and movie S3). We then increased the bubble size in a stepwise manner using additional 1-s electrolysis pulses. When the equilibrium diameters reached 54 μm (after 2 s of electrolysis) and then 62 μm (after 3 s of electrolysis), the bubble coupled more strongly to the imposed acoustic field, and the oscillation amplitude rose, which intensified the time-averaged streaming (Fig. 2A, b and c). Consistent with this picture, we found that the effective spatial extent of the streaming field increases approximately linearly with the bubble diameter (Fig. 2B), and the characteristic flow speed within the streaming region scales approximately with the square of the bubble diameter (Fig. 2C), reaching a maximum for the 69-μm bubble after 4 s of electrolysis (Fig. 2A, d).

**Fig. 2.**
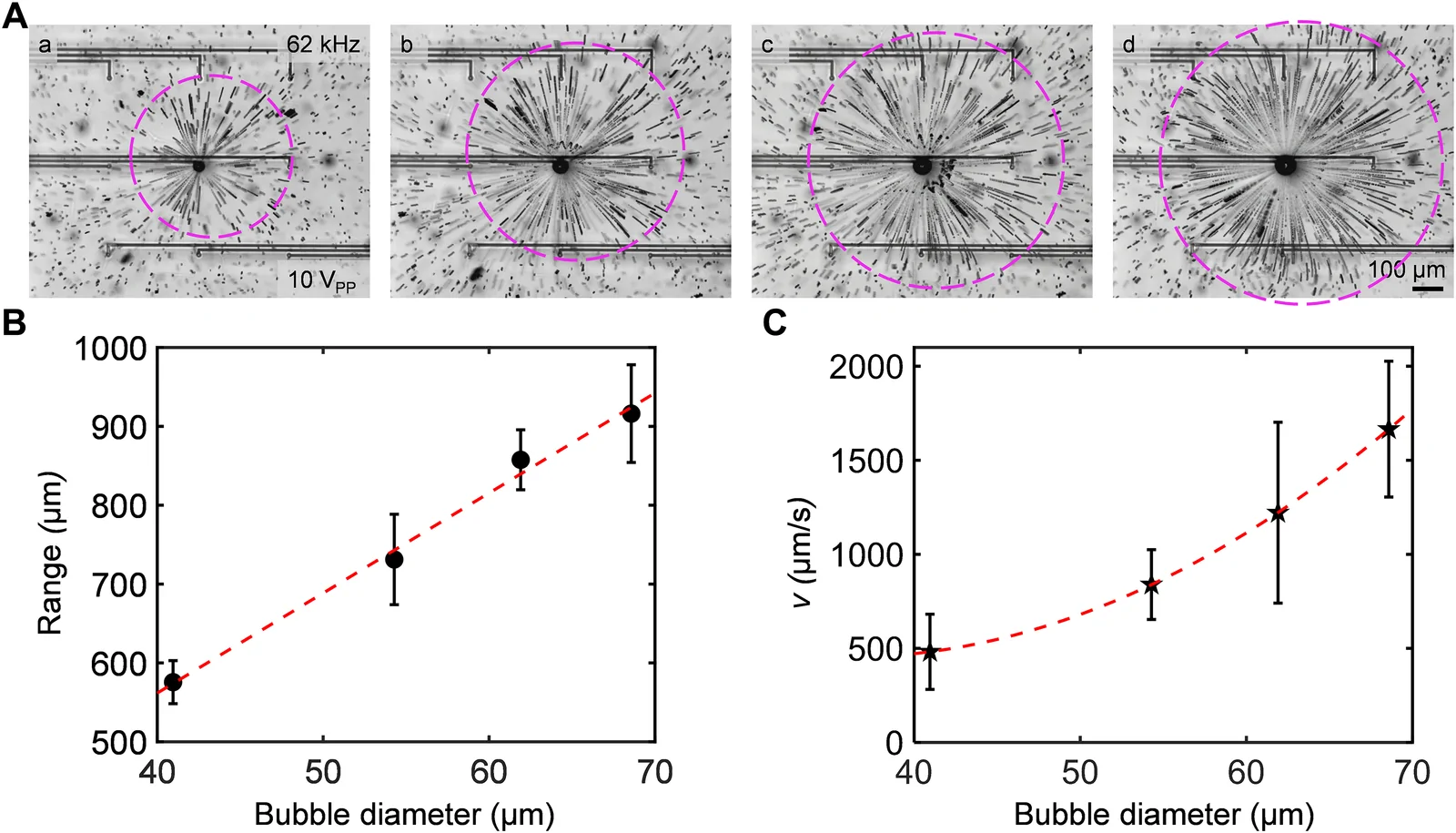
Bubble-size modulation and flow scaling. (**A**) Stepwise size modulation of a single microbubble, with diameters of (a) 41, (b) 54, (c) 62, and (d) 69 μm, achieved using additional 1-s electrolysis pulses. Under 62 kHz and 10 V_pp_ ultrasound, increasing bubble size led to intensified oscillations and enhanced acoustic streaming. Flow fields were visualized using 5-μm-diameter tracer particles and superimposed frames. The streaming ranges were determined from superimposed images of tracer-particle motion over 30 consecutive frames recorded during ultrasound excitation. The magenta dashed circles mark the outer boundary of the coherent tracer-trajectory envelope, where most tracer trajectories showed displacements greater than 30 μm and remained distinguishable from background Brownian motion and random drift. The same criterion was applied to all measurements. (**B**) The streaming range diameter as a function of bubble diameter. The dashed line indicates a linear fit. Error bars represent mean ± SD (*n* = 4 independent measurements). (**C**) Average streaming speed as a function of bubble diameter. The average streaming speed was determined as the mean tracer-particle speed within the magenta dashed circle. The dashed line indicates a quadratic fit. Error bars represent mean ± SD (*n* = 8 independent measurements).

Further growth, however, qualitatively altered the oscillation symmetry (fig. S6). The bubble response became unstable, intermittently switching among oscillation modes, undergoing oscillation suppression, and occasionally detaching from the electrode, due to translational and/or shape deformation components (*59*–*61*). In traditional passive capturing, time-dependent size changes often induce abrupt, history-dependent transitions that compromise reproducibility. With our platform, however, if the bubble is too big to begin with or becomes uncontrollably larger during excitation, we can easily clear it and generate a new bubble. Thus, electrochemical control of bubble size offers a new degree of freedom to linearly reconfigure the oscillation behavior of a bubble and the topology of the correlated flow field.

### Controlled bubble-pair interaction and flow polarization

In most acoustofluidic settings, the interactions between bubbles are experimentally difficult to systematically investigate because the bubble generation site, timing, and size ratio are not actively controlled (*37*, *62*, *63*). To address this limitation, we used our platform to enable deterministic, addressable, and spatiotemporally programmed bubble generation. Ultrasound-driven bubble pairs offer a sensitive objective for bubble interaction, where mutual scattering and secondary Bjerknes forces can substantially alter bubble behavior and reshape the surrounding acoustic streaming field (*64*–*67*). In our experiments with a 3 × 3 electrode array (pitch 50 μm), a primary bubble (55 μm in diameter) first formed and exhibited volumetric oscillation under 90 kHz and 7 V_pp_ (Fig. 3A, a, and movie S4). A secondary bubble (39 μm) was then generated at a left neighboring electrode. As the secondary bubble grew, we observed a change in the dynamic flow topology. When the second bubble was smaller than the first bubble, the streaming field became increasingly polarized in the direction toward the left, reflecting the growing contribution of the small bubble’s oscillations to global time-averaged streaming (Fig. 3A, b). When the two bubbles reached comparable sizes, the flow reorganized into four symmetric vortices, consistent with a near-symmetric superposition of two coupled oscillators (Fig. 3A, c). Further growth of the secondary bubble (59 μm) inverted the polarization to the right (Fig. 3A, d). To elucidate this flow behavior, we established the relationship between bubble size and oscillation phase based on the damped harmonic oscillator model and conducted parametric sweep simulations by varying the bubble-size ratio, oscillation-amplitude ratio, and phase difference of the bubble pair to identify the dominant factors. Briefly, we found that the flow polarization arises from a peristaltic mechanism induced by the phase difference between the oscillating bubbles. When one bubble leads the other during pulsation, constructive superposition of the flow field occurs on one side of the bubble pair while partial destructive interference occurs on the other, and vice versa. The relative phases of the bubbles are determined by both the driving acoustic field and the flows induced by the neighboring bubble. The observed flows are consistent with a phase difference between the bubbles, Δφ = 90° − 270°. The flow reversal observed, as one bubble continuously grows, can be attributed to changes in the relative oscillation phase and the swapping of the phase-leading and phase-lagging relationship between the two bubbles (see note S1 for detailed theoretical understanding and simulations). The generality of this behavior was further demonstrated using different bubble pairs (fig. S7).

**Fig. 3.**
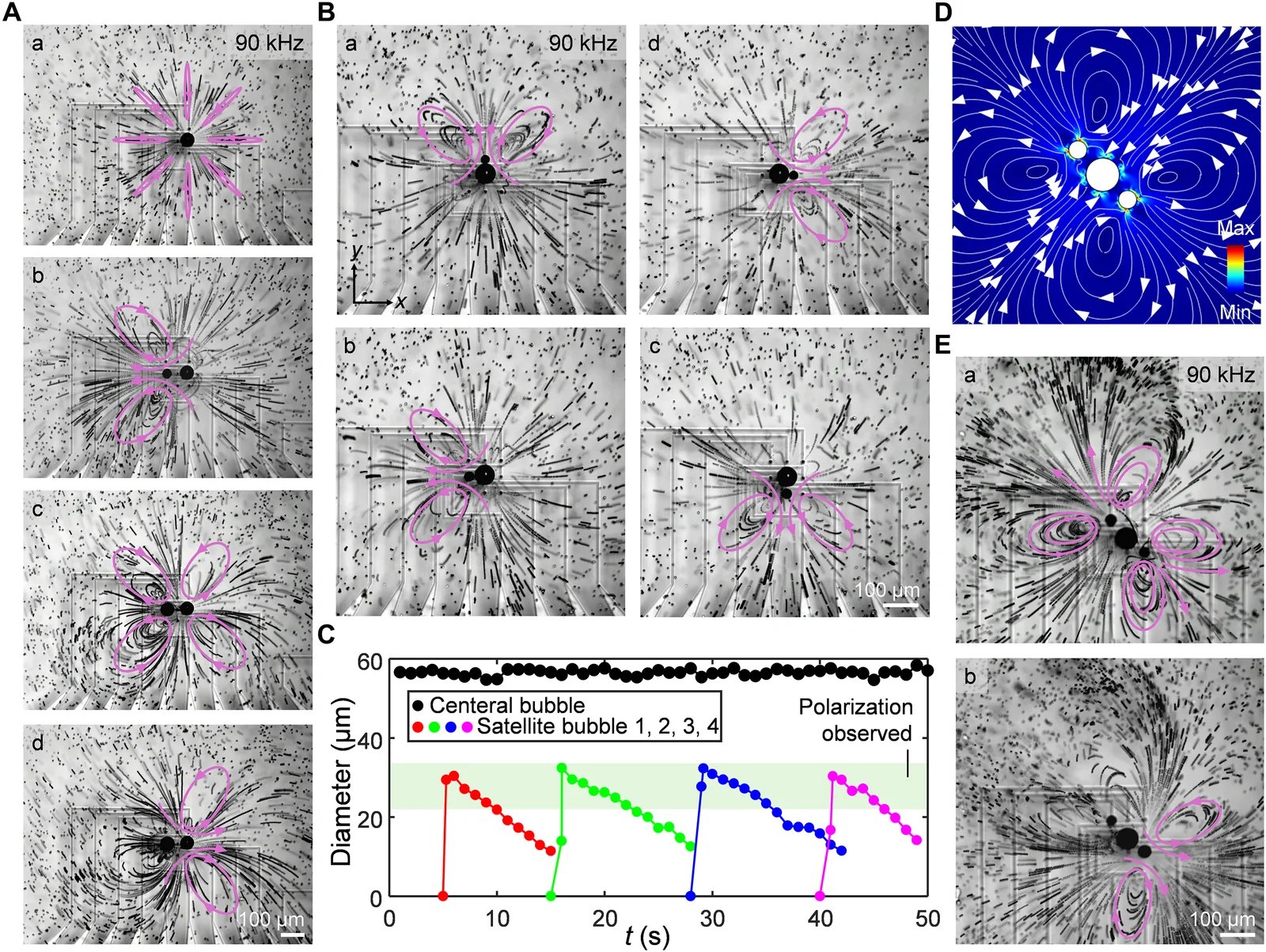
Controlled bubble-pair interaction and flow polarization. (**A**) Dynamic flow modulation by gradually increasing the size of the left bubble, with the left bubble being (a) absent, (b) smaller than, (c) equal in size to, and (d) larger than the right bubble. (**B**) Polarized flow control achieved by generating a smaller satellite bubble around a central bubble at different angular positions, with the satellite bubble located (a) above, (b) to the left of, (c) below, and (d) to the right of the central bubble. (**C**) Temporal evolution of bubble diameters under ultrasound excitation. The larger central bubble remained relatively stable over 50 s of ultrasound exposure. Smaller bubbles were rapidly generated within 1 to 2 s under electrolysis and subsequently shrank within 15 to 20 s. The light-green shaded region highlights the size range of satellite bubbles that induce flow polarization. (**D**) Numerical simulation of a three-bubble system, in which two satellite bubbles with comparable sizes produce a symmetric flow pattern. The color bar denotes normalized flow speed. (**E**) Experimental demonstrations corresponding to (D). (a) Symmetric flow generated when two satellite bubbles were similarly sized. (b) Flow polarization toward the bottom right direction when the top left bubble became too small to effectively couple with the central bubble. Flow fields were visualized using 5-μm-diameter tracer particles and superimposed frames, with magenta arrows indicating flow directions. All experiments were conducted at 90 kHz ultrasound with driving voltages of 7 V_pp_.

We then demonstrated dynamic polarization-direction modulation of acoustic streaming by generating smaller satellite bubbles (30 ± 2 μm) around a central bubble (≈57 μm) in four different directions (Fig. 3B and movie S4). In each case, the streaming was polarized from the central bubble toward the satellite bubble. Therefore, with the paired “center-satellite” bubbles, we can create flow with precise and reversible direction control on demand. Notably, the central bubble remained stable, while the satellite bubbles shrank due to the high Laplace pressure imbalance and limited contact pinning (*47*, *68*). Thus, we observed that the flow polarization gradually disappeared as the satellite microbubbles shrank. The captured size ratio change of bubble pairs showed that the fluid polarization is most prominent when the bubble-size ratio (smaller bubble/larger bubble) is 0.4 to 0.6 (Fig. 3C). This regime was determined by quantifying the polarized-flow speed using the average optical-flow speed within a selected region of interest. In this regime, the satellite bubble oscillates strongly enough to contribute appreciably to the coupled flow field, while remaining sufficiently different in size from the central bubble to preserve an amplitude-phase asymmetry. When the satellite bubble is too small, its oscillation amplitude and corresponding contribution to the flow field are too weak to substantially modify the overall streaming pattern. By contrast, as the satellite bubble approaches the size of the central bubble, its oscillation amplitude becomes substantial, but the phase difference between the two bubbles approaches 180°, resulting in a symmetric streaming pattern containing four vortices. It is worth noting that this range of size ratios does not represent a universal constant applicable to all bubble pairs. Instead, the occurrence of strong flow polarization depends sensitively on conditions such as bubble size, inter-bubble distance, bubble oscillation modes, and the local acoustic field. Detailed flow-speed quantification and additional examples with different bubble-size ratios are provided in note S2.

This flow polarization principle generalizes to *n* satellite bubbles. For example, in a triple-bubble system, simulations predicted that when two smaller bubbles are generated symmetrically on opposite sides of the central bubble, the system produces four symmetric vortices due to the two polarization contributions on either side (Fig. 3D). Experiments verified the prediction (Fig. 3E, a) and further revealed that the flow polarization could be switched to the opposite side when one of the satellite bubbles became too small to couple to the central bubble (Fig. 3E, b, and movie S4). More bubble interaction systems were simulated and provided in fig. S8. In summary, our platform transforms previously difficult-to-control coupled-bubble dynamics into a predictable, programmable, and scalable flow field primitive, where fluid polarization and vortex topology can be customized on demand.

### Active bubble oscillation decoupling and flow programming

To demonstrate selective oscillations and active decoupling of microbubbles, we implemented two control strategies: (i) resonance detuning by controlling bubble size and (ii) controlling the number of bubbles. In the first experiment (Fig. 4A and movie S5), initially, two similarly sized bubbles (90 and 82 μm) both responded strongly to ultrasound at 45 kHz and 10 V_pp_ due to their similar resonant frequencies. As we actively enlarged the lower bubble to 120 μm, its resonant frequency shifted away from the driving frequency. This modulation strongly suppressed its oscillation and reduced the effective coupling between the two oscillators, which altered the associated streaming. In the second experiment (Fig. 4B and movie S5), initially, two microbubbles with distinct diameters (128 and 87 μm) were respectively excited at 30 and 45 kHz due to a relatively large volume difference. As no bubbles were present on the remaining electrodes, fundamentally, no cross-excitation occurred on these sites. By applying −3.3 V electrolysis at the third electrode, a new bubble with a smaller diameter (35 μm) was generated. This bubble resonated at 79 kHz and introduced an additional, independently addressable streaming site in this system, while the fourth electrode remained inactive. These experiments highlight the ability of the platform to actively regulate bubble sizes and numbers, enabling selective mitigation of undesired cross-excitation and effective control over bubble interactions under the applied acoustic field.

**Fig. 4.**
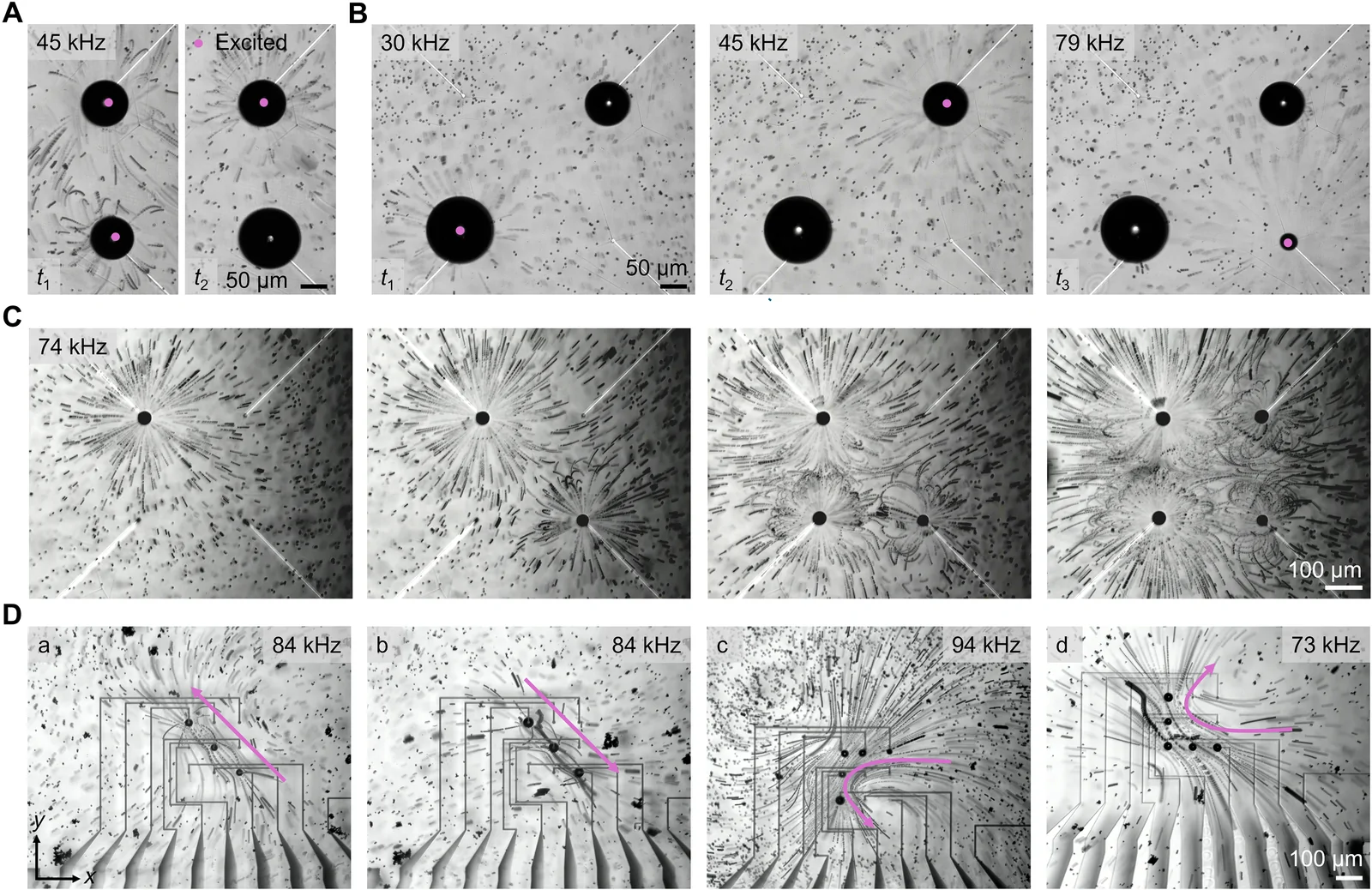
Active bubble oscillation decoupling and flow programming. (**A**) Active oscillation decoupling achieved by dynamically tuning the size of the bottom bubble in real time. Pink dots indicate the excited microbubbles. *t_i_* indicates different time points. (**B**) Active oscillation decoupling realized by controlling the number of bubbles over time. (**C**) Sequential generation and excitation of similarly sized microbubbles at different spatial locations, resulting in dynamic changes in the local flow field. (**D**) Programmable flow topologies corresponding to different microbubble arrangements: (a) a composite flow directed diagonally from the bottom right to the top left, (b) a flow from the top left to the bottom right, (c) a bottom-turning flow, and (d) a top-turning flow. Flow fields were visualized using 5-μm-diameter tracer particles and superimposed frames, with magenta arrows indicating flow directions. All experiments were conducted using 10 V_pp_ ultrasound at the corresponding driving frequencies.

To further show the control of microbubbles and program their resulting acoustofluidic fields, we demonstrated synchronous oscillations of multiple bubbles with a similar size. Four comparable bubbles (38 ± 2 μm) were sequentially generated at different positions and excited under a constant ultrasound at 74 kHz and 10 V_pp_. Dynamic changes in local acoustic streaming were enabled, where additional flow bifurcations were generated as bubbles were added (Fig. 4C and movie S6).

It is envisioned that expanding the electrode array will broaden the range of programmable flow functions. For example, we demonstrated various flow topologies with different bubble arrangements using a 3 × 3 electrode array with 100-μm pitch. Three similarly sized bubbles (≈27 μm in diameter) developed a composite flow from the bottom right diagonally to the top left at 84 kHz and 10 V_pp_ (Fig. 4D, a, and movie S7). Notably, increasing the bubble diameter to ≈37 μm reversed the flow direction under identical ultrasound excitation (Fig. 4D, b). We found that the formation and reversal of the composite flow are governed by the same peristaltic mechanism underlying the flow polarization and reversal in bubble pairs. Because the three bubbles are closely spaced, each bubble is driven not only by the incident ultrasound field but also by the secondary acoustic fields scattered by its neighboring bubbles. This mutual acoustic coupling modifies their relative oscillation phases, producing a collective response rather than three independent bubble oscillations. When the three bubbles are relatively small, their collective oscillations cause the associated flow fields to superpose constructively, generating a net composite flow along the diagonal direction. As the bubble diameter increases, the resonant frequency shifts downward, moving the bubble array into a different detuning regime. The resulting collective state alters the relative oscillation phases and swaps the phase-leading and phase-lagging relationships among the bubbles. This reorganization changes the spatial superposition of the flow fields and reverses the net composite flow along the opposite diagonal direction. In another experiment, an L-shaped bubble configuration generated a turning flow (Fig. 4D, c), and flipping the L-shaped arrangement, the turning direction flipped accordingly (Fig. 4D, d). We demonstrated expanded control over bubble oscillations and flow programming, including the selective excitation of bubbles of different sizes, the simultaneous excitation of similarly sized bubbles arranged in distinct spatial configurations, and programmable fluid pumping, vortex generation, and flow reorientation, in fig. S9. By expanding the range of manipulation strategies and further increasing the number of electrodes, diverse, dynamic, and reconfigurable acoustofluidic topologies are demonstrated in fig. S10.

Last, to enable on-demand removal of microbubbles, we developed two complementary strategies. First, global erasure was achieved by increasing the ultrasound excitation voltage to drive a strong oscillation (fig. S11 and movie S8). The enhanced radiation forces among adjacent bubbles promote their depinning and detachment from the electrode. Then, to selectively remove an individual bubble, we activated the corresponding electrode to initiate localized bubbling (fig. S12 and movie S8). A targeted bubble was removed while surrounding bubbles remained stably oscillating. Collectively, the platform allows us to both add and remove bubbles with equal precision.

### Directional bubble shooting and long-range fluid transport

Beyond microbubble removal, bubbling also serves to provide multiple bubbles for high-throughput manipulation. We further found that these floating bubbles could be periodically ejected and guided by ultrasound. When 74 kHz and 20 V_pp_ ultrasound was applied, the floating bubbles transitioned from a passive floating state to an active shooting state oriented in the *x-y* plane, which produced a directional net flow to the left (Fig. 5A). The shooting flow persisted over distances >2 mm. Tuning the ultrasound excitation frequency switched the shooting direction. For example, when we switched the ultrasound frequency to 90 kHz, the bubble shooting direction instantly shifted to the top left. Across the accessible frequency range of 0 to 100 kHz, we identified several distinct bubble-shooting directions, each corresponding to a different orientation angle relative to the electrode (Fig. 5A; see movies S9 and S10 for clear time-resolved visualization of the bubble-shooting trajectories). We quantified the frequency-selective directionality of bubble shooting using a polar representation, which shows that the shooting direction remains reproducible with a narrow angular spread at a given excitation frequency (Fig. 5B). We found that the flow velocity was sensitive to both the driving voltage for the PZT and the electrolysis voltage (Fig. 5, C and D). We measured a maximum average flow velocity of 625 μm/s at a distance of 100 μm in front of the electrode under 20 V_pp_ ultrasound and −10 V electrolysis (Materials and Methods). We were also able to capture the speed of shooting bubbles and found a maximum of ≈6,208 μm/s at 20 V_pp_ (inset in Fig. 5C). In addition, we determined that the bubbling rate reached ≈37 bubbles per second at −10 V (inset in Fig. 5D).

**Fig. 5.**
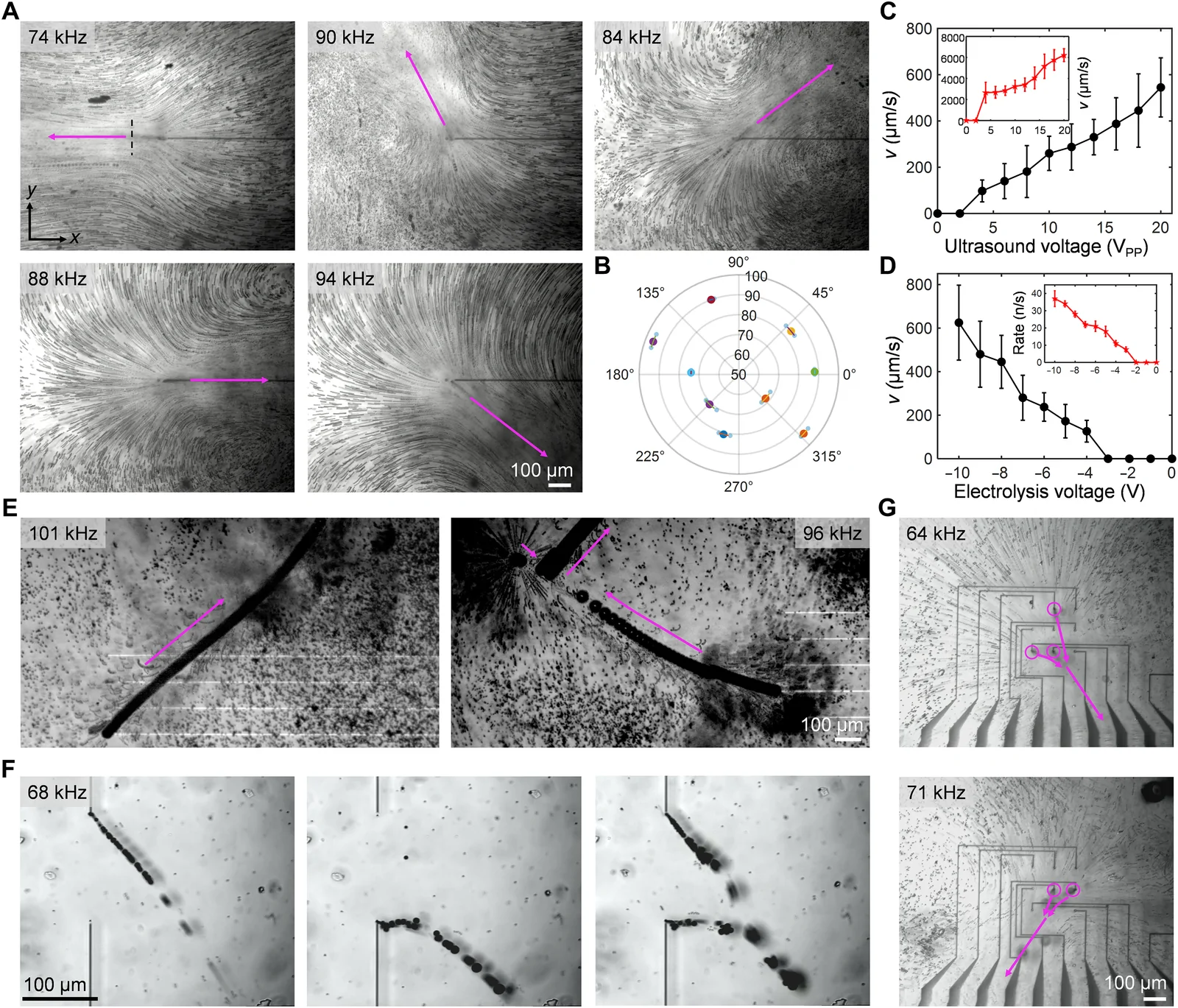
Directional bubble shooting and long-range fluid transport. (**A**) Directional bubble shooting achieved by switching the ultrasound excitation frequency under 20 V_pp_ ultrasound and −5 V electrolysis. Magenta arrows are overlaid on the bubble-shooting trajectories and indicate the corresponding shooting directions. (**B**) Frequency-selective bubble-shooting direction. The angular coordinate denotes the measured bubble-shooting direction, and the radial coordinate denotes the ultrasound excitation frequency. (**C**) Flow speed as a function of ultrasound excitation voltage at 74 kHz and −10 V electrolysis. Inset: Bubble shooting speed as a function of ultrasound excitation voltage. (**D**) Flow speed as a function of electrolysis voltage at 74 kHz and 20 V_pp_ ultrasound. Inset: Bubble generation rate as a function of electrolysis voltage. Error bars represent mean ± SD (*n* = 6 independent measurements). (**E**) Superimposed images of directional bubble shooting recorded using a high-speed camera at 1000 fps. (**F**) Selective bubble shooting enabled by an electrode pair under 10 V_pp_ ultrasound and (**G**) collective bubble shooting enabled by an electrode array under 20 V_pp_ ultrasound, with −5 V electrolysis. Flow fields were visualized using 5-μm-diameter tracer particles and superimposed frames, with magenta arrows indicating flow directions.

We used high-speed videos at 1000 frames per second to provide greater insight into the bubble shooting process (Fig. 5E and movie S11). The superimposed images revealed a repeatable shooting cycle: a microbubble gradually generated from the electrode during continuous electrolysis; meanwhile, the external ultrasound drove its oscillation. As the bubble grew, its oscillation amplitude intensified until it eventually detached from the electrode and shot away. After a bubble shot away, a new bubble formed and underwent the same cycle. Under constant ultrasound and electrolysis conditions, each newly generated bubble grew to approximately the same size as its predecessor. Thus, they experienced a similar acoustic radiation force and consequently shot in the same direction. Notably, under a different ultrasound excitation frequency, bubbles of a different equilibrium size were generated and shot along a new trajectory (Fig. 5E, right panel). In addition, when the moving bubble approached a previously formed bubble, the two bubbles coalesced into a larger bubble, leading to a pronounced change in the trajectory of the newly formed bubble.

On the basis of the experimental observations, force analyses, and simulations, we found a coupled field-resonance mechanism underlying frequency-selective bubble shooting. Before bubble detachment, electrochemical gas generation, capillary retention, contact-line pinning, buoyancy, and acoustic forcing jointly determine the bubble detachment timing and size. Following detachment, bubble motion is primarily governed by the total time-averaged acoustic radiation force. The lateral motion of the bubble is primarily determined by the primary Bjerknes force, which can be decomposed into pressure-gradient and phase-gradient components (see Materials and Methods). Thus, the primary Bjerknes force is governed jointly by the spatial acoustic field and the bubble resonance condition, rather than by either term alone. Varying the excitation frequency alters two aspects of the force coupling: It modifies the acoustic field via substrate vibration modes (fig. S13) and influences bubble response behavior by changing the resonant detuning state and bubble detachment size. These coupled changes in wave propagation, pressure gradients, and bubble oscillation phase and amplitude determine the relative dominance of the pressure-gradient and phase-gradient force components, which can rotate or reverse the direction of the lateral radiation force and produce frequency-selective bubble shooting. In addition, the wall-mediated secondary Bjerknes force confines the bubble near the substrate by counteracting buoyancy. Last, a secondary Bjerknes force arising from neighboring bubbles further facilitates mutual attraction, aggregation, and coalescence due to the change in volume and resonance characteristics. Hydrodynamic drag balances the resultant force during motion (see note S3 for detailed theoretical understanding and simulations).

Last, we investigated the selective and collective bubble shooting using paired electrodes and electrode arrays. Under similar ultrasound and electrolysis conditions, each electrode produced bubbles that shot in a similar direction, either independently or collectively (Fig. 5F and movie S12). Upon switching the excitation frequency, bubbles generated from all electrodes simultaneously changed their shooting direction, confirming synchronized frequency-selective control across the array (Fig. 5G). Consequently, this controllable, fast, and high-throughput bubble shooting capacity establishes a distinct functionality of our platform for long-range flow manipulation beyond the footprint of the electrode array.

### Potential applications

The demonstrated programmable flow topologies establish that this platform can generate a set of reconfigurable acoustofluidic primitives for microscale actuation and manipulation. Specifically, these flow primitives enable functions such as trapping, pumping, turning, polarization, and long-range transport, which constitute central operations in lab-on-chip systems. Because these functions are defined by reconfigurable bubble patterns rather than fixed channel geometries, the platform may be assembled into higher-level workflows for particle/cell routing, sorting, and localized sample processing. The flow polarization generated by paired center-satellite bubbles extends the platform toward acoustically powered microscopic robotic systems, in which electronically selected bubble configurations could provide an additional degree of control over force generation and flow direction under external ultrasound excitation. This reconfigurability may offer greater design space and adaptability than approaches in which actuation characteristics are fixed by the device structure.

To further advance the functional scope of bubble shooting, we experimentally demonstrated several application-relevant operations. First, directional bubble shooting removed surface-deposited particles and routed particles over long distances by sequentially switching the ultrasound excitation frequency, demonstrating its utility for localized cleaning and cargo transport (fig. S14). Then, by integrating the electrode array with a two-photon–polymerized microcapsule, controllable bubble generation and shooting could enable directional drug release and on-demand chemical reactions (fig. S15). These results show that programmable bubble oscillation and shooting are not merely tracer-particle visualization patterns, but functional microscale actuation mechanisms with potential utility in lab-on-chip manipulation, microscopic robotic propulsion, and future biomedical micro-tools.

## DISCUSSION

As these demonstrations show, we have developed a new electronic platform that enables real-time microbubble reconfiguration for diverse ultrasound actuation functions. The platform combines structural simplicity with rapid response and outstanding control capabilities. By tuning the electrolysis voltage, single-bubble generation and bubbling can be switched on demand. Then, under ultrasound excitation, we achieved a diverse range of bubble modulations, bubble interactions, and bubble shooting behaviors, with corresponding fluid manipulations. As such, by enabling the real-time electronic writing, erasing, and reconfiguration of bubbles at selected locations, our platform addresses several key challenges encountered by state-of-the-art microbubble systems, including limited bubble life span, fixed bubble position and size, excitation coupling, and an inability to reconfigure bubbles for excitation. The strategy we have adopted is inherently scalable, because incorporating additional electrodes and expanding the ultrasound parameter space would further increase the operational degrees of freedom and control granularity. Collectively, this platform represents a paradigm shift in ultrasound-bubble technologies from systems where bubbles are passively captured to ones where bubbles can be actively reconfigured on demand.

To quantitatively characterize the stability, repeatability, and durability of the active bubble-customization platform, we measured bubble-size evolution after electrolysis was discontinued and conducted image-based closed-loop bubble-size control, bubble-configuration regeneration, and repeated write/erase cycling tests (see note S4 for details). Postgeneration tracking showed that larger bubbles remained stable over extended timescales, whereas smaller bubbles shrank rapidly, primarily because of gas dissolution and the interfacial pressure imbalance. The image-based feedback experiments further demonstrated that bubble shrinkage could be dynamically compensated by applying corrective electrolysis pulses, thereby maintaining the target bubble diameter during operation. The relevant operational lifetime is therefore no longer governed solely by the passive dissolution time of an individual bubble, but by the ability of the electrode array to reproducibly regenerate and maintain the programmed bubble configuration over an extended period. Regeneration experiments showed that the desired bubble configurations could be reliably reproduced on the same device, producing highly consistent flow patterns across successive cycles. Last, repeated cycling tests confirmed that both the write/global-erase and write/selective-erase protocols could be operated reliably over numerous cycles under the tested conditions. Consequently, these results demonstrate that the platform enables sustained bubble customization and repeated acoustofluidic reconfiguration.

Despite the broad capabilities and promising results of our platform, several limitations and practical challenges remain. In the current implementation, the electrode array is externally wired and driven by benchtop instrumentation, which increases system complexity and may constrain deployment in space-limited settings. In future iterations, standardized and encapsulated packaging strategies could be adopted to facilitate integration with complementary metal-oxide semiconductor–compatible chips and microelectronic circuits for portable or wireless implementations (*69*, *70*). At present, bubble reconfiguration is demonstrated in two dimensions, as standard photolithography primarily enables planar electrode arrays. By extending the electrode structure into three dimensions using emerging three-dimensional (3D) microfabrication and micro-actuation techniques (*71*–*73*), 3D bubble arrangements and flow topologies could be achieved (*74*, *75*). For bubble shooting, the frequency-direction map is sensitive to chamber geometry, boundary conditions, transducer coupling, and substrate vibration. Therefore, approaches that use robust ultrasound generation methods, such as surface acoustic wave devices or acoustic holographic platforms, will likely be required to obtain prescribed and stable acoustic fields.

## MATERIALS AND METHODS

### Fabrication of microelectrodes

Fabrication of the electrode-array chip involves two material layers. These include, from bottom to top, growing and patterning the electrode layer and etching the insulation layer. The detailed recipe is as follows.

#### Electrode layer

We started the fabrication on a fused-silica wafer [4 inches (100 mm), Nova]. A negative photoresist (MicroChemicals NLOF 2020) was spin coated and patterned. Titanium (≈10 nm) and platinum (≈60 nm) were sputtered at 3 mtorr and 400 W (AJA sputter tool). The photoresist was then stripped with Microposit Remover 1165 under sonication.

#### Insulation layer

SiO_2_ (1 to 2 μm) was grown using Oxford 100 PECVD. A negative photoresist (NLOF 2020) was spin coated and patterned. The SiO_2_ was then etched using CHF_3_/O_2_ oxide etching (Oxford 80+ etcher). Last, the photoresist was stripped in Microposit Remover 1165. For SU-8 insulation, SU8-5 (2 to 8 μm) was spin coated and patterned.

### Experimental setup

Electrolysis at the electrodes was controlled by a field-programmable gate array (FPGA; DE2-115, Terasic) with tunable voltages (−3.3 and −5 V) and a function generator (SDG-1032X, Siglent) with dc outputs from 0 to −10 V. For the acoustic setup, a circular piezoelectric transducer (10 mm by 0.1 mm in diameter and thickness, PUI Audio) was affixed to the substrate using epoxy resin (2-K-Epoxidkleber, UHU Schnellfest). A cylindrical PDMS acoustic chamber (10 mm by 3 mm by 2 mm in inner diameter, height, and thickness) was bonded atop the electrodes and filled with 1× PBS (pH 7.4, Gibco). The center-to-center distance between the transducer and the acoustic chamber was 13 mm. During experiments, the substrate was fixed on the operation stage of an optical microscope (WF, Mitutoyo and Alessi Rel-4800, Cascade Microtech). Ultrasound excitation signals were generated using a function generator (SDG-1032X, Siglent) with tunable frequencies from 0 to 30 MHz and amplitudes up to 20 V_pp_. The peak-to-peak voltage was directly applied to the transducer.

### Microbubble generation efficiency

Microbubble generation was characterized using electrodes of varying exposed diameters (2 to 10 μm, 2-μm increments) and cavity heights (1 to 8 μm, 1-μm increments). At an electrolysis voltage of −3.3 V and a 6-μm exposed diameter, the bubble volume exhibited a linear dependence on electrolysis time (fig. S1C) consistent with Faraday’s law of electrolysis (*54*)

$$V=\frac{\mathrm{ItM}}{zF\rho }$$ (1)

where *V* is the total gas volume generated, *I* is the electric current (in amperes), *t* is the electrolysis time (in seconds), *M* is the molar mass of the gas, *z* is the number of electrons transferred, *F* is Faraday’s constant (96,485 C/mol), and ρ is the gas density.

On the basis of the measured bubble-volume growth rate and numerical fitting, we estimated the average electrolysis current to be *I* ≈ 471 nA. To verify this estimation experimentally, we measured the electrolysis current using an electrochemical workstation (Reference 620, Gamry instrument). The working-electrode voltage was set to −3.3 V for electrolysis durations ranging from 1 to 10 s, with an increment of 1 s. The current was recorded at a sampling interval of 0.01 s. After each electrolysis step, the working-electrode voltage was switched back to 0 V for 1 s before the next measurement. As shown in fig. S16A, the measured electrolysis current remained relatively stable, with an average value of 481.5 ± 34.2 nA. This value agrees with the current estimated from Faraday’s law. We also measured electrode arrays from several different fabrication batches. At −3.3 V, these devices exhibited average electrolysis currents ranging from approximately 300 to 600 nA. The variations among devices may arise from differences in the thickness, roughness, and quality of the metal films deposited by magnetron sputtering, as well as differences in the insulation quality. These device-to-device variations can be calibrated through bubble-generation experiments before use, allowing the bubble size to be reproducibly programmed by controlling the electrolysis time. In addition, we measured the electrolysis current under different electrolysis voltages. As shown in fig. S16B, the current magnitude increased substantially as the applied voltage magnitude increased. When the voltage magnitude exceeded approximately 4 V, the current magnitude became larger than 1.4 μA, corresponding to the bubbling regime observed experimentally. In contrast, when the applied voltage was −1 or −2 V, the current magnitude was below 1 nA, which was insufficient to generate observable bubbles. Therefore, the electrolysis-current measurements are consistent with the bubble-generation behavior observed in the experiments.

We then found that cavity height influenced the transition between single bubble generation and bubbling. Heights of 1 to 2 μm enabled repeated switching between the two states by adjusting electrolysis voltages. When the height was greater than 3 μm, it favored stable single bubble generation. At lower heights (<1 μm), it was conducive to bubbling due to the relatively weaker pinning force.

### Resonant frequency characterization

As shown in fig. S1D, the resonant frequency decreased from 100 to 1 kHz with increasing bubble diameters, consistent with the inverse scaling Minnaert relationship between the bubble’s natural frequency and its size (*55*)

$$f=\frac{1}{2\pi R}\sqrt{\frac{3\gamma P_{0}}{\rho_{L}}}$$ (2)

where *R* is the radius of the microbubble, γ is the adiabatic index of the gas inside the bubble (≈1.41 for H_2_), *P*_0_ is the ambient pressure (typically atmospheric pressure: ≈101,325 Pa), and $\rho_{L}$ is the density of the surrounding liquid (1000 kg/m^3^ for water, ≈1003 kg/m^3^ for 1× PBS buffer).

### Acoustic radiation forces

For a subwavelength bubble whose response is dominated by spherical volumetric oscillations, the leading-order translational force exerted on the bubble from the external acoustic field can be approximated by the primary Bjerknes force (*67*), which is expressed schematically as

$$F_{B}^{\text{pri}}=-\langle V\left(t\right)\;\nabla p(x_{b},t)\rangle =-\frac{1}{2}\text{Re}\left[\hat{V}{\left(\nabla {\hat{p}}_{0}\right)}^{*}\right]$$ (3)

where *V*(*t*) is the instantaneous bubble volume, ∇p is the acoustic pressure gradient, $x_{b}$ is the bubble position, and 〈⋅〉 denotes the time averaging over one acoustic cycle. $p_{0}$ is the external acoustic pressure evaluated at the bubble position, and excluding the target bubble’s self-field, a hat denotes a complex harmonic amplitude, and an asterisk denotes complex conjugation. In this framework, bubble resonance determines the phase and amplitude of the bubble-volume oscillation *V*(*t*), whereas the acoustic field in the chamber determines the local pressure-gradient field $\nabla p\left(x_{b},t\right)$. Therefore, the primary Bjerknes force is governed by both the bubble resonance condition and the spatial distribution of the acoustic field.

By writing $\hat{p_{0}}={\mathrm{Ae}}^{\mathrm{i\phi}},\hat{V}=\left|\hat{V}\right|e^{i\phi V}$ and Δϕ=ϕV−ϕ, the complex form of $F_{B}^{\text{pri}}$ can be decomposed into a pressure-gradient contribution and a phase-gradient contribution (*76*, *77*)

$$F_{B}^{\text{pri}}=-\frac{|\hat{V}|}{2}\text{cos}\left(\Delta \phi \right)\;\nabla A-\frac{|\hat{V}|}{2}\text{sin}\left(\Delta \phi \right)\;A\nabla \phi$$ (4)

The secondary Bjerknes force acting on the bubble due to the scattered acoustic field generated by a neighboring bubble and the wall-mediated image bubble can be expressed schematically as

$$F_{B}^{\text{sec}}=-\langle V_{1}\left(t\right)\;\nabla p_{2}(x_{1},t)\rangle \approx \frac{\rho_{0}}{4\pi d^{2}}\langle {\dot{V}}_{1}\left(t\right)\;{\dot{V}}_{2}\left(t\right)\rangle e_{12}$$ (5)

where $p_{2}\left(x_{1},t\right)$ is the scattered acoustic pressure generated by bubble 2 and evaluated at the position of bubble 1, *d* is the center-to-center distance between the two bubbles, and **e**_12_ is the unit vector pointing from bubble 1 to bubble 2 (*78*–*80*).

### Bubble shooting characterization

Figure 5C shows that both the flow and bubble shooting speeds increased as the ultrasound driving voltage increased from 4 to 20 V_pp_, at −10-V electrolysis. However, no noticeable shooting occurred below 4 V_pp_. Notably, the bubble shooting speed is 4.85 to 11.39 times the associated flow speed, reflecting the nonlinear rise with acoustic amplitude. Similarly, raising the electrolysis voltage from −4 to −10 V (at 20-V_pp_ ultrasound) increased the flow velocity and bubble production rate (Fig. 5D). No bubbling was observed when the electrolysis voltage magnitude was below 4 V. Therefore, under 20-V_pp_ ultrasound and −10-V electrolysis, a maximum average flow velocity of 625 μm/s was achieved, with a low Reynolds number of *Re* ≈ 3.1 × 10^−3^ for 5-μm tracer particles. Meanwhile, the maximum average bubble shooting speed reached 6208 μm/s, corresponding to *Re* ≈ 0.19 to 0.62 for bubble diameters between 30 and 100 μm, remaining within the laminar flow regime.

### Numerical simulations

Finite-element simulations were conducted using COMSOL Multiphysics, including pressure acoustics, thermoviscous acoustics, laminar flow, solid mechanics, electrostatics, and heat transfer in solids and fluids. The configurations used to simulate acoustic streaming and bubble-oscillation phases are provided in COMSOL Output Report S1. The configurations used to simulate substrate vibration, acoustic pressure, and thermal distribution are provided in COMSOL Output Report S2. The configurations used to simulate prescribed acoustic fields and acoustic radiation forces are provided in COMSOL Output Report S3. All simulations were performed using geometric dimensions and material properties based on those used in the experiments. Bubble size, bubble number, and acoustic-field parameters were varied in parametric sweeps.

### Imaging and analysis

Bubble dynamics and flows were recorded using a camera (30 fps; a2A2448-75ucPRO, Basler) and a high-speed camera (1000 fps; VEO 710L, Phantom). Recorded footage was analyzed in ImageJ. Flows were visualized using uniform polymer tracer particles with a diameter of 5 μm (Duke Standards).

## Supplementary Materials

### The PDF file includes:

Notes S1 to S4 Figs. S1 to S31 Legends for movies S1 to S12 Legends for COMSOL Output Reports S1 to S3 References

## References

[1] Z. Miao, T. Feng, A. Ren, H. Huang, S. Li, Y. Xue, Y. Shen, X. Liu, Z. Chen, P. Jin, T.-W. Wong, F. Zhou, T. Li, X. Yang, W. Yang, Acoustically powered micro-clampbot for single-particle transportation. Sci. Adv. 11, eady3213 (2025). 10.1126/sciadv.ady3213 PMC13155591 40991711

[2] M. Kaynak, A. Dolev, M. S. Sakar, 3D printed acoustically programmable soft microactuators. Soft Robot. 10, 246–257 (2023). 10.1089/soro.2021.0193 PMC10123809 35704862

[3] T. Qiu, F. Adams, S. Palagi, K. Melde, A. G. Mark, U. Wetterauer, A. Miernik, P. Fischer, Wireless acoustic-surface actuators for miniaturized endoscopes. ACS Appl. Mater. Interfaces 9, 42536–42543 (2017). 10.1021/acsami.7b12755 PMC5730945 29148713

[4] N. Mahkam, M. C. Ugurlu, S. K. Kalva, A. Aghakhani, D. Razansky, M. Sitti, Multiorifice acoustic microrobot for boundary-free multimodal 3D swimming. Proc. Natl. Acad. Sci. U.S.A. 122, e2417111122 (2025). 10.1073/pnas.2417111122 PMC11789062 39841149

[5] C. Dillinger, A. Rasaiah, A. Vogel, C. Bahou, K. Monastyrskaya, A. H. Gheinani, D. Ahmed, Real-time color flow mapping of ultrasound microrobots. Sci. Adv. 11, eadt8887 (2025). 10.1126/sciadv.adt8887 PMC12273784 40680140

[6] L. Chai, D. Kim, S. K. Chung, K. Y. Lee, Magnetically maneuverable micromixer using acoustically excited bubbles. J. Micromech. Microeng. 35, 035001 (2025).

[7] H. Han, X. Ma, W. Deng, J. Zhang, S. Tang, O. S. Pak, L. Zhu, E. Criado-Hidalgo, C. Gong, E. Karshalev, J. Yoo, M. You, A. Liu, C. Wang, H. K. Shen, P. N. Patel, C. L. Hays, P. J. Gunnarson, L. Li, Y. Zhang, J. O. Dabiri, L. V. Wang, M. G. Shapiro, D. Wu, Q. Zhou, J. R. Greer, W. Gao, Imaging-guided bioresorbable acoustic hydrogel microrobots. Sci. Robot. 9, eadp3593 (2024). 10.1126/scirobotics.adp3593 39661698

[8] J. Janiak, Y. Li, Y. Ferry, A. A. Doinikov, D. Ahmed, Acoustic microbubble propulsion, train-like assembly and cargo transport. Nat. Commun. 14, 4705 (2023). 10.1038/s41467-023-40387-7 PMC10404234 37543657

[9] L. Ren, N. Nama, J. M. McNeill, F. Soto, Z. Yan, W. Liu, W. Wang, J. Wang, T. E. Mallouk, 3D steerable, acoustically powered microswimmers for single-particle manipulation. Sci. Adv. 5, eaax3084 (2019). 10.1126/sciadv.aax3084 PMC6814402 31692692

[10] D. Ahmed, M. Lu, A. Nourhani, P. E. Lammert, Z. Stratton, H. S. Muddana, V. H. Crespi, T. J. Huang, Selectively manipulable acoustic-powered microswimmers. Sci. Rep. 5, 9744 (2015). 10.1038/srep09744 PMC4438614 25993314

[11] T. Luo, M. Wu, Biologically inspired micro-robotic swimmers remotely controlled by ultra-sound waves. Lab Chip 21, 4095–4103 (2021). 10.1039/d1lc00575h 34549766

[12] J. Jeong, D. Jang, D. Kim, D. Lee, S. K. Chung, Acoustic bubble-based drug manipulation: Carrying, releasing and penetrating for targeted drug delivery using an electromagnetically actuated microrobot. Sens. Actuators A Phys. 306, 111973 (2020).

[13] X. Liu, Y. Li, F. Liu, Q. Shi, L. Dong, Q. Huang, T. Arai, T. Fukuda, μSonic-hand: Biomedical micromanipulation driven by acoustic gas-liquid-solid interactions. Sci. Adv. 11, eads8167 (2025). 10.1126/sciadv.ads8167 PMC11952102 40153493

[14] Z. Zhang, L. K. Allegrini, N. Yanagisawa, Y. Deng, S. C. Neuhauss, D. Ahmed, Sonorotor: An acoustic rotational robotic platform for zebrafish embryos and larvae. IEEE Robot. Autom. Lett. 8, 2598–2605 (2023).

[15] D. Ahmed, A. Ozcelik, N. Bojanala, N. Nama, A. Upadhyay, Y. Chen, W. Hanna-Rose, T. J. Huang, Rotational manipulation of single cells and organisms using acoustic waves. Nat. Commun. 7, 11085 (2016). 10.1038/ncomms11085 PMC4814581 27004764

[16] R. Goyal, A. G. Athanassiadis, Z. Ma, P. Fischer, Amplification of acoustic forces using microbubble arrays enables manipulation of centimeter-scale objects. Phys. Rev. Lett. 128, 254502 (2022). 10.1103/PhysRevLett.128.254502 35802439

[17] T. Qiu, S. Palagi, A. G. Mark, K. Melde, F. Adams, P. Fischer, Wireless actuation with functional acoustic surfaces. Appl. Phys. Lett. 109, 191602 (2016).

[18] A. Marzo, B. W. Drinkwater, Holographic acoustic tweezers. Proc. Natl. Acad. Sci. U.S.A. 116, 84–89 (2019). 10.1073/pnas.1813047115 PMC6320506 30559177

[19] L. Wang, Y. Wu, Y. Tang, H. Wei, T. Lyu, X. Zhang, T.-Y. Huang, Reconfigurable acoustofluidic microvortices for selective microcargo delivery. Adv. Sci. 13, e21612 (2026). 10.1002/advs.202521612 PMC13042385 41517865

[20] M. Medany, L. Piglia, L. Achenbach, S. K. Mukkavilli, D. Ahmed, Model-based reinforcement learning for ultrasound-driven autonomous microrobots. Nat. Mach. Intell. 7, 1076–1090 (2025). 10.1038/s42256-025-01054-2 PMC12283351 40709099

[21] L. Meng, X. Liu, Y. Wang, W. Zhang, W. Zhou, F. Cai, F. Li, J. Wu, L. Xu, L. Niu, H. Zheng, Sonoporation of cells by a parallel stable cavitation microbubble array. Adv. Sci. 6, 1900557 (2019). 10.1002/advs.201900557 PMC6724477 31508275

[22] C. B. Dayan, N. Mahkam, N. O. Dogan, V. Liimatainen, D. Hiz, A. Ilgezdi, D. Razansky, A. Aghakhani, M. Sitti, Multiscale acoustic bubble actuators using bioinspired liquid-repellent microstructures. Adv. Mater. Technol. 11, e01532 (2026).

[23] C. Bai, Z. Chen, Y. Li, Y. Chen, Q. Shi, Q. Huang, T. Fukuda, T. Arai, X. Liu, Acoustic rising microbubbles for efficient liquid operations. Cyborg Bionic Syst. 7, 0449 (2026). 10.34133/cbsystems.0449 PMC12968397 41808671

[24] Z. Shi, Z. Zhang, J. Schnermann, S. C. Neuhauss, N. Nama, R. Wittkowski, D. Ahmed, Ultrasound-driven programmable artificial muscles. Nature 646, 1096–1104 (2025). 10.1038/s41586-025-09650-3 PMC12571877 41162719

[25] Y. Gao, M. Wu, Y. Lin, W. Zhao, J. Xu, Acoustic bubble-based bidirectional micropump. Microfluid. Nanofluid. 24, 29 (2020).

[26] Q. Tang, F. Liang, L. Huang, P. Zhao, W. Wang, On-chip simultaneous rotation of large-scale cells by acoustically oscillating bubble array. Biomed. Microdevices 22, 13 (2020). 10.1007/s10544-020-0470-1 31955256

[27] X. Li, Z. Deng, W. Zhang, W. Zhou, X. Liu, H. Quan, J. Li, P. Li, Y. Li, C. Hu, F. Li, L. Niu, Z. Tian, L. Meng, H. Zheng, Oscillating microbubble array-based metamaterials (OMAMs) for rapid isolation of high-purity exosomes. Sci. Adv. 11, eadu8915 (2025). 10.1126/sciadv.adu8915 PMC12002133 40238867

[28] S. Mohanty, A. Paul, P. M. Matos, J. Zhang, J. Sikorski, S. Misra, CeFlowBot: A biomimetic flow-driven microrobot that navigates under magneto-acoustic fields. Small 18, e2105829 (2022). 10.1002/smll.202105829 34889051

[29] K. Nguyen, A. Swain, Y. Zheng, A. Vasa, A. Jain, P. Phaneesh, M. Nouri, A. Kuo, “Optothermally driven bubble etching for sustainable and on-demand nanomanufacturing,” in *Optical Trapping and Optical Micromanipulation XXII* (SPIE, 2025), PC135800U.

[30] H. Joh, B. Lian, S.-i. Hsueh, Z. Ma, K.-J. Lee, S.-Y. Zheng, P. Fischer, D. E. Fan, Massively parallel microbubble nano-assembly. Nat. Commun. 16, 6743 (2025). 10.1038/s41467-025-62070-9 PMC12284250 40695847

[31] J. H. Shin, J. Seo, J. Hong, S. K. Chung, Hybrid optothermal and acoustic manipulations of microbubbles for precise and on-demand handling of micro-objects. Sens. Actuators B Chem. 246, 415–420 (2017).

[32] L. Lin, X. Peng, Z. Mao, W. Li, M. N. Yogeesh, B. B. Rajeeva, E. P. Perillo, A. K. Dunn, D. Akinwande, Y. Zheng, Bubble-pen lithography. Nano Lett. 16, 701–708 (2016). 10.1021/acs.nanolett.5b04524 PMC5490994 26678845

[33] W. Gao, J. Wang, Synthetic micro/nanomotors in drug delivery. Nanoscale 6, 10486–10494 (2014). 10.1039/c4nr03124e 25096021

[34] Q. Gao, F. Li, J. Gao, E. Zhang, Y. Su, N. Qin, H. Ye, J. Puigmartí, G. Cantarella, S. Schuerle, J. Wu, B. J. Nelson, S. Pané, Reconfigurable magnetic soft microrobot for acoustically triggered targeted bacterial sterilization. Adv. Funct. Mater. 36, e18017 (2026).

[35] D. Wang, Z. Liu, H. Zhao, H. Qin, G. Bai, C. Chen, P. Shi, Y. Du, Y. Zhao, W. Liu, D. Wang, G. Zhou, X. He, C. Dai, Launching by cavitation. Science 389, 935–939 (2025). 10.1126/science.adu8943 40875850

[36] M. Cattaneo, G. Guerriero, G. Shakya, L. A. Krattiger, L. G. Paganella, M. L. Narciso, O. Supponen, Cyclic jetting enables microbubble-mediated drug delivery. Nat. Phys. 21, 590–598 (2025). 10.1038/s41567-025-02785-0 PMC11999868 40248569

[37] X. Huang, P.-B. Liu, G.-Y. Niu, H.-B. Hu, Experimental study on the translation behavior of an in-situ bubble pair in the ultrasonic field. Ultrason. Sonochem. 112, 107188 (2025). 10.1016/j.ultsonch.2024.107188 PMC11697788 39671814

[38] M. U. Jung, Y. C. Kim, G. Bournival, S. Ata, Tailoring bubble size through acoustic-assisted microbubble generation. AIChE J. 71, e18802 (2025).

[39] H. J. Cho, J. P. Mizerak, E. N. Wang, Turning bubbles on and off during boiling using charged surfactants. Nat. Commun. 6, 8599 (2015). 10.1038/ncomms9599 PMC4639814 26486275

[40] C.-H. Choi, M. David, Z. Gao, A. Chang, M. Allen, H. Wang, C. H. Chang, Large-scale generation of patterned bubble arrays on printed bi-functional boiling surfaces. Sci. Rep. 6, 23760 (2016). 10.1038/srep23760 PMC4817132 27034255

[41] Y. Lee, V. K. Bandari, J. S. McCaskill, P. Adluri, D. Karnaushenko, D. D. Karnaushenko, O. G. Schmidt, Si chiplet-controlled 3D modular microrobots with smart communication in natural aqueous environments. Sci. Robot. 10, eadu6007 (2025). 10.1126/scirobotics.adu6007 40834063

[42] G. Zhang, S. Yang, J. F. Yang, D. Gonzalez-Medrano, M. Z. Miskin, V. B. Koman, Y. Zeng, S. X. Li, M. Kuehne, A. T. Liu, A. M. Brooks, M. Kumar, M. S. Strano, High energy density picoliter-scale zinc-air microbatteries for colloidal robotics. Sci. Robot. 9, eade4642 (2024). 10.1126/scirobotics.ade4642 39141708

[43] G. Wu, I. Heck, N. Zhang, G. Phaup, X. Zhang, Y. Wu, D. E. Stalla, Z. Weng, H. Sun, H. Li, Z. Zhang, S. Ding, D.-P. Li, Y. Zhang, Wireless, battery-free push-pull microsystem for membrane-free neurochemical sampling in freely moving animals. Sci. Adv. 8, eabn2277 (2022). 10.1126/sciadv.abn2277 PMC8865804 35196090

[44] Z. Ma, K. Melde, A. G. Athanassiadis, M. Schau, H. Richter, T. Qiu, P. Fischer, Spatial ultrasound modulation by digitally controlling microbubble arrays. Nat. Commun. 11, 4537 (2020). 10.1038/s41467-020-18347-2 PMC7484750 32913270

[45] K. H. Lee, J. H. Lee, J. M. Won, K. Rhee, S. K. Chung, Micromanipulation using cavitational microstreaming generated by acoustically oscillating twin bubbles. Sens. Actuators A Phys. 188, 442–449 (2012).

[46] S. K. Chung, Y. Zhao, S. K. Cho, On-chip creation and elimination of microbubbles for a micro-object manipulator. J. Micromech. Microeng. 18, 095009 (2008).

[47] A. Bashkatov, F. Bürkle, Ç. Demirkır, W. Ding, V. Sanjay, A. Babich, X. Yang, G. Mutschke, J. Czarske, D. Lohse, D. Krug, L. Büttner, K. Eckert, Electrolyte droplet spraying in H2 bubbles during water electrolysis under normal and microgravity conditions. Nat. Commun. 16, 4580 (2025). 10.1038/s41467-025-59762-7 PMC12084577 40379635

[48] L. Deng, L. Jin, L. Yang, C. Feng, A. Tao, X. Jia, Z. Geng, C. Zhang, X. Cui, J. Shi, Bubble evolution dynamics in alkaline water electrolysis. eScience 5, 100353 (2025).

[49] H. Tüysüz, Alkaline water electrolysis for green hydrogen production. Acc. Chem. Res. 57, 558–567 (2024). 10.1021/acs.accounts.3c00709 PMC10882964 38335244

[50] K. Matis, E. Peleka, Alternative flotation techniques for wastewater treatment: Focus on elec-troflotation. Sep. Sci. Technol. 45, 2465–2474 (2010).

[51] S. Boinpally, A. Kolla, J. Kainthola, R. Kodali, J. Vemuri, A state-of-the-art review of the electrocoagulation technology for wastewater treatment. Water Cycle 4, 26–36 (2023).

[52] M. Bharti, P. P. Das, M. K. Purkait, A review on the treatment of water and wastewater by electrocoagulation process: Advances and emerging applications. J. Environ. Chem. Eng. 11, 111558 (2023).

[53] F. Sepahi, R. Verzicco, D. Lohse, D. Krug, Mass transport at gas-evolving electrodes. J. Fluid Mech. 983, A19 (2024).

[54] X. Yang, F. Karnbach, M. Uhlemann, S. Odenbach, K. Eckert, Dynamics of single hydrogen bubbles at a platinum microelectrode. Langmuir 31, 8184–8193 (2015). 10.1021/acs.langmuir.5b01825 26133052

[55] M. Minnaert, XVI. On musical air-bubbles and the sounds of running water. Philos. Mag. 16, 235–248 (1933).

[56] M. Versluis, D. E. Goertz, P. Palanchon, I. L. Heitman, S. M. van der Meer, B. Dollet, N. de Jong, D. Lohse, Microbubble shape oscillations excited through ultrasonic parametric driving. Phys. Rev. E 82, 026321 (2010). 10.1103/PhysRevE.82.026321 20866919

[57] A. A. Doinikov, S. Cleve, G. Regnault, C. Mauger, C. Inserra, Acoustic microstreaming produced by nonspherical oscillations of a gas bubble. I. Case of modes 0 and *m*. Phys. Rev. E 100, 033104 (2019). 10.1103/PhysRevE.100.033104 31639916

[58] A. A. Doinikov, T. Combriat, P. A. Thibault, P. Marmottant, Acoustic streaming produced by a cylindrical bubble undergoing volume and translational oscillations in a microfluidic channel. Phys. Rev. E 94, 033109 (2016). 10.1103/PhysRevE.94.033109 27739843

[59] J. Mondal, Y. Wu, A. Mishra, F. Akbaridoust, I. Marusic, P. Ghosh, M. Ashokkumar, Bubble oscillations at low frequency ultrasound for biological applications. Ultrason. Sonochem. 104, 106816 (2024). 10.1016/j.ultsonch.2024.106816 PMC11639710 38433032

[60] V. Poulichet, A. Huerre, V. Garbin, Shape oscillations of particle-coated bubbles and directional particle expulsion. Soft Matter 13, 125–133 (2017). 10.1039/c6sm01603k PMC5304335 27714376

[61] G. Lajoinie, Y. Luan, E. Gelderblom, B. Dollet, F. Mastik, H. Dewitte, I. Lentacker, N. de Jong, M. Versluis, Non-spherical oscillations drive the ultrasound-mediated release from targeted microbubbles. Commun. Phys. 1, 22 (2018).

[62] N. A. Pelekasis, A. Gaki, A. Doinikov, J. A. Tsamopoulos, Secondary Bjerknes forces between two bubbles and the phenomenon of acoustic streamers. J. Fluid Mech. 500, 313–347 (2004).

[63] A.-M. Zhang, S.-M. Li, P. Cui, S. Li, Y.-L. Liu, A unified theory for bubble dynamics. Phys. Fluids 35, 033323 (2023).

[64] Y. Shen, L. Zhang, Y. Wu, W. Chen, The role of the bubble-bubble interaction on radial pulsations of bubbles. Ultrason. Sonochem. 73, 105535 (2021). 10.1016/j.ultsonch.2021.105535 PMC8050038 33823488

[65] F. Mekki-Berrada, T. Combriat, P. Thibault, P. Marmottant, Interactions enhance the acoustic streaming around flattened microfluidic bubbles. J. Fluid Mech. 797, 851–873 (2016).

[66] J. Jiao, Y. He, S. E. Kentish, M. Ashokkumar, R. Manasseh, J. Lee, Experimental and theoretical analysis of secondary Bjerknes forces between two bubbles in a standing wave. Ultrasonics 58, 35–42 (2015). 10.1016/j.ultras.2014.11.016 25542344

[67] T. G. Leighton, A. J. Walton, M. J. W. Pickworth, Primary Bjerknes forces. Eur. J. Phys. 11, 47–50 (1990).

[68] S. Park, L. Liu, Ç. Demirkır, O. van der Heijden, D. Lohse, D. Krug, M. T. M. Koper, Solutal Marangoni effect determines bubble dynamics during electrocatalytic hydrogen evolution. Nat. Chem. 15, 1532–1540 (2023). 10.1038/s41557-023-01294-y 37563325

[69] S. Lee, S. Ghajari, S. Sadeghi, Y. Zheng, H. Zahr, A. J. Cortese, W. Gu, K. Choe, A. Mok, M. Wallace, R. Jiao, C. Wu, J. C. Werth, W. Fan, P. Mogalipuvvu, J. U. Park, S. Zhao, C. Smart, T. A. Cleland, M. R. Warden, J. Lammerding, T. Wang, J. H. Goldberg, P. L. McEuen, C. Xu, A. C. Molnar, A subnanolitre tetherless optoelectronic microsystem for chronic neural recording in awake mice. Nat. Electron. 8, 1259–1271 (2025). 10.1038/s41928-025-01484-1 PMC12727521 41451321

[70] M. Taghavi, W. Wang, K. Shim, J. Zhang, I. Cohen, A. Apsel, Coordinated behavior of autonomous microscopic machines through local electronic pulse coupling. Sci. Robot. 9, eadn8067 (2024). 10.1126/scirobotics.adn8067 39602516

[71] Y. Deng, A. Paskert, Z. Zhang, R. Wittkowski, D. Ahmed, An acoustically controlled helical microrobot. Sci. Adv. 9, eadh5260 (2023). 10.1126/sciadv.adh5260 PMC10511192 37729400

[72] Z. Liang, M. X. Lim, Q.-Z. Zhu, F. Mottes, J. Z. Kim, L. Guttieres, C. Smart, T. Pearson, C. X. Du, M. Brenner, P. McEuen, I. Cohen, Magnetic decoupling as a proofreading strategy for high-yield, time-efficient microscale self-assembly. Proc. Natl. Acad. Sci. U.S.A. 122, e2502361122 (2025). 10.1073/pnas.2502361122 PMC12415251 40875809

[73] Q. Liu, W. Wang, H. Sinhmar, I. Griniasty, J. Z. Kim, J. T. Pelster, P. Chaudhari, M. F. Reynolds, M. C. Cao, D. A. Muller, A. B. Apsel, N. L. Abbott, H. Kress-Gazit, P. L. McEuen, I. Cohen, Electronically configurable microscopic metasheet robots. Nat. Mater. 24, 109–115 (2025). 10.1038/s41563-024-02007-7 39261721

[74] W. S. Harley, K. Kolesnik, M. Xu, D. E. Heath, D. J. Collins, 3D acoustofluidics via sub-wavelength micro-resonators. Adv. Funct. Mater. 33, 2211422 (2023).

[75] C. Chen, Y. Gu, J. Rufo, J. Zhang, K. Yang, Y. Chen, L. P. Lee, T. J. Huang, Acoustofluidic spin control for 3D particle manipulation in droplets. Sci. Adv. 11, eadx0269 (2025). 10.1126/sciadv.adx0269 PMC12083541 40378226

[76] A. A. Doinikov, Acoustic radiation force on a bubble: Viscous and thermal effects. J. Acoust. Soc. Am. 103, 143–147 (1998).

[77] C. P. Lee, T. G. Wang, Acoustic radiation force on a bubble. J. Acoust. Soc. Am. 93, 1637–1640 (1993).

[78] O. Louisnard, A simple model of ultrasound propagation in a cavitating liquid. Part II: Primary Bjerknes force and bubble structures. Ultrason. Sonochem. 19, 66–76 (2012). 10.1016/j.ultsonch.2011.06.008 21764349

[79] L. A. Crum, Bjerknes forces on bubbles in a stationary sound field. J. Acoust. Soc. Am. 57, 1363–1370 (1975).

[80] H. Chen, Z. Chen, Y. Li, Modulation of the secondary Bjerknes force in multi-bubble systems. Ultrason. Sonochem. 61, 104814 (2020). 10.1016/j.ultsonch.2019.104814 31675659

[81] T. Barbat, N. Ashgriz, C.-S. Liu, Dynamics of two interacting bubbles in an acoustic field. J. Fluid Mech. 389, 137–168 (1999).

[82] P. Tho, R. Manasseh, A. Ooi, Cavitation microstreaming patterns in single and multiple bubble systems. J. Fluid Mech. *576*, 191–233 (2007).

[83] M. S. Plesset, A. Prosperetti, Bubble dynamics and cavitation. Annu. Rev. Fluid Mech. 9, 145–185 (1977).

[84] A. Prosperetti, Bubble phenomena in sound fields: Part one. Ultrasonics 22, 69–77 (1984).

[85] A. A. Doinikov, S. T. Zavtrak, On the mutual interaction of two gas bubbles in a sound field. Phys. Fluids 7, 1923–1930 (1995).

[86] M. Ida, Phase properties and interaction force of acoustically interacting bubbles: A complementary study of the transition frequency. Phys. Fluids 17, 097107 (2005).

[87] C. Wang, J. Cheng, Cavitation microstreaming generated by a bubble pair in an ultrasound field. J. Acoust. Soc. Am. 134, 1675–1682 (2013). 10.1121/1.4812896 23927208

[88] A. A. Doinikov, A. Bouakaz, Microstreaming generated by two acoustically induced gas bubbles. J. Fluid Mech. 796, 318–339 (2016).

[89] S. M. van der Meer, B. Dollet, M. M. Voormolen, C. T. Chin, A. Bouakaz, N. de Jong, M. Versluis, D. Lohse, Microbubble spectroscopy of ultrasound contrast agents. J. Acoust. Soc. Am. 121, 648–656 (2007). 10.1121/1.2390673 17297818

[90] A. A. Doinikov, G. Regnault, C. Mauger, P. Blanc-Benon, C. Inserra, Acoustic microstreaming produced by two interacting gas bubbles undergoing axisymmetric shape oscillations. J. Fluid Mech. 931, A19 (2022).

[91] Y. Ma, G. Zhang, T. Ma, Interaction of two bubbles with distortion in an acoustic field. Ultrason. Sonochem. 84, 105953 (2022). 10.1016/j.ultsonch.2022.105953 PMC8861464 35184007

[92] M. Settnes, H. Bruus, Forces acting on a small particle in an acoustical field in a viscous fluid. Phys. Rev. E 85, 016327 (2012). 10.1103/PhysRevE.85.016327 22400677

[93] A. A. Doinikov, Acoustic radiation force on a spherical particle in a viscous heat-conducting fluid. I. General formula. J. Acoust. Soc. Am. 101, 713–721 (1997).

[94] K. Yosioka, Y. Kawasima, Acoustic radiation pressure on a compressible sphere. Acustica 5, 167–173 (1955).

[95] P. Marmottant, M. Versluis, N. de Jong, S. Hilgenfeldt, D. Lohse, High-speed imaging of an ultrasound-driven bubble in contact with a wall: “Narcissus” effect and resolved acoustic streaming. Exp. Fluids 41, 147–153 (2006).

[96] A. A. Doinikov, S. Zhao, P. A. Dayton, Modeling of the acoustic response from contrast agent microbubbles near a rigid wall. Ultrasonics 49, 195–201 (2009). 10.1016/j.ultras.2008.07.017 PMC2807700 18789469

[97] A. O. Maksimov, Radiation force on a bubble located near an interface. J. Acoust. Soc. Am. 151, 1464–1475 (2022). 10.1121/10.0009673 35364929

[98] S. A. Suslov, A. Ooi, R. Manasseh, Nonlinear dynamic behavior of microscopic bubbles near a rigid wall. Phys. Rev. E 85, 066309 (2012). 10.1103/PhysRevE.85.066309 23005208

