# Supplementary material for: Ultrasound-driven electronically programmable microbubbles
Source: Sci Adv. 2026 Oct 14;12(42):eaeg5408. doi: 10.1126/sciadv.aeg5408 (PMC13665997; doi:10.1126/sciadv.aeg5408)
Supplement: Supplementary file 1 [file sciadv.aeg5408_sm.pdf]

Science Advances
AAAS

Supplementary Materials for

Ultrasound-driven electronically programmable microbubbles

Zhiyuan Zhang *et al.*

Corresponding author: Zhiyuan Zhang, zz888@cornell.edu; Itai Cohen, itai.cohen@cornell.edu

Sci. Adv. **12**, eaeg5408 (2026)
DOI: 10.1126/sciadv.aeg5408

The PDF file includes:

Notes S1 to S4
Figs. S1 to S31
Legends for movies S1 to S12
Legends for COMSOL Output Reports S1 to S3
References

Other Supplementary Material for this manuscript includes the following:

Movies S1 to S12

Supplementary Note S1: Theoretical understanding and numerical simulations of flow polarization and reversal

For the flow polarization and flow reversal observed in **Figs. 3**, briefly, we propose that the flow polarization arises from a peristaltic mechanism induced by the phase difference between the oscillating bubbles. When one bubble leads the other during pulsation, constructive superposition of the flow field occurs on one side of the bubble pair or group, while partial destructive interference occurs on the other, and vice versa. We found that the oscillation phase of each bubble is determined by both the driving external acoustic field and the secondary acoustic interactions between the bubbles. For example, when the bubbles are nearly the same size, we observe symmetric flow fields that result from a 180° phase shift between the bubbles. The flow reversal observed when one bubble continuously grows can be attributed to changes in the relative oscillation phase and the swapping of the leading/lagging relationship between the two bubbles. In the following, we provide a detailed description of the modeling and simulations that we used to arrive at this understanding.

Starting from the oscillation of a single bubble, under small-amplitude oscillations, an ultrasonically driven bubble can be approximated as a damped harmonic oscillator (*81, 82*), with the instantaneous bubble radius written as

$$R(t) = R_0 + r(t), \tag{N1}$$

where R_0 is the equilibrium bubble radius, $r(t)$ is the small radial perturbation induced by ultrasound excitation, and t denotes time. $r(t)$ is the response of a forced damped oscillator.

We take the acoustic pressure as

$$p_{\mathrm{ac}} = p_0 \cos(\omega t), \tag{N2}$$

where p_0 is the acoustic pressure amplitude and ω is the angular frequency of the external drive. The linearized radial dynamics can be written as

$$\ddot{r} + 2\beta\dot{r} + \omega_0^2 r = -F\cos(\omega t), \tag{N3}$$

where ω_0 is the natural angular frequency of the bubble, β is the damping coefficient, and F is the effective forcing term associated with the external acoustic field. The negative sign reflects the fact that positive acoustic pressure compresses the bubble, whereas negative acoustic pressure drives

bubble expansion. Thus, the steady-state response of this system can be expressed as

$$r(t) = X\cos(\omega t + \phi), \tag{N4}$$

where X is the steady-state oscillation amplitude of the bubble radius and ϕ is the phase difference of the radial oscillation relative to the external acoustic pressure, which is

$$\phi = \arctan\frac{2\beta\omega}{\omega^2 - \omega_0^2}, \tag{N5}$$

where ω_0 is the resonant angular frequency of a bubble, which is governed by its equilibrium size. Therefore, at a fixed driving frequency, different-sized bubbles exhibit different degrees of detuning from the external drive (**Supplementary Fig. S17**). For bubbles smaller than the resonance-matched size, $\omega < \omega_0$, and the phase difference lies in the range $90° < \phi < 180°$. When the bubble size matches the resonant size, $\omega \approx \omega_0$, and the phase difference is approximately 90°. For bubbles larger than the resonance-matched size, $\omega > \omega_0$, and the phase difference approaches $0° < \varphi < 90°$ (*83, 84*).

In a two-bubble system, their local microstreaming fields will overlap and superpose when positioned within a small center-to-center distance. Because each bubble is not only driven by the externally applied acoustic field but also coupled to the other bubble through the secondary acoustic field generated by mutual scattering (*85–87*). This interaction can be described as a coupled complex-amplitude system

$$\begin{aligned} \left(\omega_{01}^2 - \omega^2 + 2i\beta_1\omega\right) X_1 + C_{12}(\omega, D)X_2 &= F_1, \\ C_{21}(\omega, D)X_1 + \left(\omega_{02}^2 - \omega^2 + 2i\beta_2\omega\right) X_2 &= F_2, \end{aligned} \tag{N6}$$

where F_1 and F_2 are the complex amplitudes of the external acoustic forcing evaluated at the positions of bubbles 1 and 2, respectively. X_1 and X_2 denote the complex oscillation amplitudes of the two bubbles. D is the inter-bubble distance. C_{12} and C_{21} are the coupling terms mediated by the scattered acoustic fields, depending on the oscillation amplitudes $|X_1|$, $|X_2|$, and phase difference of the two bubbles $\Delta\varphi$, as well as on their sizes, inter-bubble distance, the oscillation modes of the bubbles, and boundary conditions (*65, 88, 89*). Previous theoretical and experimental studies have shown that interactions between two or multiple bubbles can substantially modify the strength, direction, and topology of microstreaming flows (*90, 91*).

In our platform, the positions of the two bubbles remain fixed while their sizes are electronically modulated, and the bubbles are primarily excited in a volumetric oscillation mode. Consequently, we focus specifically on the physical possibility that the growth of a neighboring bubble changes the relative oscillation amplitude and phase difference between the two bubbles, thereby reorganizing the coupled streaming field and producing flow polarization.

To identify the contributions of the bubble-size ratio, oscillation-amplitude ratio, and phase difference, we constructed a simplified two-dimensional acoustic-streaming simulation model in COMSOL Multiphysics and systematically performed parametric sweeps of the bubble-size ratio, oscillation-amplitude ratio, and phase difference. In this model, two bubbles were placed at the center of the water domain with an inter-bubble distance of 100 μm, and the ultrasound excitation frequency was kept at 90 kHz, consistent with the experimental excitation. Three bubble-size combinations were considered: 27.5 and 55 μm, 55 and 55 μm, and 110 and 55 μm in diameter, as shown in **Supplementary Figs. S18, S19, and S20**, respectively. For each size combination, the oscillation amplitude of the right bubble (A_2) was held constant, while the oscillation amplitude of the left bubble (A_1) was varied to obtain oscillation-amplitude ratios (A_1/A_2) of 0.4, 0.6, 0.8, 1, 2, and 3. In parallel, the phase difference ($\Delta\varphi = \phi_1 - \phi_2$, $\phi_2 = 90°$) was prescribed as 0°, 90°, 180°, 270°, and 360°. The second-order steady streaming field was then obtained through a thermoviscous acoustics frequency-domain analysis followed by a stationary laminar-flow study (see **COMSOL Output Report S1** for detailed simulation configurations).

The parametric sweeps revealed that, under the present two-bubble ultrasound excitation configuration, the bubble-size ratio and oscillation-amplitude ratio primarily modulate the relative streaming strength, whereas the phase difference plays the dominant role in transitioning the streaming topologies. To examine the streaming state associated with each phase condition, we further prescribed the phase difference between the two bubbles from 0° to 360° in 10° increments while keeping their sizes and oscillation amplitudes constant.

To quantify the directionality of the streaming field, we defined a polarization index, P, as the mean horizontal velocity along the centerline between the two bubbles, with its sign and magnitude characterizing the direction and degree of flow polarization, respectively. Based on the simulated streaming patterns and the corresponding values of P, the phase-difference sweep revealed four distinct flow topological regimes (**Supplementary Fig. S21**). Near $\Delta\varphi \approx 0°$, the two bubbles

oscillate approximately in phase. Thus, the time-averaged streaming field is left–right symmetric with the upper-right streaming vortex rotating clockwise and $P \approx 0$. As the phase difference increases, $0^\circ \leq \Delta\varphi < 50^\circ$ and $P > 0$; however, the time-averaged streaming field remains four vortices. Therefore, we consider these streaming states to belong to the in-phase symmetric regime. For $50^\circ < \Delta\varphi < 150^\circ$, the streaming field becomes left-polarized with two vortices, with P remaining positive and reaching its maximum at $\Delta\varphi = 100^\circ$. Near $\Delta\varphi \approx 180^\circ$, the two bubbles oscillate approximately out of phase. The streaming field again remains left–right symmetric with four vortices. But its rotational chirality is reversed, with the upper-right streaming vortex rotating counterclockwise. Similarly, we regard these four-vortex streaming states with $150^\circ < \Delta\varphi < 210^\circ$ as belonging to the anti-phase symmetric regime. For $210^\circ < \Delta\varphi < 320^\circ$, the streaming field is reversed and becomes right-polarized with two vortices. In this regime, P remains smaller than 0, with its minimum at $\Delta\varphi = 260^\circ$. Subsequently, for $320^\circ < \Delta\varphi < 360^\circ$, these four-vortex streaming states shift back to the in-phase symmetric regime. The results demonstrate that continuously changing the phase difference between two bubbles can induce flow polarization, symmetric flow, and flow reversal in sequence with $P \sim \sin\Delta\varphi$, while also determining the chirality of the symmetric streaming states.

It should be noted that, in this simplified model, the transition boundaries between these regimes are not universal. In a different system, they are governed by a combination of factors, including bubble geometry, excitation conditions, amplitude asymmetry, finite-domain effects, and the degree of nonlinear rectification. Therefore, the simulations are used to elucidate the relationship between phase difference and flow polarization, rather than to claim an exact point-by-point reproduction of the experiments.

According to the simulations, across the full phase difference space, there are two points corresponding to $P = 0$, which are $\Delta\varphi = 0^\circ$ and $\Delta\varphi = 180^\circ$, representing two potential phase evaluation branches that cause flow polarization and flow reversal. However, based on the damped harmonic oscillator, for a linear resonant bubble driven by an external acoustic field, when it is driven successively above resonance, near resonance, and below resonance, its oscillation phase relative to the external sound field can only vary continuously from 180° to 0°, or equivalently, from 180° to 360°, depending on the excitation conditions, instead of mapping the full mode.

In experiments, as the left bubble grows while the right bubble remains fixed, we observed that

the system traces only a subset of the full phase map, passing from the left-polarized regime to the near anti-phase symmetric regime and then to the right-polarized regime, without passing through the in-phase symmetric regime. Therefore, combined with the simulation and damped harmonic oscillator theory, it is assumed that since the right bubble is near the resonant size, with $\phi_r \approx 90°$, and as the left bubble radius is increased from zero, ϕ_l = 180°–360°, resulting in the phase difference between the two bubbles increases from 90° to 270°. When the phase difference passes through 180°, the oscillating leadership between the two bubbles is flipped, resulting in the flow reversal. We therefore identify $\Delta\varphi$ = 90°–270° as a typical experimentally observed phase-difference range.

In summary, for the coupled-bubble system shown in **Fig. 3**, we found that the flow polarization arises from a peristaltic mechanism induced by the phase difference between the oscillating bubbles. When one bubble leads the other during pulsation, constructive superposition of the flow field occurs on one side of the bubble pair while partial destructive interference occurs on the other, and vice versa. Since the variation in oscillation phase stems from whether the bubble is above or below resonance and the flows induced by the second bubble, the flow reversal observed as one bubble continuously grows can be attributed to changes in the relative oscillation phase and the swapping of the leading/lagging relationship between the two bubbles. We established the relationship between bubble size and oscillation phase using a damped harmonic-oscillator model. We then performed parametric simulations by independently varying the bubble-size ratio, oscillation-amplitude ratio, and phase difference to identify their respective roles. The parametric sweeps revealed that, under the present two-bubble ultrasound excitation configuration, the bubble-size ratio and oscillation-amplitude ratio primarily modulate the relative streaming strength, whereas the phase difference plays the dominant role in transitioning the streaming topologies. The phase sweep further revealed four distinct streaming topologies in the full phase map. While a linear resonant bubble driven by an external acoustic field can only vary in phase continuously from 180° to 0°, the driving due to the second bubble extends the range of possible phases. In experiments, we observed that the system passes from the left-polarized regime to the near anti-phase symmetric regime and then to the right-polarized regime, without passing through the in-phase symmetric regime. The observed flows are consistent with a scenario where the right bubble is near resonance with a phase of 90° relative to the driving acoustic field and the left bubble exhibits a phase-difference range $\Delta\varphi$ = 90°–270° as its size is varied.

Supplementary Note S2: Quantification of the bubble-size ratio for flow polarization in bubble pairs

In **Fig. 3C**, flow polarization was observed when the satellite-to-central bubble-size ratio was approximately 0.4–0.6. This intermediate size ratio reflects a balance between two competing requirements: the satellite bubble must oscillate strongly enough to contribute appreciably to the coupled flow field, while remaining sufficiently different in size from the central bubble to preserve an amplitude–phase asymmetry. In the experiments, both the oscillation amplitudes and the phase difference of the two bubbles are governed by their respective sizes. When the satellite bubble is too small, its oscillation amplitude and corresponding contribution to the flow field are too weak to substantially modify the overall streaming pattern (**Movie S4**). By contrast, as the satellite bubble approaches the size of the central bubble, its oscillation amplitude becomes substantial, but the phase difference between the two bubbles approaches 180°, producing a symmetric four-vortex streaming pattern (**Fig. 3Ac**). Therefore, flow polarization emerges only within an intermediate size-ratio range, in which the satellite bubble contributes sufficiently to the coupled flow field while a pronounced amplitude–phase imbalance is retained.

To quantitatively assess this effect, we evaluated the polarized-flow speed as a function of the satellite-to-central bubble-size ratio. We first extracted the bubble-size ratio, $\chi = D_s/D_c$, from the measured diameters of the satellite and central bubbles. Then, we quantified the polarized-flow speed using the average optical-flow speed within a selected region of interest (ROI) aligned with the experimentally observed polarization direction (**Supplementary Fig. S22A**). For each polarization direction, the ROI was placed consistently in the corresponding downstream region of the flow. When no directional polarization could be resolved, the polarized-flow speed was assigned a value of zero.

Supplementary Fig. S22B shows that, for the bubble-pair configuration shown in **Fig. 3B**, the polarized-flow speed is maximal in an intermediate size-ratio regime of approximately 0.4–0.6 and decreases to zero as the satellite bubble becomes too small. In this configuration, data above $\chi \approx 0.6$ could not be robustly obtained because the 50 μm electrode spacing constrained the accessible bubble separation, and the two bubbles tended to merge once the satellite bubble became too large. Therefore, in this configuration, the experimentally accessible regime of strongest polarization is approximately 0.4–0.6.

It is worth noting that this range of bubble-size ratios does not represent a universal constant applicable to all bubble pairs. Instead, the occurrence of strong flow polarization depends sensitively on conditions such as bubble size, inter-bubble distance, bubble oscillation modes, and the local acoustic field. To further illustrate this point, we conducted a supplementary analysis of the bubble pair shown in **Fig. 3A**, which has a larger inter-bubble distance of 100 μm. In this case, the range of size ratios corresponding to the polarized flow regime is broader, spanning approximately 0.4–0.9 (**Supplementary Fig. S23**).

In summary, these analyses support the physical picture that the strongest polarization emerges when the two bubbles are strongly coupled while still maintaining sufficient asymmetry in their oscillation responses. The exact size-ratio range is not a universal constant; instead, it depends sensitively on conditions such as bubble size, inter-bubble distance, bubble oscillation modes, and the local acoustic field.

Supplementary Note S3: A coupled field–resonance mechanism underlying frequency-selective bubble shooting

For the bubble shooting shown in **Fig. 5**, we found that a coupled field-resonance mechanism explains the behavior. Briefly, the direction of bubble shooting is governed jointly by the spatial acoustic field distribution and the bubble resonant condition, rather than by either term alone. The direction of the total acoustic radiation force acting on the detaching bubble is primarily determined by two components of the primary Bjerknes force: the pressure-gradient component, which depends mainly on the acoustic pressure distribution within the acoustic chamber; and the phase-gradient component, which depends largely on how closely the bubble's state approaches resonant conditions. Varying the excitation frequency simultaneously affects the contributions of these two forces: changes in frequency alter the acoustic field within the cavity via substrate vibration modes, thereby primarily influencing the pressure-gradient component; meanwhile, frequency changes affect the bubble's response characteristics by inducing resonant detuning and altering the bubble's detachment size, thereby primarily influencing the phase-gradient component.

Below, we present five analyses to support this understanding: (**i**) we performed experiments with different electrode geometries to distinguish acoustically driven effects from electrode-specific pinning and electrolysis effects; (**ii**) we proposed a force analysis framework for the detached bubble to elucidate its dynamics; (**iii**) we simulated the acoustic pressure distribution in the acoustic chamber to reveal its frequency-sensitive character; (**iv**) we simulated radiation forces to isolate the respective contributions of the pressure-gradient component and phase-gradient component of the primary Bjerknes force; (**v**) Finally, we assessed the role of secondary Bjerknes forces based on experimental observations of bubbles remaining close to the substrate during shooting and the convergence of neighboring shooting trajectories.

(**i**) First, to test whether the observed directional shooting is determined by electrode geometry, contact-line pinning, or electrolysis dynamics, we fabricated microelectrodes with varying exposed dimensions and geometries, thereby generating different bubble detachment conditions and a range of detachment sizes (**Supplementary Fig. S24A**). In the absence of ultrasound, bubbles generated from all electrodes rose to the liquid-air interface without exhibiting near-wall directional shooting (**Supplementary Fig. S24B**). Under 97 kHz ultrasound excitation, bubbles released from different electrodes moved toward the bottom-right (**Supplementary Fig. S24C**). When the frequency was

shifted to 125 kHz, the bubbles primarily shot toward the left (**Supplementary Fig. S24D**). Thus, at a fixed frequency, the direction of bubble shooting remained consistent across different electrodes and underwent a collective shift in response to changes in frequency. Consequently, these experiments demonstrate that while electrode geometry, contact-line pinning, and the electrolysis process dictate the size and timing of bubble detachment, they do not determine the primary shooting direction following detachment.

(**ii**) To elucidate the dynamics of a detached bubble, we combined several standard force analysis methods into a unified framework, as illustrated in **Supplementary Fig. S25**. An ultrasound-excited bubble situated in a liquid near a solid substrate is subjected to hydrodynamic and acoustic forces. The hydrodynamic terms include buoyancy (orange arrow), gravity (gray arrow), and drag (brown arrow). The acoustic contribution is the total time-averaged radiation force primarily comprising the primary Bjerknes force (dark green arrow), the residual momentum-flux correction (blue arrow), the wall-mediated secondary Bjerknes force (teal arrow), and the neighbor-bubble-mediated secondary Bjerknes force (black arrow).

Total acoustic radiation force: The total radiation force acting on a bubble can be evaluated by integrating the time-averaged total acoustic momentum flux over a closed control surface S that encloses only the bubble:

$$\mathbf{F}_{\mathrm{ac}} = -\oint_S \langle \mathbf{\Pi}_{\mathrm{ac}} \rangle \cdot \mathbf{n}\, \mathrm{d}S, \tag{N7}$$

where $\mathbf{n}$ is the outward unit normal vector to S. $\mathbf{\Pi}_{\mathrm{ac}}$ is the acoustic momentum-flux tensor constructed from the total first-order acoustic field. The negative sign indicates that the force acting on the enclosed bubble equals the negative of the net outward acoustic momentum flux. The formulation provides a general numerical approach for determining the total acoustic radiation force and accounts for the combined contributions of the incident field, bubble scattering, and interference effects (*92, 93*).

Primary Bjerknes force: For a subwavelength bubble whose response is dominated by spherical volumetric oscillations, the leading-order translational force exerted by an external acoustic field on the bubble can be approximated by the primary Bjerknes force (*67*), expressed schematically as:

$$\mathbf{F}_B^{\mathrm{pri}} = -\langle V(t) \nabla p\, (\mathbf{x}_b, t) \rangle = -\frac{1}{2}\, \mathrm{Re}\left[\widehat{V}\, (\nabla \widehat{p}_0)^* \right], \tag{N8}$$

where $V(t)$ is the instantaneous bubble volume, ∇p is the acoustic pressure gradient, $\mathbf{x}_b$ is the

bubble position. $\langle\cdot\rangle$ denotes the time averaging over one acoustic cycle. p_0 is the external acoustic pressure evaluated at the bubble position and excluding the target bubble's self-field, a hat denotes a complex harmonic amplitude, and an asterisk denotes complex conjugation. In this framework, bubble resonance determines the phase and amplitude of the bubble-volume oscillation $V(t)$, whereas the acoustic field in the chamber determines the local pressure-gradient field $\nabla p(\mathbf{x}_b, t)$. Therefore, the primary Bjerknes force is governed by both the bubble resonant conditions and the spatial distribution of the acoustic field, rather than by either term alone.

By writing $\widehat{p}_0 = Ae^{i\phi}$, and $\Delta\phi = \phi_V - \phi$, the complex form of $\mathbf{F}_B^{\text{pri}}$ can be decomposed into a pressure-gradient contribution and a phase-gradient contribution (*76, 77*):

$$\mathbf{F}_B^{\text{pri}} = -\frac{\left|\widehat{V}\right|}{2}\cos(\Delta\phi)\nabla A - \frac{\left|\widehat{V}\right|}{2}\sin(\Delta\phi)A\nabla\phi. \tag{N9}$$

Residual momentum-flux correction: This correction accounts for scattering/interference, finite-size, near-wall, and higher-order contributions that are not captured by the leading-order primary Bjerknes approximation. It is defined as the difference between the total acoustic radiation force obtained from the time-averaged momentum-flux integral and the two leading-order primary Bjerknes components.

Secondary Bjerknes force: The force arises from the scattered acoustic field generated by a neighboring bubble or by the wall-mediated image bubble and can be expressed schematically as

$$\mathbf{F}_B^{\text{sec}} = -\langle V_1(t)\nabla p_2(\mathbf{x}_1, t)\rangle \approx \frac{\rho_0}{4\pi d^2}\left\langle \dot{V}_1(t)\dot{V}_2(t)\right\rangle \mathbf{e}_{12}, \tag{N10}$$

where $p_2(\mathbf{x}_1, t)$ is the scattered acoustic pressure generated by bubble 2 or by the image bubble and evaluated at the position of bubble 1. d is the center-to-center distance between the two bubbles, $\mathbf{e}_{12}$ is the unit vector pointing from bubble 1 to bubble 2, and a positive value of $\left\langle \dot{V}_1\dot{V}_2\right\rangle$ corresponds to attraction (*78–80*).

(iii) To illustrate the respective contributions of each radiation force component, we started from the intuition for the first term of the primary Bjerknes force associated with the pressure gradient. We constructed a simulation model to analyze the substrate vibration modes and acoustic pressure distribution within the chamber using geometric structures and material properties based on those used in the experiments. Representative ultrasound excitation frequencies of 74, 81, 84, 88, 90, and 94 kHz were simulated (**Supplementary Fig. S13**). Detailed simulation configurations are provided in **COMSOL Output Report S2**.

Simulations revealed that both the global vibration modes of the substrate and the acoustic pressure distribution inside the chamber exhibit pronounced frequency sensitivity. The pressure distribution is spatially nonuniform, with particularly strong variations near the liquid–air and liquid–PDMS boundaries, whereas the pressure magnitude is comparatively homogeneous near the liquid-substrate interface and in portions of the bulk liquid. Notably, in experiments, the acoustic field could deviate from the idealized simulation because the transducer and chamber are assembled manually. Small variations in transducer position, bonding-layer thickness, chamber alignment, and interfacial boundary conditions can modify the substrate vibration modes and the resulting pressure distribution. The simulations are used to identify the frequency-sensitive character of the chamber acoustic field and the associated force mechanism, rather than to claim an exact point-by-point reproduction of the experimental pressure distribution.

(iv) Because the simulation suggests that the pressure distribution in the experiments is likely complex, below we analyze the remaining force terms in the context of a simpler traveling wave field, which is written as

$$P_b(x, y) = p_0 \left[1 + \alpha \left(x \cos\theta_g + y \sin\theta_g\right)\right] e^{-ik_0(x\cos\theta_k + y\sin\theta_k)}, \qquad \text{(N11)}$$

where, $P_b(x, y)$ is the prescribed background-pressure complex amplitude, p_0 is the reference pressure amplitude, x, y are the spatial coordinates, α is the signed pressure-magnitude-gradient coefficient, θ_g defines the gradient axis, θ_k defines the propagation direction, and k_0 is the acoustic wavenumber (*94*).

To aid visualization of the different radiation force components, we simulated a wave propagating along $+y$ with the pressure-gradient axis inclined at 45°, by setting $\alpha = -300\ \mathrm{m}^{-1}$, $\theta_k = 90°$, and $\theta_g = 45°$ (**Supplementary Fig. S26A**). The driving frequency was set to the resonant frequency of a 30 μm diameter bubble, while the bubble size and position were varied for characterization. The model does not include the solid substrate or neighboring bubbles. Detailed simulation configurations are provided in **COMSOL Output Report S3**. Next, we calculated the contributions from both the pressure-gradient component (magenta arrow) and the phase-gradient component (green arrow). For completeness, we also plotted the contribution arising from the residual momentum-flux correction (blue arrow). In this simplified model, this residual correction was approximated using the pure scattered-field momentum-flux contribution as $\mathbf{F}_{\mathrm{scat}} = -\oint_S \langle \mathbf{\Pi}_{\mathrm{scat}} \rangle \cdot \mathbf{n}\, \mathrm{d}S$. The vector

sum of all three contributions gives the approximate resultant radiation force (red arrow).

While the bubble position was fixed at the center of the domain and the bubble diameter varied from 10 μm to 60 μm, simulations revealed three characteristic force regimes (**Supplementary Fig. S26B**). For bubbles smaller than the resonant diameter, the pressure-gradient component points along $+\nabla p$. This is because the driving frequency is smaller than the bubble resonant frequency, $\omega < \omega_0$, with $90^\circ < \Delta\phi < 180^\circ$ and $\cos(\Delta\phi) < 0$. For bubbles larger than the resonant diameter, the pressure-gradient component reverses and points along $-\nabla p$. Similarly, because the driving frequency is larger than the bubble resonant frequency, $\omega > \omega_0$, with the phase difference ranging $0^\circ < \Delta\phi < 90^\circ$ and $\cos(\Delta\phi) > 0$. Furthermore, because the bubble is away from resonance, the phase-gradient contribution is weak, allowing the pressure-gradient term to dominate the primary Bjerknes force.

Conversely, at resonance, the pressure-gradient component vanishes, and the phase-gradient component becomes dominant. Because the volume oscillation of the resonant bubble is approximately in quadrature with the local background pressure, $\Delta\phi \approx 90^\circ$ and therefore $\cos\Delta\phi \approx 0$. This phase-gradient component is directed along the local wave propagation and momentum transport direction. It is governed by the phase gradient of the acoustic field rather than by the pressure gradient, although the bubble itself perturbs the local total acoustic field.

In all three force regimes, the residual momentum-flux correction is generally smaller than the dominant primary Bjerknes' contribution and does not substantially change the resultant force direction. An exception occurs for very small bubbles, such as a 10 μm diameter bubble. Since this bubble is far from resonance, both primary Bjerknes components are weak owing to its small volumetric response. As a result, the residual momentum-flux correction becomes the dominant contribution in the vector decomposition. However, for even smaller bubbles, the residual correction decreases as the scattered field and overall acoustic response further diminish.

Subsequently, we varied the bubble's position while keeping the bubble diameter and the established sound field constant. The results demonstrate that, across the entire sampling region, the direction of the force exerted on bubbles of the same size remains virtually identical (**Supplementary Fig. S26C** and **S26D**). This spatial robustness explains the similar initial shooting directions observed for bubbles detached from adjacent electrodes (**Fig. 5F**).

(v) Finally, we assessed the role of secondary Bjerknes forces based on experimental observa-

tions. In the absence of ultrasound, detached bubbles rise directly toward the liquid–air interface driven by buoyancy. However, under ultrasound excitation, bubbles are confined near the substrate and move laterally rather than simply rising vertically (as shown in the initial stage of **Movie S9**). We found that the confinement of bubbles near the substrate can be attributed to the action of the wall-mediated secondary Bjerknes force. This is because, under ultrasound excitation, a bubble near the substrate interacts with a virtual image bubble located on the opposite side of the wall. This image bubble oscillates approximately in phase with the real bubble, generating an attractive force directed toward the wall (*95–98*). This interaction contributes substantially to counteracting the upward buoyancy.

If a second bubble exists in the vicinity of the shooting bubble, the secondary Bjerknes force from the neighboring bubble further promotes attraction, aggregation, and coalescence between the two, as evidenced by the trajectory changes shown in the right panel of **Fig. 5E**. As this secondary interaction is sufficiently strong, the two bubbles coalesce into a larger new bubble; subsequently, the motion path of this new bubble changes significantly due to alterations in volume and resonant characteristics. The convergence of bubble trajectories from two rows, shown in the third panel of **Fig. 5F**, also corroborates this, as the emission directions from the two electrodes are similar, leading to a mutual attraction between the bubbles.

In summary, the experiments, force analyses, and simulations support a coupled field–resonance mechanism underlying frequency-selective bubble shooting. Before bubble detachment, electrochemical gas generation, capillary retention, contact-line pinning, buoyancy, and acoustic forcing jointly determine the bubble detaching timing and size. Following detachment, bubble motion is primarily governed by the total time-averaged acoustic force. The lateral motion of the bubble is primarily determined by the primary Bjerknes force, which can be decomposed into pressure-gradient and phase-gradient components. Thus, the primary Bjerknes force is governed jointly by the spatial acoustic field distribution and the bubble resonant condition, rather than by either term alone. Varying the excitation frequency alters two aspects of the force-coupling: it modifies the acoustic field via substrate vibration modes and influences bubble response behavior by changing the resonant detuning state and bubble detachment size. These coupled changes in wave propagation, pressure gradients, and bubble oscillation phase and amplitude determine the relative dominance of the pressure-gradient and phase-gradient force components, which can rotate or

reverse the lateral acoustic radiation force and thus produce frequency-selective bubble shooting. Additionally, the wall-mediated secondary Bjerknes force confines the bubble near the substrate by counteracting buoyancy. Finally, a secondary Bjerknes force arising from neighboring bubbles further facilitates mutual attraction, aggregation, and coalescence due to the change in volume and resonant characteristics. Hydrodynamic drag balances the resultant force during motion.

Supplementary Note S4: Stability, repeatability, and durability of the electrical bubble-customization platform

To quantitatively characterize the stability, repeatability, and robustness of the active bubble-customization platform, we measured bubble-size evolution after electrolysis was discontinued and conducted image-based closed-loop bubble-size control, bubble-configuration regeneration, and repeated write/erase cycling tests.

First, to evaluate stability, we tracked the diameters of generated bubbles over time without ultrasound excitation (**Supplementary Fig. S27**). Bubbles with diameters larger than 100 μm underwent a short shrinkage period of approximately 2 min and then maintained a relatively stable size for more than 1 h. Bubbles with diameters between 50 μm and 100 μm gradually shrank over approximately 200–500 s. Bubbles smaller than 50 μm shrank rapidly and disappeared within approximately 10–200 s. This size-dependent shrinkage can be primarily attributed to gas dissolution and diffusion driven by the pressure imbalance across the bubble interface, including the larger Laplace pressure associated with smaller bubbles. The abrupt jumps and fluctuations in some diameter traces are attributed to image-detection errors, stage vibration, and optical distortion caused by liquid evaporation during long-term observation. Notably, during ultrasound excitation, bubble oscillation may further modify the dissolution dynamics. In particular, the central bubble maintained a stable size under ultrasound excitation over the experimental timescale (**Fig. 3C**).

These measurements also indicate how long the programmed states in **Figs. 3** and **4** can remain usable before refresh or regeneration is needed. For the bubble-pair configuration shown in **Fig. 3B**, the smaller satellite bubble, with a diameter of approximately 20–30 μm, gradually shrank, and the corresponding polarized-flow state remained usable for approximately 5–8 s before refresh or regeneration was required. In contrast, for the L-shaped bubble array shown in **Fig. 4D**, where bubbles with diameters of approximately 30–50 μm were used, the programmed turning-flow state remained stable for approximately 30–150 s. When larger bubbles with diameters of approximately 50–100 μm were used, the bubbles maintained relatively stable sizes under the current ultrasound excitation conditions, and the corresponding flow state could persist for approximately 5–10 min. Moreover, this long-lived flow state could be repeatedly turned on and off by controlling the ultrasound excitation. These results demonstrate that larger-bubble configurations can support long-lived programmed flow states, whereas small-bubble configurations may require periodic

refresh due to faster gas dissolution. This size-governed lifetime further highlights the advantage of our platform: short-lived small-bubble states can be refreshed quickly when needed, whereas larger-bubble configurations can support longer-term programmable flow operation.

Importantly, the key feature of our platform is that the bubbles are not passive, one-time trapped cavities. Instead, their size and position can be actively maintained, adjusted, erased, or regenerated by localized electrolysis. To demonstrate this capability, we performed an additional experiment in which the shrunk bubble in the L-shaped array was actively refreshed by localized electrolysis. By increasing the size of the shrunk bubble back to the programmed range, the turning-flow state could be maintained and recovered on demand (**Supplementary Fig. S28**).

We also performed an image-based closed-loop control of bubble diameter. A Basler camera was used to acquire the diameter of the generated bubble, while an FPGA-based controller delivered voltage pulses to the selected microelectrode for localized electrolysis. At each control cycle, the measured bubble diameter D was compared with a prescribed target diameter D_{target}. When D was smaller than D_{target} by more than a predefined tolerance, an electrolysis pulse was applied to the selected electrode. The pulse duration was adjusted according to the diameter deficit. Specifically, no pulse was applied when the deficit was within the tolerance; a maximum pulse duration of 50 ms was applied when the deficit exceeded the full-pulse threshold; otherwise, the pulse duration was scaled linearly with the diameter deficit. After each electrolysis pulse, a new image was acquired, and the bubble diameter was re-evaluated. Up to three corrective pulses were allowed within each control cycle to reduce overshoot. In the experiments described here, the target diameter was set to 30 μm, the tolerance was 0.5 μm, the detection period was 1 s, and the total control duration was 5 min. **Supplementary Fig. S29** shows the diameter–time trace.

The current implementation used a simple event-triggered optical feedback scheme in which the electrolysis pulse duration was scaled according to the instantaneous diameter deficit. This approach provides a simple route for compensating bubble shrinkage and maintains the bubble diameter near a prescribed setpoint (30 μm) with the average error smaller than 5 μm. This experiment demonstrated robust dynamic bubble detection and modulation. Future work could further improve control accuracy by incorporating the temporal dynamics of bubble growth and dissolution. A calibrated growth model, PID controller, or model-predictive controller could be used to anticipate the diameter change induced by each pulse and reduce overshoot near the target size. In addition, integrating an

active shrinkage or erasure mechanism would enable bidirectional regulation, allowing the bubble diameter to be adjusted both upward and downward. These developments, together with improved real-time image segmentation, would provide smoother and more precise bubble-size control for long-term programmable acoustofluidic operation.

Second, we evaluated the repeatability of regenerated bubble configurations and the resulting flow patterns. We repeated the bubble-pair flow-polarization experiments (**Fig. 3B**) 40 times on a single device, with each polarization direction repeated 10 times. Each trial generated a similar polarized-flow pattern. We also repeated the L-shaped bubble-array experiment (**Fig. 4Dc**) 20 times on a single device, and each cycle produced a similar turning-flow pattern. These results demonstrate that the programmed bubble configurations and the associated flow topologies can be regenerated reproducibly.

Finally, in terms of durability, we performed repeated write/erase cycling tests while monitoring the electrolysis current with an electrochemical workstation. Two cyclic electrolysis protocols were designed to correspond to the two write/erase strategies. First, for the global erasure in which bubbles are removed by increasing the ultrasound excitation voltage to induce strong oscillation, we used a cyclic electrolysis recipe consisting of 1 s at −3.3 V followed by 1 s at 0 V. This recipe was repeated for 2000 cycles, and the current was sampled every 0.1 s. Three independent tests were performed on three different electrodes. The electrolysis current remained stable over repeated cycles, indicating good repeatability under this relatively mild electrolysis condition (**Supplementary Fig. S30**). The average measured currents for electrolysis at −3.3 V were −473.75 nA, −553.49 nA, and −393.04 nA, respectively. The current drift observed during cycling may arise from sampling fluctuations and transient bubble nucleation/detachment events during repeated electrolysis.

Then, for selective erasure through localized bubbling, we used a stronger cyclic electrolysis protocol consisting of 1 s at −3.3 V followed by 1 s at −5 V. This protocol was also repeated for 2000 cycles, with the current sampled every 0.1 s. Three independent tests were performed on three different electrodes. The electrolysis current at both −3.3 V and −5 V remained relatively stable during the first approximately 1000 cycles (**Supplementary Fig. S31**). After approximately 1500 cycles, the current at −5 V gradually decreased, and the current at −3.3 V also decreased to approximately −200 nA. This degradation is attributed to the stronger hydrogen evolution and localized bubbling at −5 V. Repeated bubble nucleation and detachment under high cathodic bias

may progressively alter the surface of the sputtered Pt thin-film electrode, for example, through local roughening, contamination, microcracking, or partial degradation of the Pt/adhesion-layer interface. When the current magnitude at −5 V fell below approximately 5 μA, no notable localized bubbling was observed, indicating failure of that electrode for selective bubble erasure. The long-term stability and duty cycle of the Ti/Pt electrodes could be improved by increasing the Pt thickness, optimizing electrode geometry, or adopting alternative electrode materials with enhanced electrochemical durability.

Consequently, the statement that "the platform addresses the challenge of limited bubble lifespan encountered by state-of-the-art microbubble systems" should be understood in an operational sense. We do not imply that every individual bubble remains indefinitely stable after a single generation step. Rather, the platform mitigates the practical lifetime limitation of passively trapped bubbles by allowing bubbles to be written, erased, regenerated, and reconfigured electronically at selected locations. For smaller bubbles that shrink over shorter timescales, this loss can be compensated through repeated electrolysis pulses or closed-loop optical feedback. The relevant operational lifetime is therefore no longer governed solely by the passive dissolution time of an individual bubble, but by the ability of the electrode array to reproducibly regenerate and maintain the programmed bubble configuration over an extended period. Furthermore, under test conditions, the write/erase scheme remained stable over thousands of cycles. This refreshable and repeatable operation provides a practical basis for our platform.

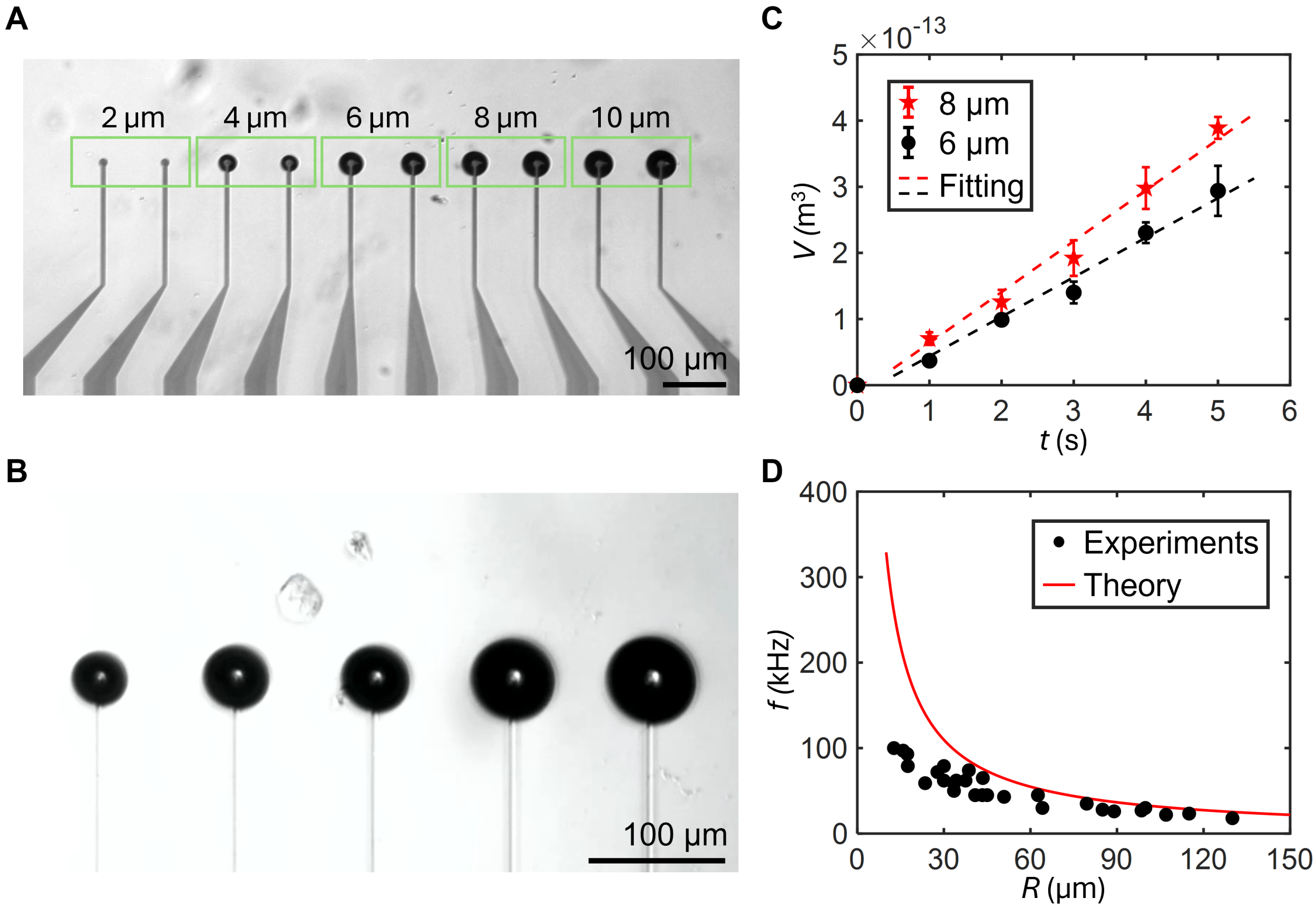

Figure S1: **Characterization of microbubble generation and the corresponding resonant frequency.** (**A**) Microbubbles generated with different exposed electrode areas under an electrolysis voltage of −3.3 V for 1 s. Green rectangles indicate the electrode diameters. (**B**) Microbubbles generated using 6 µm-diameter electrodes under electrolysis at −3.3 V for 1–5 s. (**C**) Microbubble volume as a function of electrolysis time. Dashed lines indicate linear fits. Error bars represent the mean ± s.d. (n = 6 independent measurements). (**D**) Microbubble resonant frequency as a function of microbubble radius.

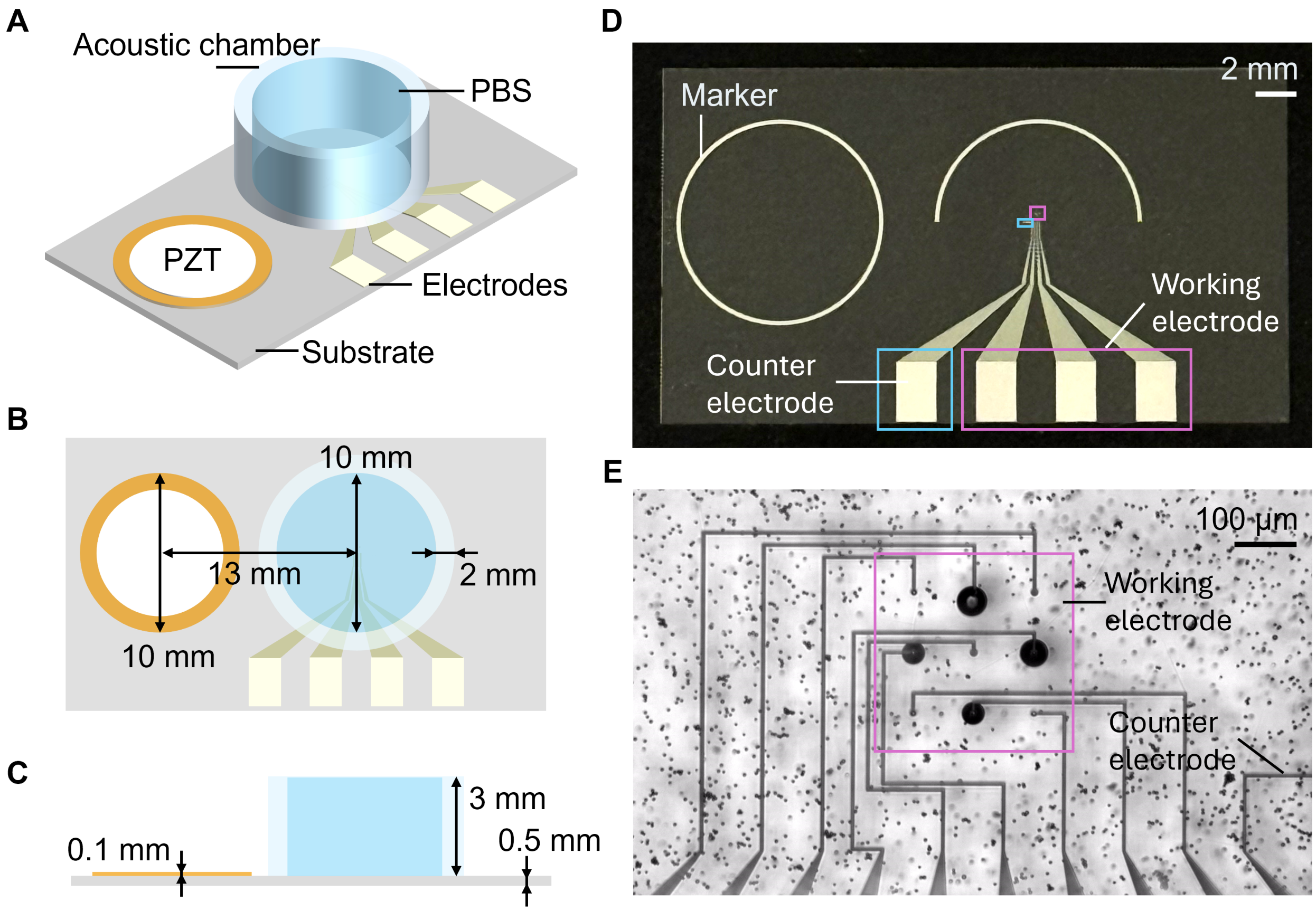

Figure S2: **Schematic and optical images of the experimental setup.** (**A**) 3D schematic, (**B**) top view, and (**C**) side view of the setup with key geometric dimensions illustrated. (**D**) Images of the substrate after microfabrication. (**E**) Micrograph showing an expanded view of a nine-electrode array with generated bubbles and tracer particles. The magenta box indicates the working electrodes, and the blue box indicates the counter electrode.

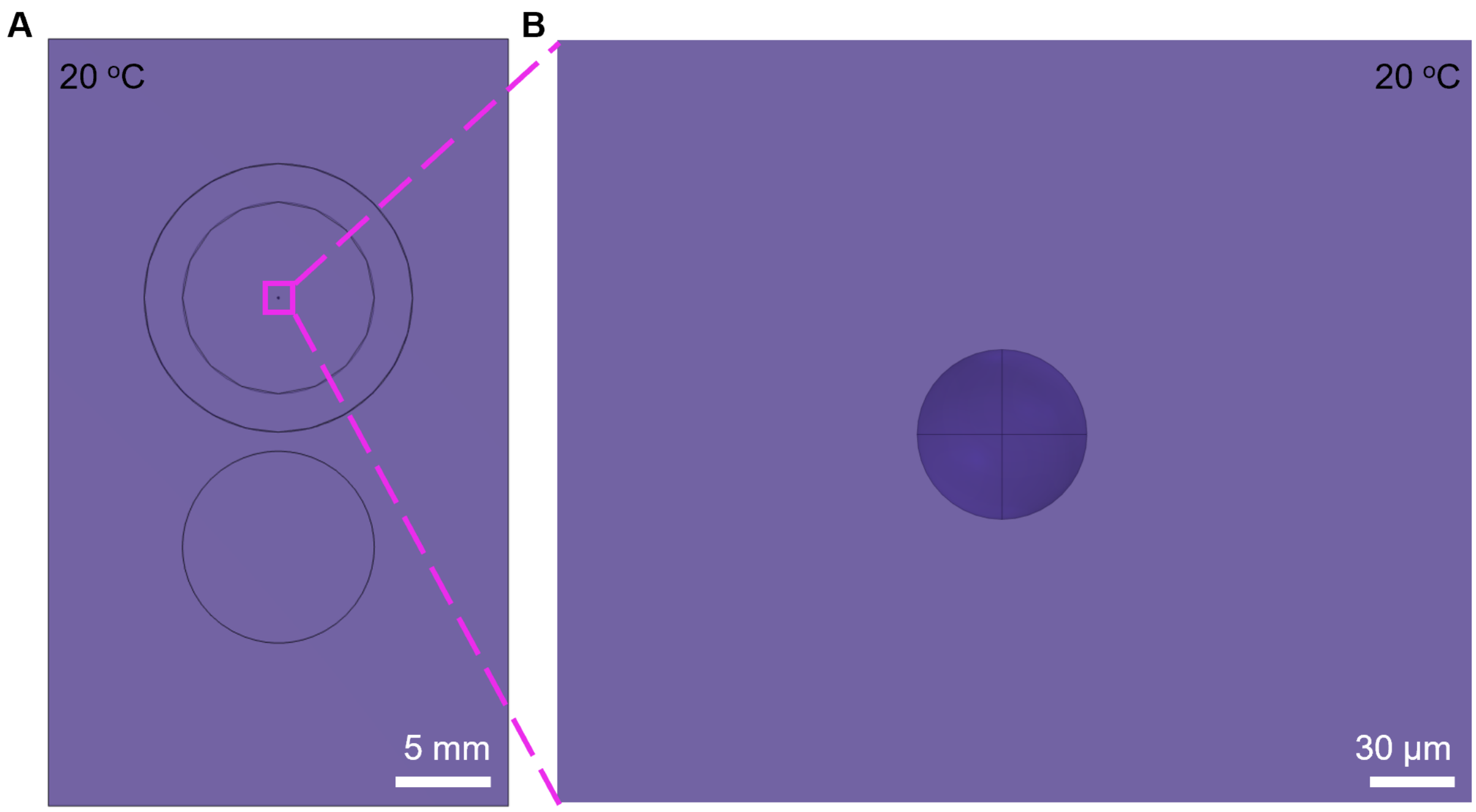

Figure S3: **Simulated temperature distribution.** (**A**) Temperature distribution within the acoustic chamber. (**B**) Local temperature distribution around the generated bubble over the experimentally relevant frequency range of 1–100 kHz under 20 V_{PP} ultrasound excitation, indicating no appreciable local temperature increase around the bubble under these operating conditions. The color denotes the ambient temperature of 20 °C

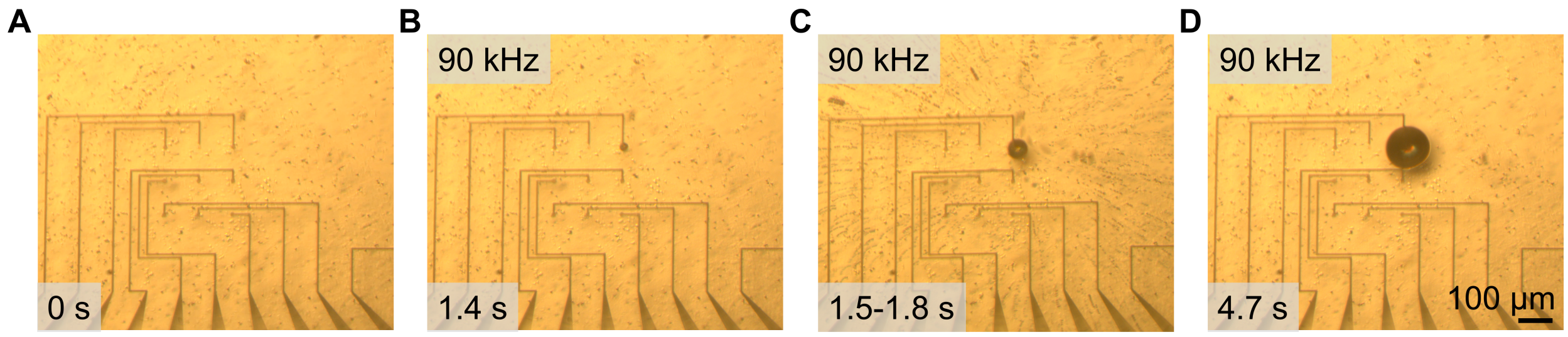

Figure S4: **Visualization of acoustic streaming during bubble growth under constant ultrasound excitation.** (**A**) Before bubble generation, no pronounced local tracer circulation was observed. (**B**) When the newly generated bubble remained small, it exhibited no appreciable ultrasound-driven oscillation, and no pronounced streaming developed. (**C**) As the bubble grew to a size range that could be effectively excited under the applied ultrasound, its interfacial oscillation generated pronounced local microstreaming, visualized using 5 μm tracer particles and superimposed frames. (**D**) Upon further growth, the bubble moved away from the effectively excited size range, and the streaming pattern disappeared. The ultrasound excitation frequency and voltage were maintained at 90 kHz and 20 V_{PP}, respectively.

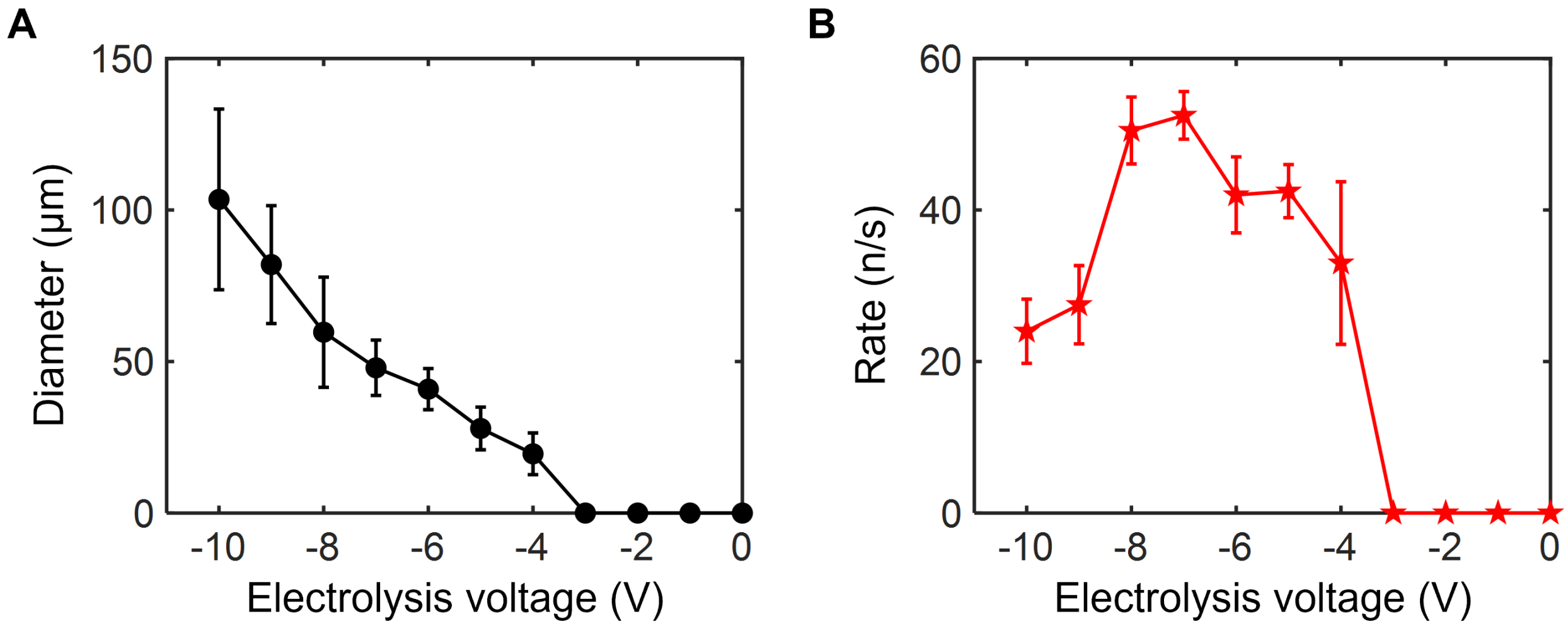

Figure S5: **Characterization of bubble size and bubbling rate as a function of electrolysis voltage.** (**A**) Bubble diameter as a function of electrolysis voltage. No bubbling was observed when the electrolysis voltage amplitude was below 4 V. (**B**) Bubbling speed as a function of electrolysis voltage. The bubbling speed increases with voltage for $|V| < 7$ V and decreases for $|V| > 7$ V. Error bars represent the mean ± s.d. ($n = 6$ independent measurements).

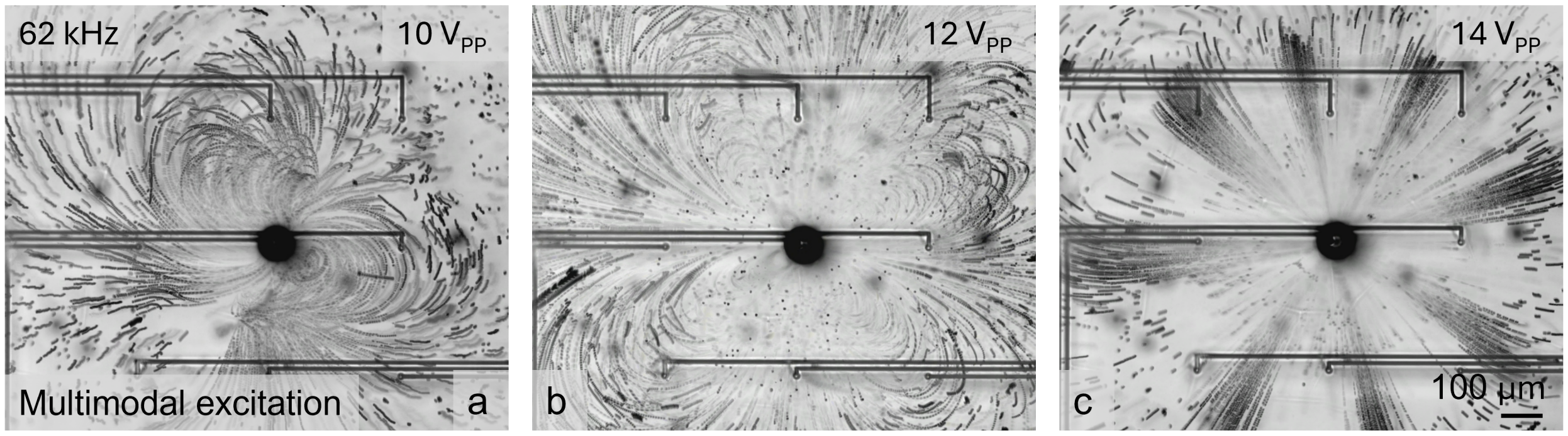

Figure S6: **Oscillation modes of a single bubble under different ultrasound excitation voltages.** A bubble with a constant diameter of 93 μm exhibited distinct oscillation modes at different ultrasound excitation voltages, including the (a) dipole mode, (b) quadrupole mode, and (c) octupole mode, while the ultrasound excitation frequency was fixed at 62 kHz.

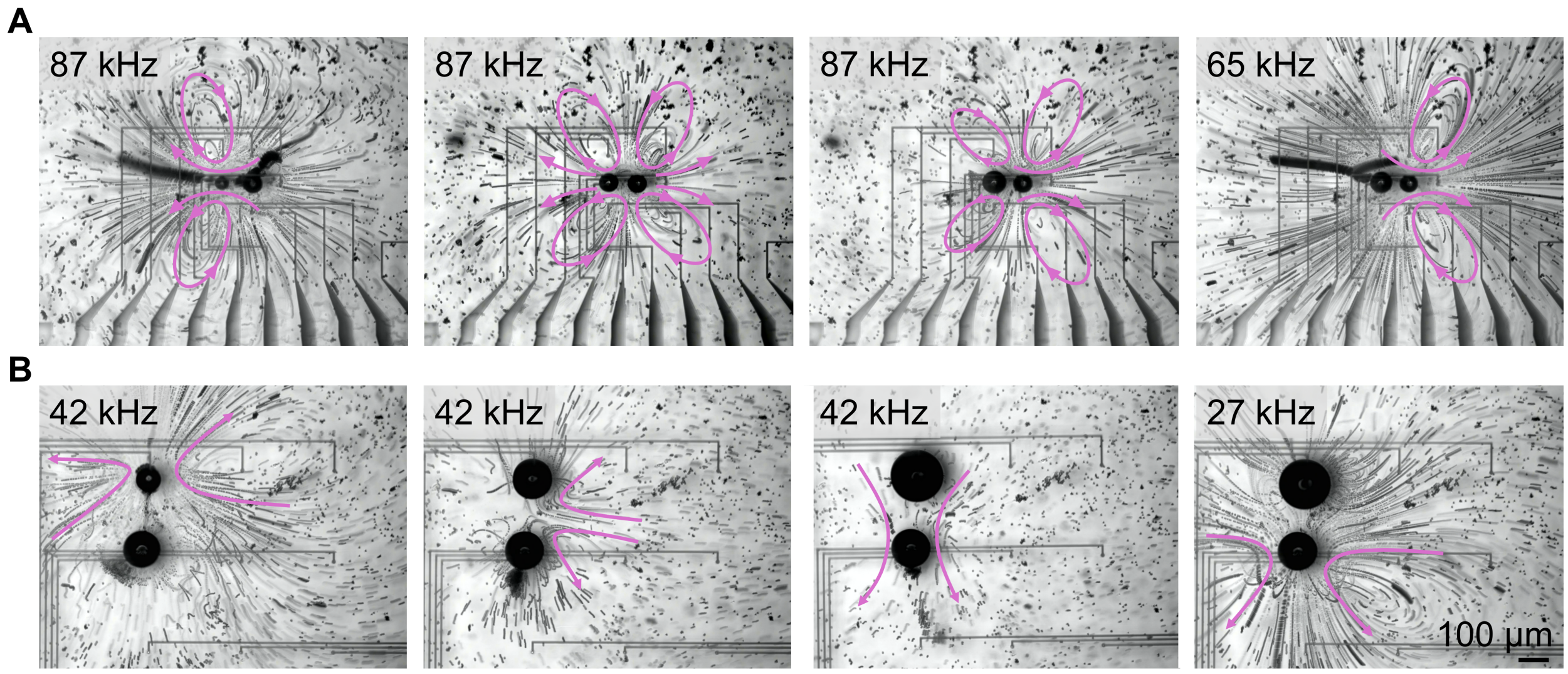

Figure S7: **Additional demonstrations of flow polarization using different bubble configurations and acoustic frequency modulation.** Dynamic flow flipping was achieved by gradually increasing the size of (**A**) the left bubble or (**B**) the top bubble in different bubble-pair configurations. After bubble growth and flow reversal, the ultrasound excitation frequency was adjusted to match the new bubble configuration and to enhance the resulting flow polarization. The magenta arrows indicate flow directions.

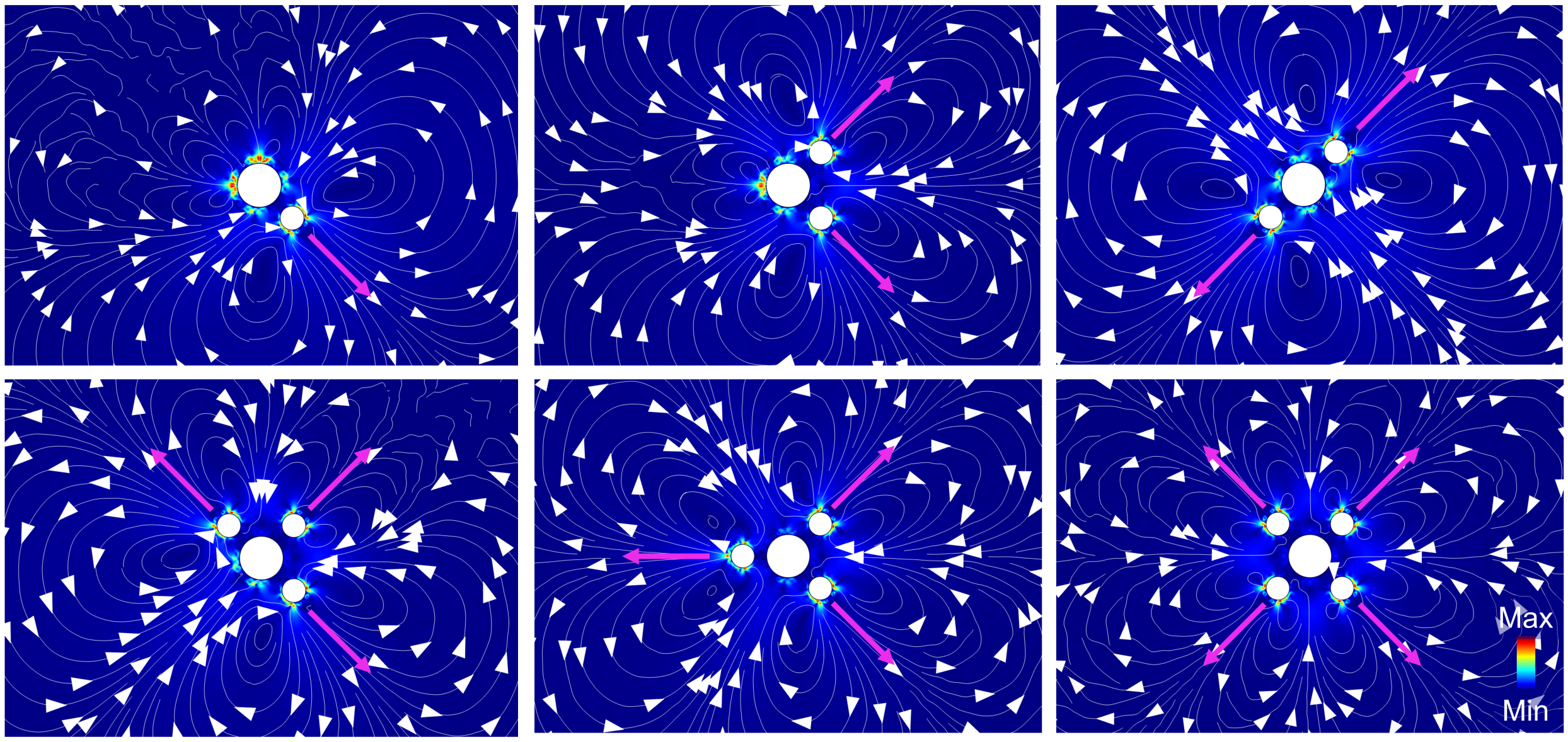

Figure S8: **Simulations of satellite-bubble induced polarized flows around a central bubble.** Satellite bubbles with a similar size surrounding a central bubble develop associated polarized flows. The color bar denotes the normalized flow speed. White and magenta arrows denote flow directions.

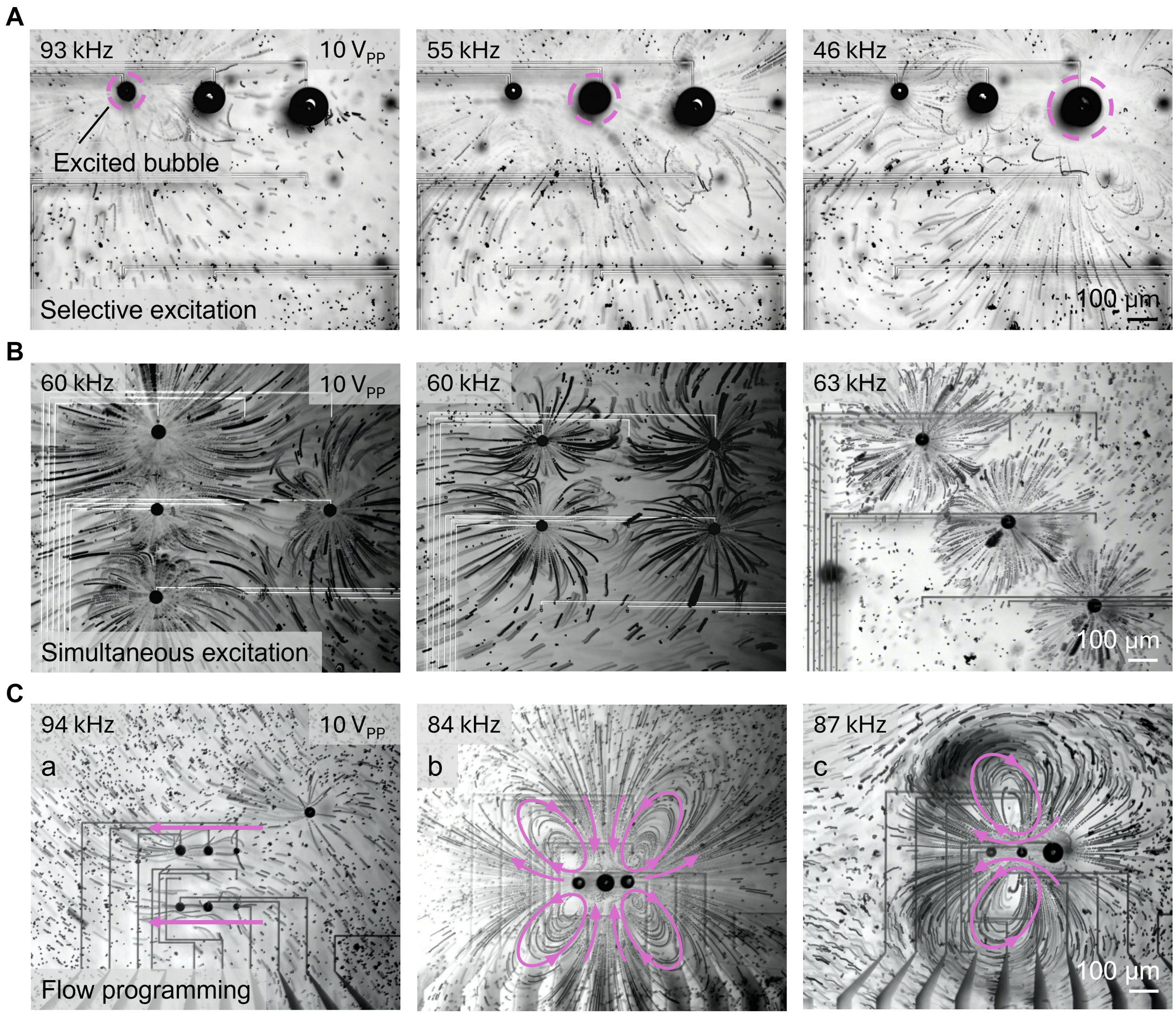

Figure S9: **Extended active bubble oscillation control and flow programming.** (**A**) Selective excitation of bubbles with different sizes at different ultrasound excitation frequencies. The magenta dashed circle indicates the excited bubble. (**B**) Simultaneous excitation of similarly sized bubbles arranged in different spatial configurations. (**C**) Programmable flow generation using multi-bubble patterns, including (a) flow pumping, (b) vortex generation, and (c) flow reorientation. The magenta arrows indicate flow directions.

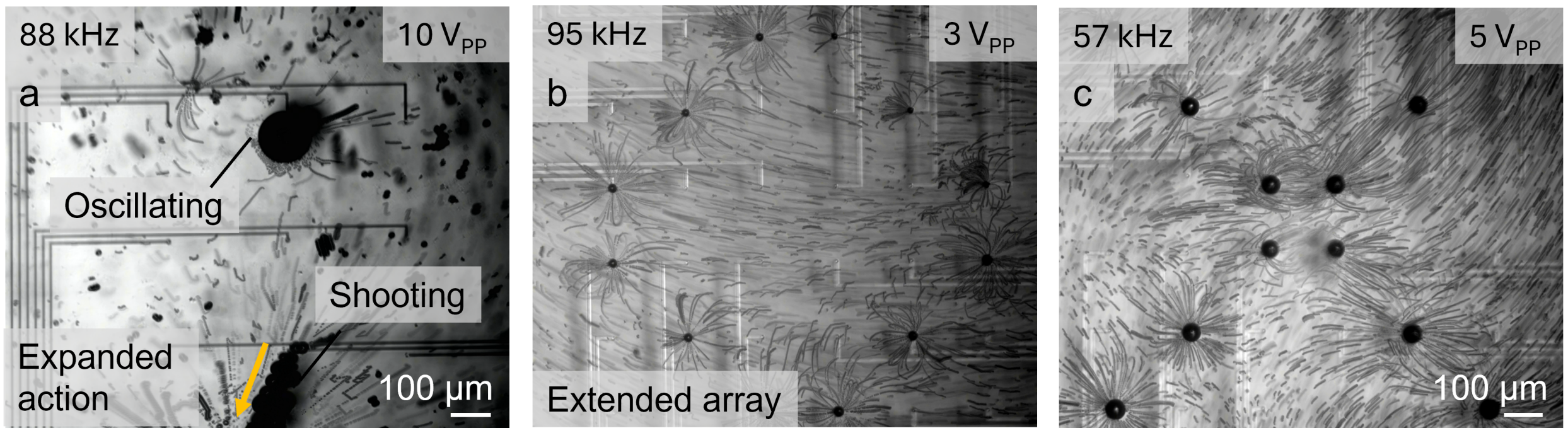

Figure S10: **Strengthened flow programming enabled by expanded manipulation actions and an extended electrode array.** (a) Simultaneous bubble oscillation and bubble shooting. (b) Circular bubble pattern and (c) X-shaped bubble pattern generated using an extended 6×6 electrode array.

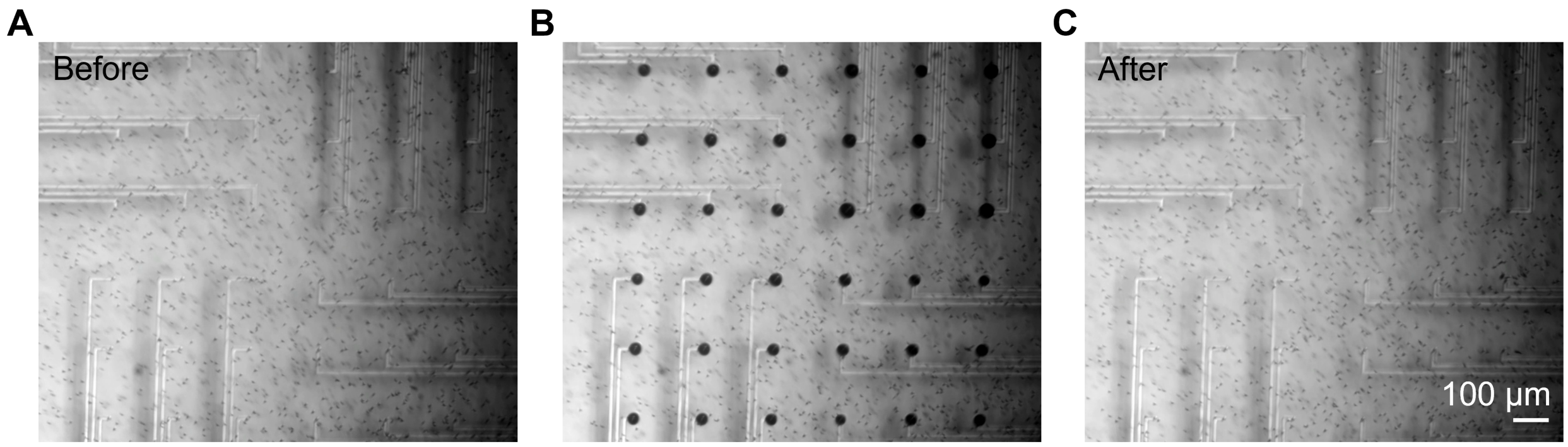

Figure S11: **Global erasure of 36 microbubbles.** (**A**) Image of the electrode array prior to bubble generation. (**B**) Image showing the generation of 36 microbubbles on the electrode array. (**C**) Image of the electrode array after bubble erasure. Ultrasound was at 57 kHz and 20 V_{PP}.

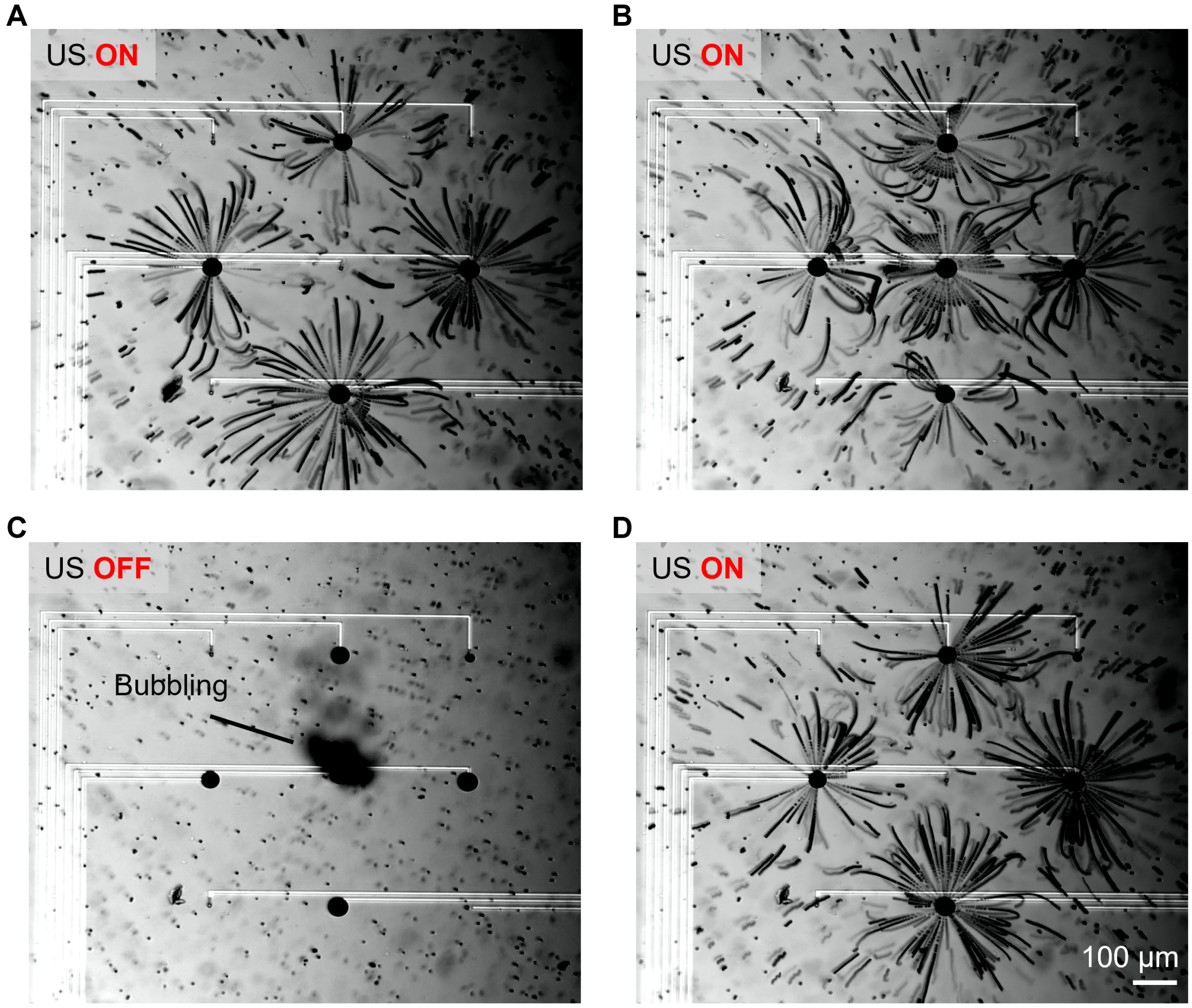

Figure S12: **Selective removal of a microbubble via localized bubbling.** (**A**) Initially, four bubbles were generated and excited under ultrasound at 60 kHz and 5 V_{PP}. (**B**) The fifth bubble was then generated at the center of the array. (**C**) To remove the central bubble, the ultrasound was turned off, and localized bubbling was performed at the central electrode. (**D**) Finally, the targeted bubble was removed while the surrounding bubbles remained stably oscillating. Flow fields were visualized using 5 μm-diameter tracer particles and superimposed frames.

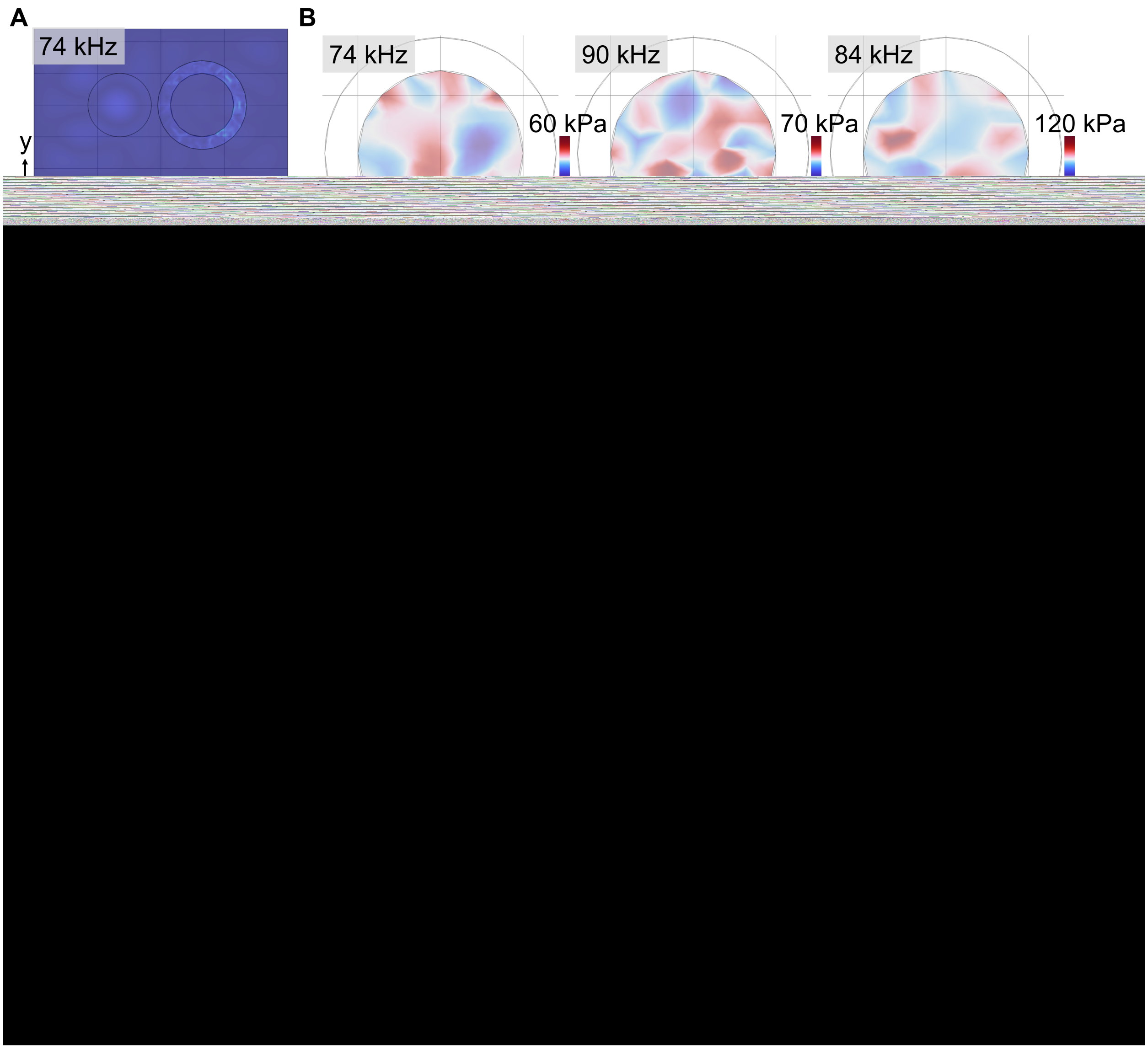

Figure S13: **Simulations of substrate vibration and acoustic pressure distribution within the acoustic chamber.** (**A**) Simulated vibration of the substrate. The color bar denotes the displacement magnitude. Simulated acoustic pressure distribution at the (**B**) top surface (air-liquid interface) and (**C**) bottom surface (liquid-substrate interface) at the indicated frequencies under 20 V_{PP} excitation. Color bars denote acoustic pressure amplitudes. The simulations are used to identify the frequency-sensitive evolution of the chamber acoustic field and the associated force mechanism, rather than to claim an exact point-by-point reproduction of the experimental pressure field.

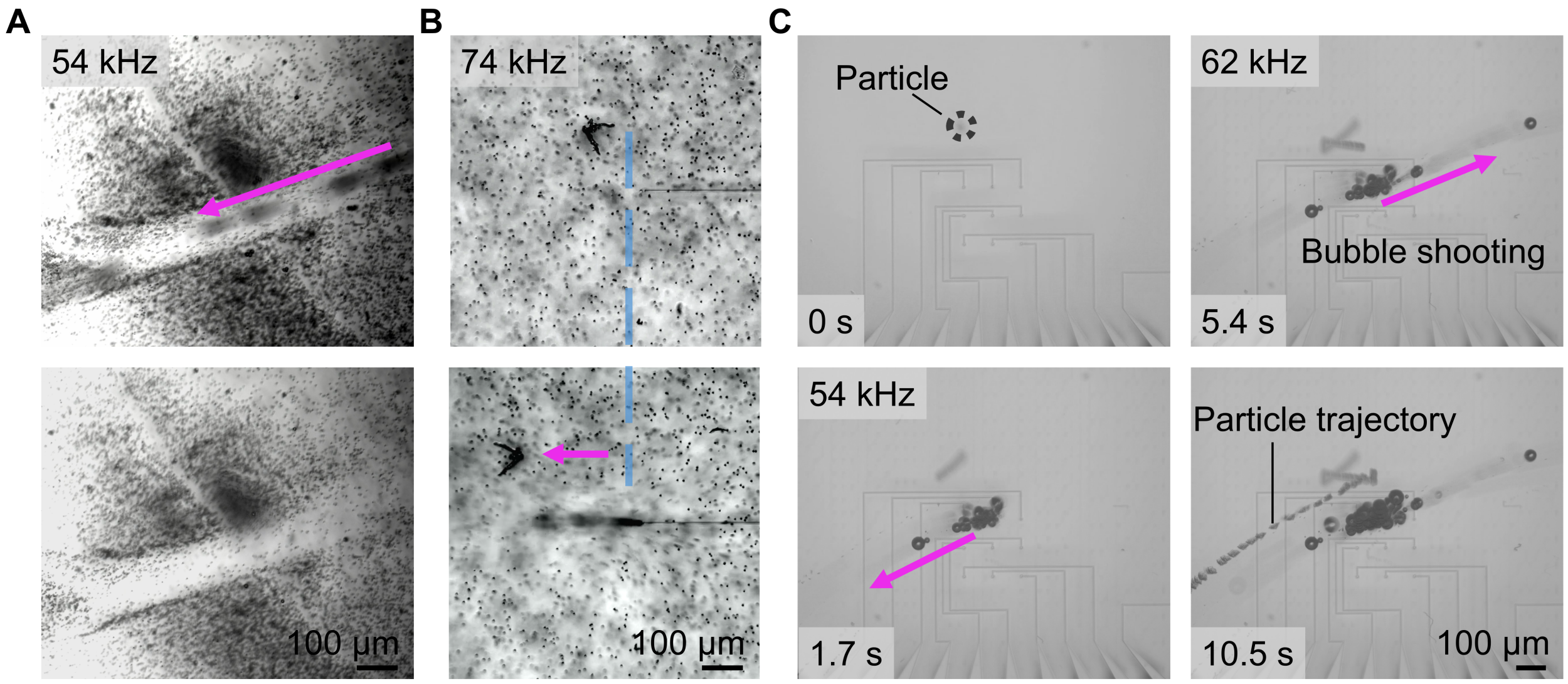

Figure S14: **Functional demonstrations of directional bubble shooting.** (**A**) Surface cleaning: left-bottom shooting bubbles removed particles deposited on the substrate. (**B**) Cluster transport: left directional shooting bubbles displaced a cluster. The blue dashed line denotes the initial position of the cluster. (**C**) Frequency-selective particle routing: a target particle was transported to the left or right by selectively tuning the ultrasound excitation frequencies. Magenta arrows denote bubble shooting directions. The black dashed line denotes the initial position of the target particle. The ultrasound excitation voltage was 20 V_{PP}.

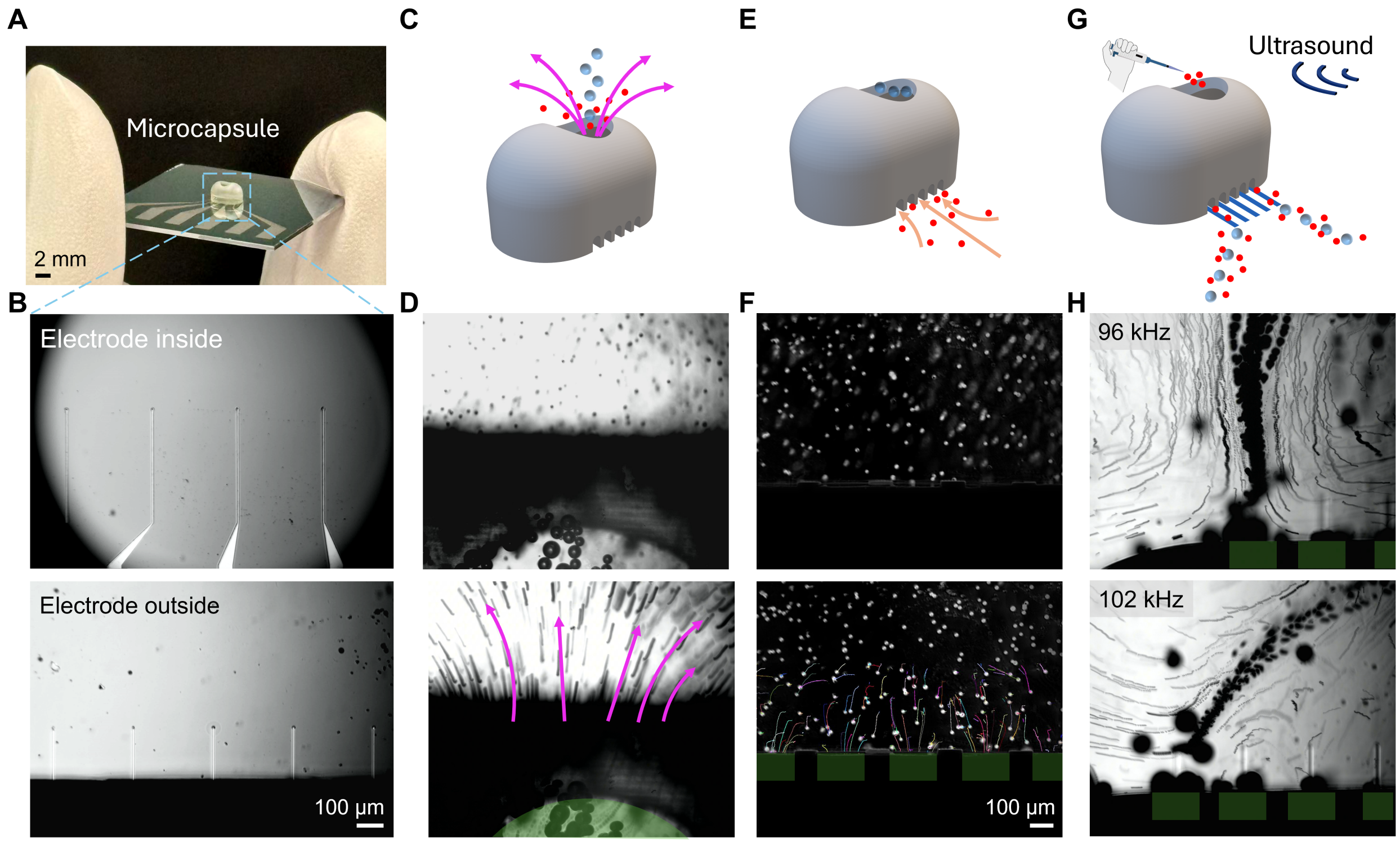

Figure S15: **A multifunctional microcapsule enabled by programmable bubble shooting.** (**A**) Optical image of a microcapsule printed by two-photon polymerization on a Si substrate with electrode arrays. The microcapsule has dimensions of 4000 × 2500 × 2250 × 250 μm in length, width, height, and wall thickness, respectively. It contains a circular top opening with a diameter of 1 mm and five bottom semicircular openings with a diameter of 100 μm, each aligned with a microelectrode. (**B**) Electrodes positioned inside (top panel) and outside (bottom panel) the capsule for different operating modes. (**C**) Schematic of preloaded cargo released from the top opening driven by bubbling from internal electrodes. (**D**) An image before bubbling and the superimposed image of particle release during bubbling at −5V using five electrodes. (**E**) Schematic of particle collection through the bottom openings driven by bubbling from internal electrodes. (**F**) Images before bubbling and particle trajectories during bubbling at −8V using five electrodes. (**G**) Schematic of selective directional release of preloaded contents through programmable bubble shooting by switching the ultrasound excitation frequencies. (**H**) Superimposed images showing directional bubble shooting at 96 and 102 kHz, respectively. The ultrasound excitation voltage was 5 V_{PP}, and the electrolysis voltage was −4V. Magenta arrows denote flow directions. Green rectangles denote the positions of openings.

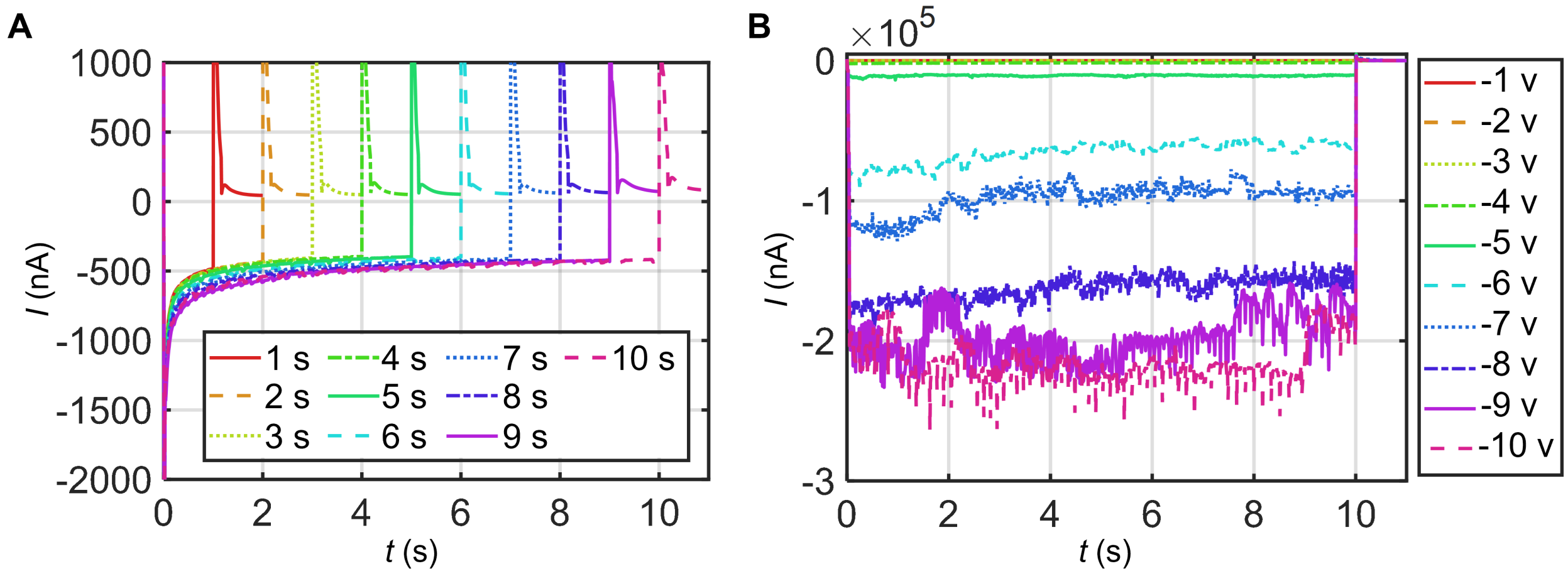

Figure S16: **Electrolysis current as a function of electrolysis time under different electrolysis voltages.** (**A**) The electrolysis current was measured using an electrochemical workstation under working-electrode voltages of −3.3 V with electrolysis durations ranging from 1 s to 10 s. (**B**) The current magnitude increased with the applied voltage magnitude. Observable bubbling occurred when the voltage magnitude exceeded approximately 4 V, whereas no bubble generation was observed at −1 V or −2 V.

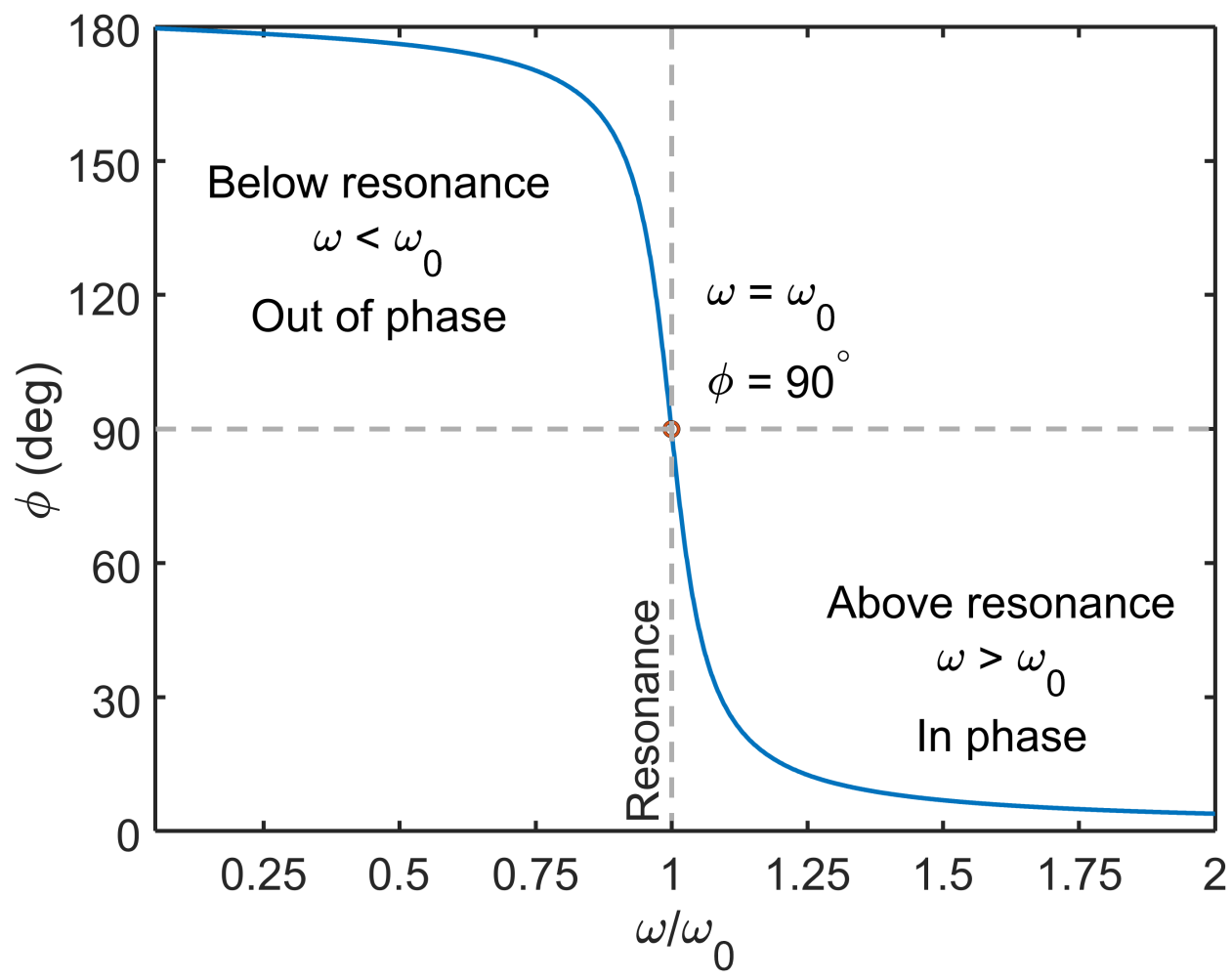

Figure S17: **Phase difference between bubble radial oscillation and external acoustic pressure as a function of normalized driving frequency.** The phase decreases from 180° below resonance to 0° above resonance, passing through 90° at $\omega = \omega_0$.

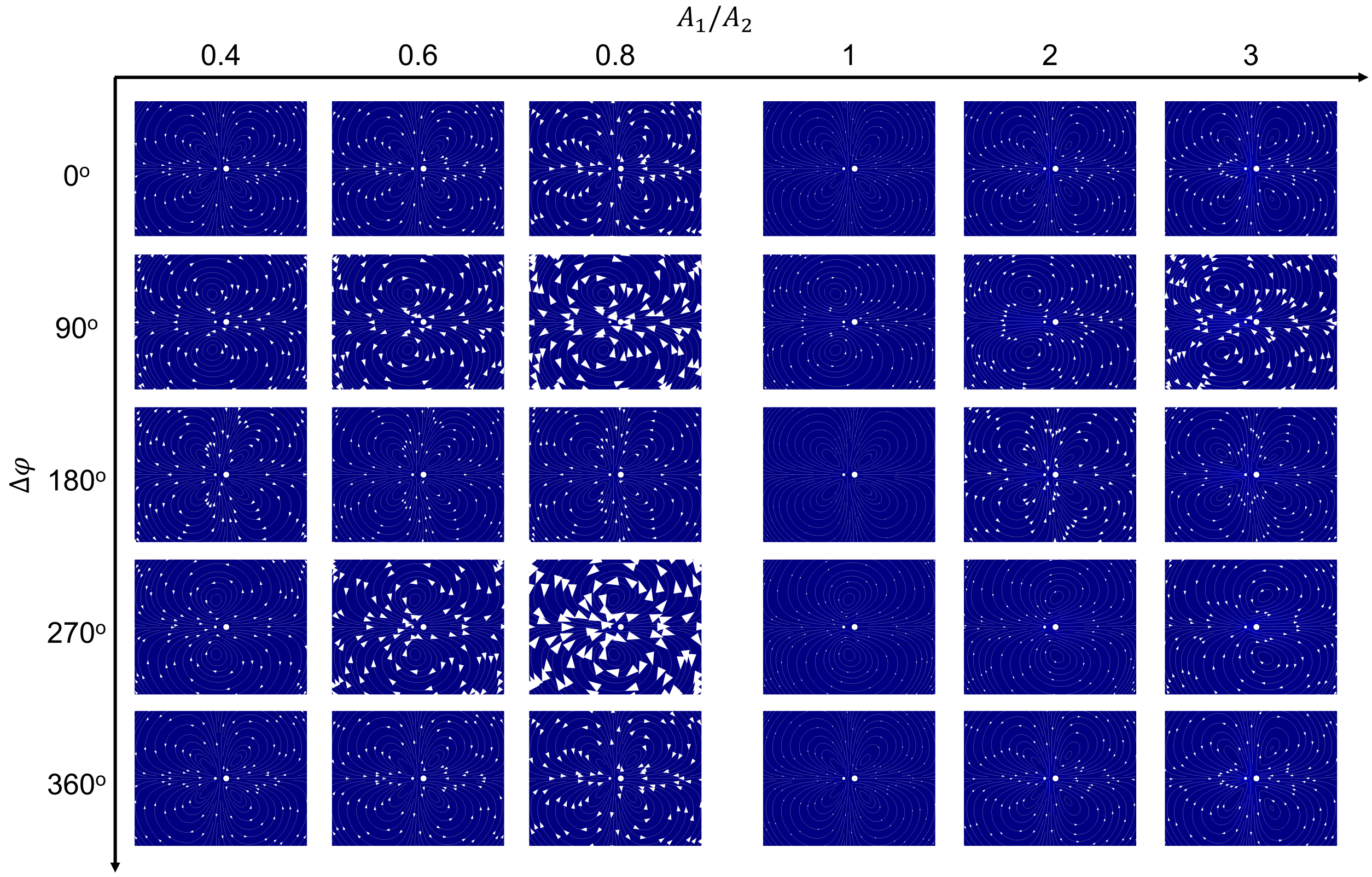

Figure S18: **Simulated acoustic-streaming states of a two-bubble configuration under parametric sweeps of oscillation-amplitude ratio and phase difference.** The two bubble diameters were prescribed as 27.5 μm and 55 μm, respectively. The oscillation-amplitude ratio (A_1/A_2) primarily modulates the relative streaming strength, whereas the phase difference ($\Delta\varphi$) changes the streaming topology. The color maps denote the scaled flow speed. For visualization, two separate arrow scales were used for oscillation-amplitude ratios of 0.4–0.8 and 1–3, because the latter produces substantially higher flow speeds than the former.

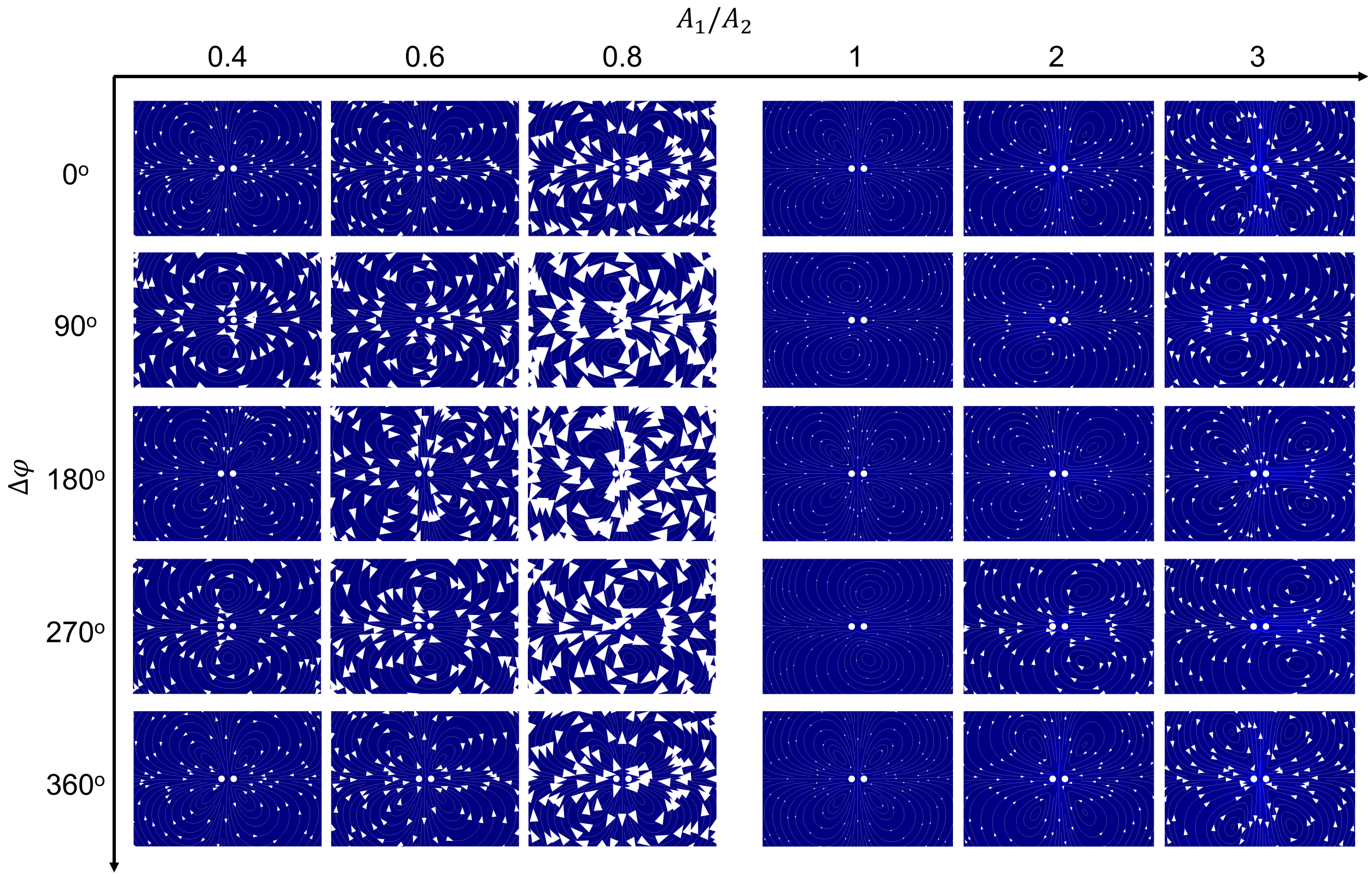

Figure S19: **Simulated acoustic-streaming states of a two-bubble configuration under parametric sweeps of oscillation-amplitude ratio and phase difference.** The two bubble diameters were prescribed as 55 μm and 55 μm, respectively. The oscillation-amplitude ratio (A_1/A_2) primarily modulates the relative streaming strength, whereas the phase difference ($\Delta\varphi$) changes the streaming topology. The color maps denote the scaled flow speed. For visualization, two separate arrow scales were used for oscillation-amplitude ratios of 0.4-0.8 and 1-3, because the latter produces substantially higher flow speeds than the former.

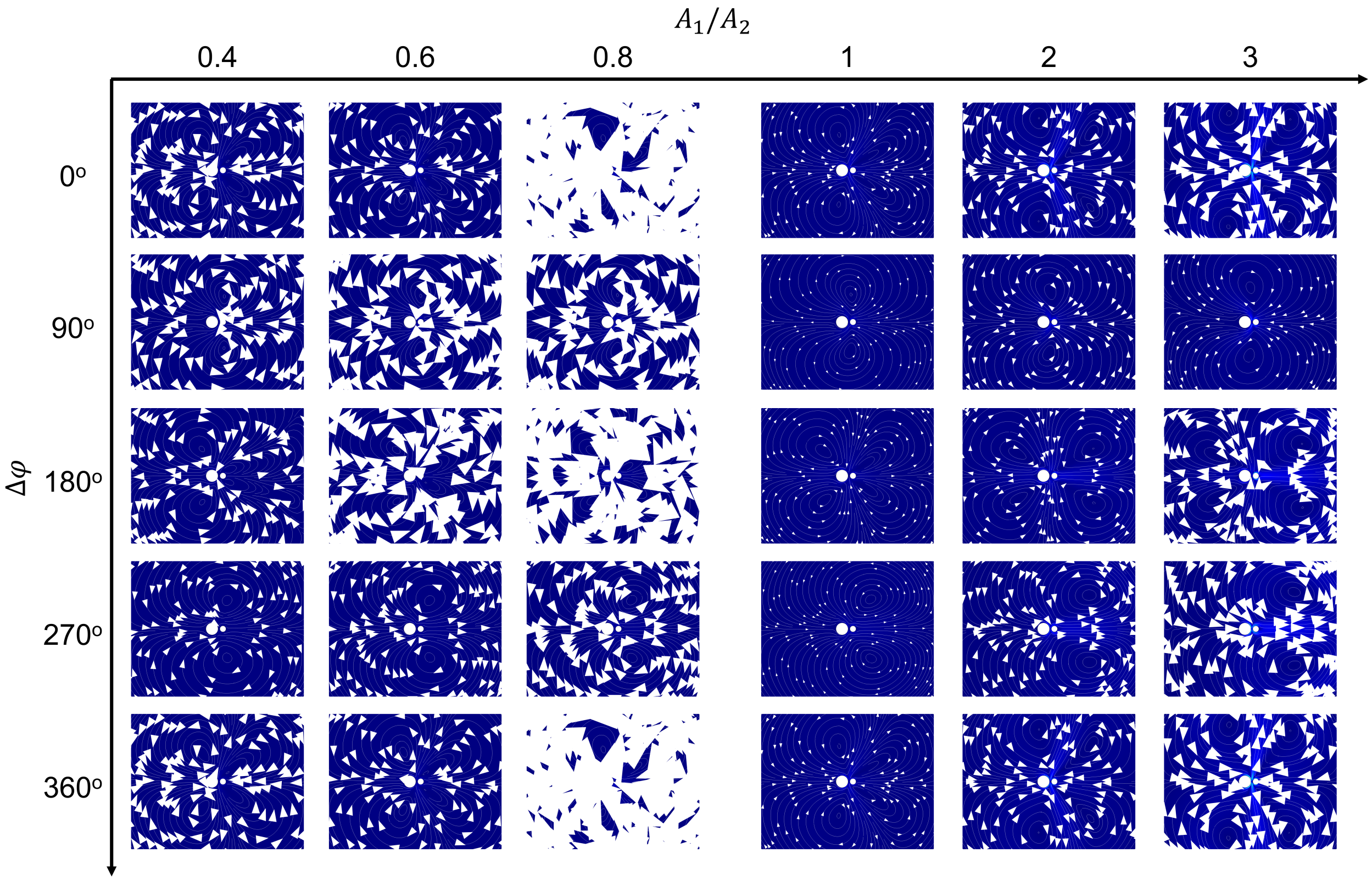

Figure S20: **Simulated acoustic-streaming states of a two-bubble configuration under parametric sweeps of oscillation-amplitude ratio and phase difference.** The two bubble diameters were prescribed as 110 μm and 55 μm, respectively. The oscillation-amplitude ratio (A_1/A_2) primarily modulates the relative streaming strength, whereas the phase difference ($\Delta\varphi$) changes the streaming topology. The color maps denote the scaled flow speed. For visualization, two separate arrow scales were used for oscillation-amplitude ratios of 0.4-0.8 and 1-3, because the latter produces substantially higher flow speeds than the former.

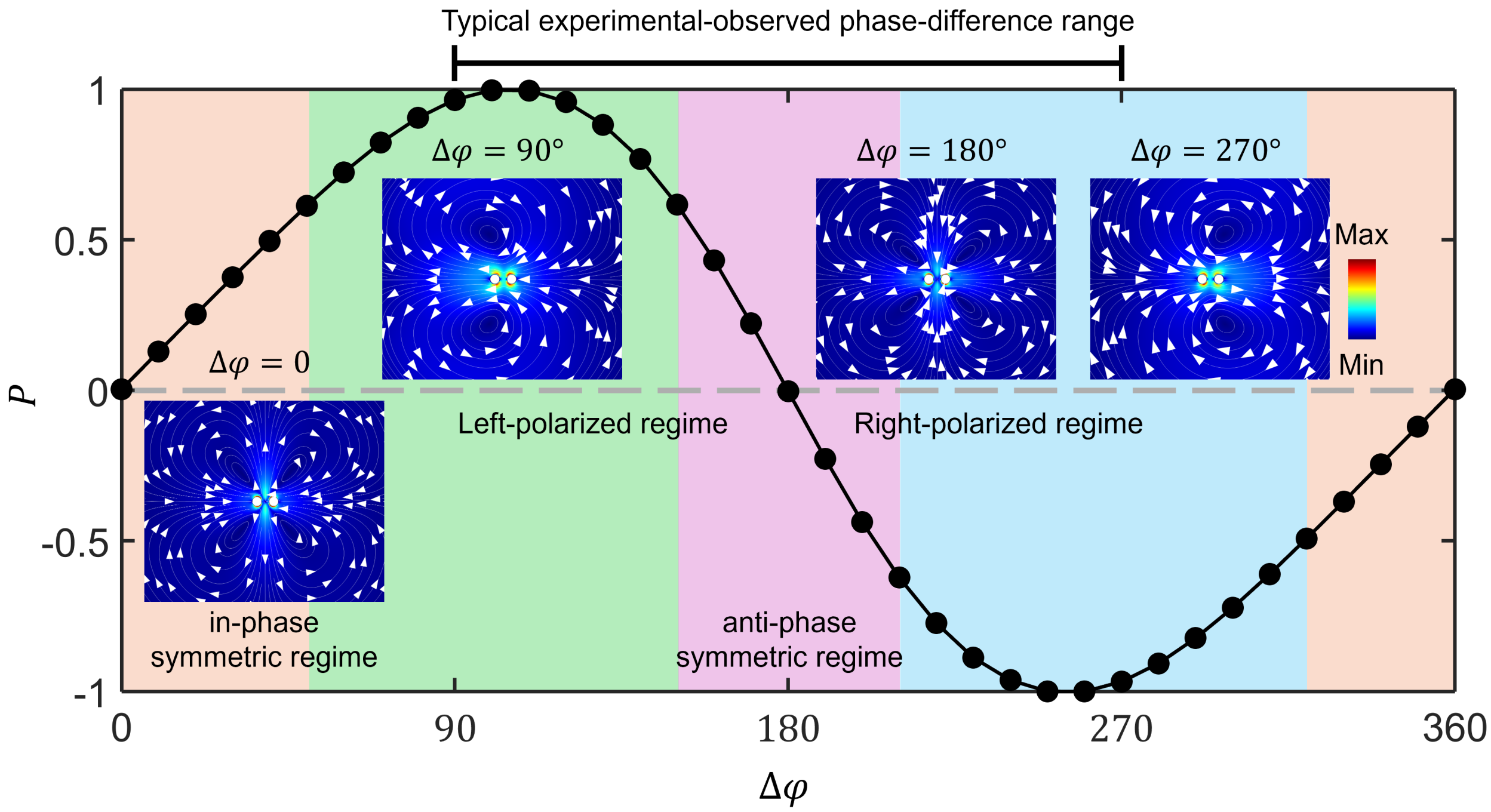

Figure S21: **Phase diagram of acoustic-streaming states as a function of the phase difference between two bubbles.** P is the mean horizontal velocity along the centerline between the two bubbles. The insets show simulated acoustic-streaming fields with the phase differences of 0°, 90°, 180°, and 270°, respectively. The color bar denotes the scaled flow speed. From left to right, the four background colors indicate the in-phase symmetric regime, left-polarized regime, anti-phase symmetric regime, and right-polarized regime, respectively.

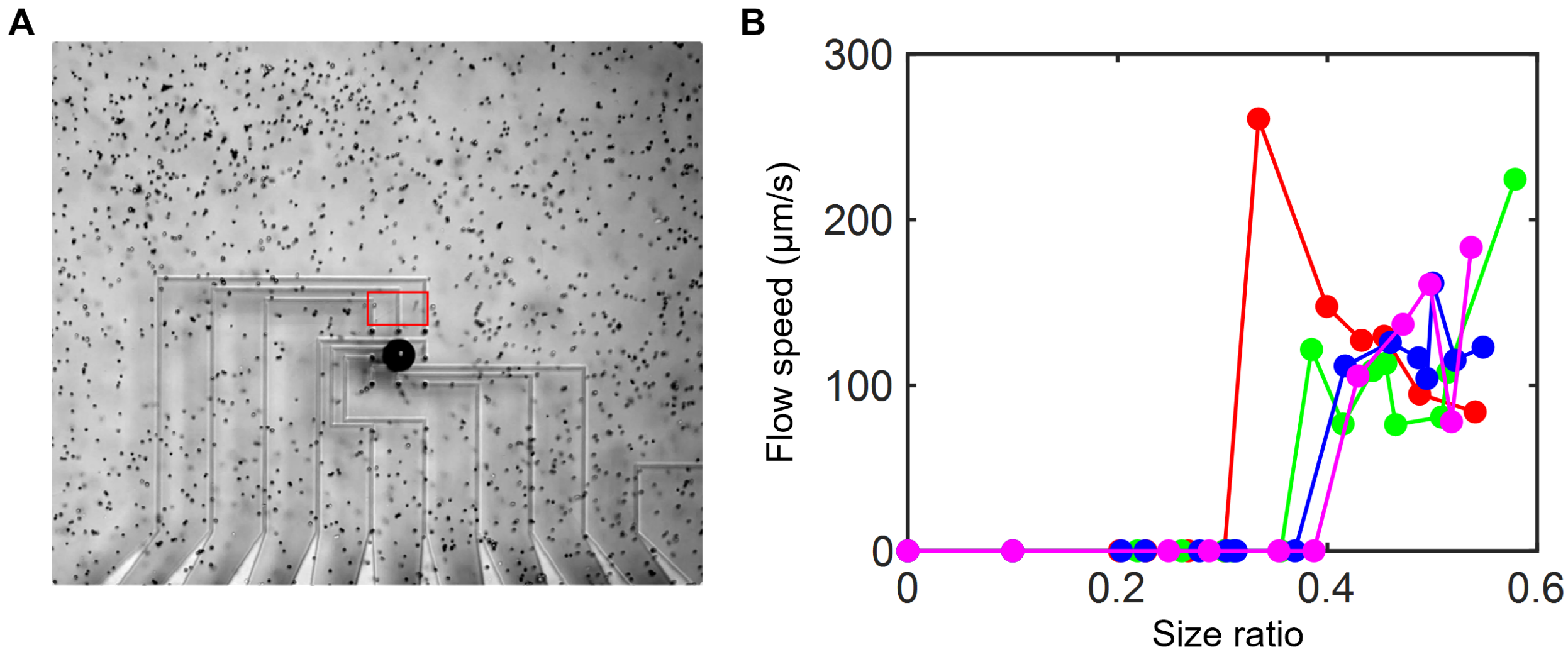

Figure S22: **Quantification of polarization flow speed.** (**A**) The flow speed was quantified as the average optical-flow speed within the selected region of interest (ROI). For each polarization direction, the ROI was repositioned in the corresponding direction. (**B**) Flow speed of the polarized flow as a function of the size ratio of the bubble pair shown in **Fig. 3B**. Different colors denote different bubble pairs.

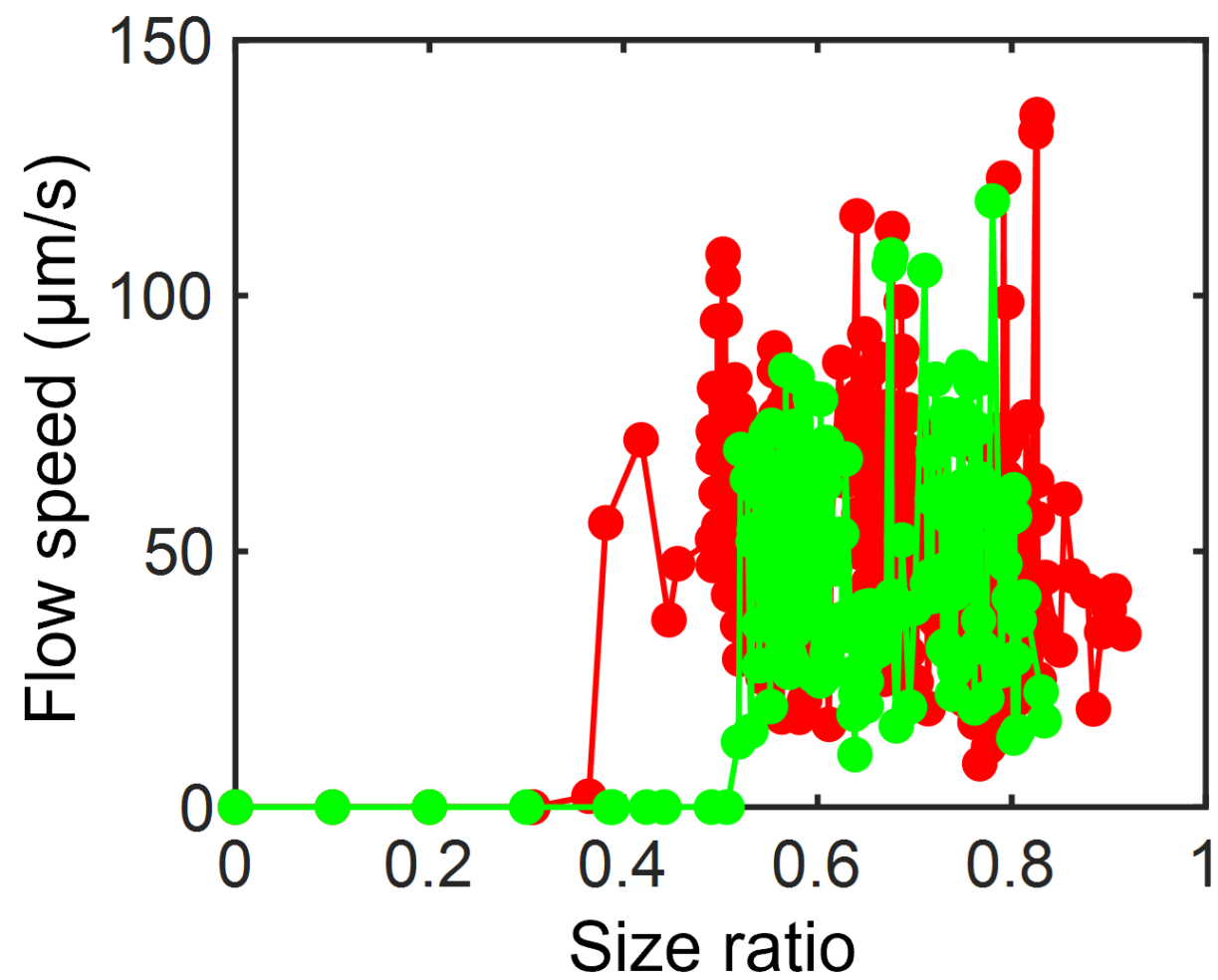

Figure S23: **Flow speed of the polarized flow as a function of the size ratio of the bubble pair shown in Fig. 3A.** The flow speed was quantified as the average optical-flow speed within the selected ROI. The collected flow velocity data were denoised using a low-pass filter with a cutoff frequency of 5 Hz. Different colors denote different bubble pairs.

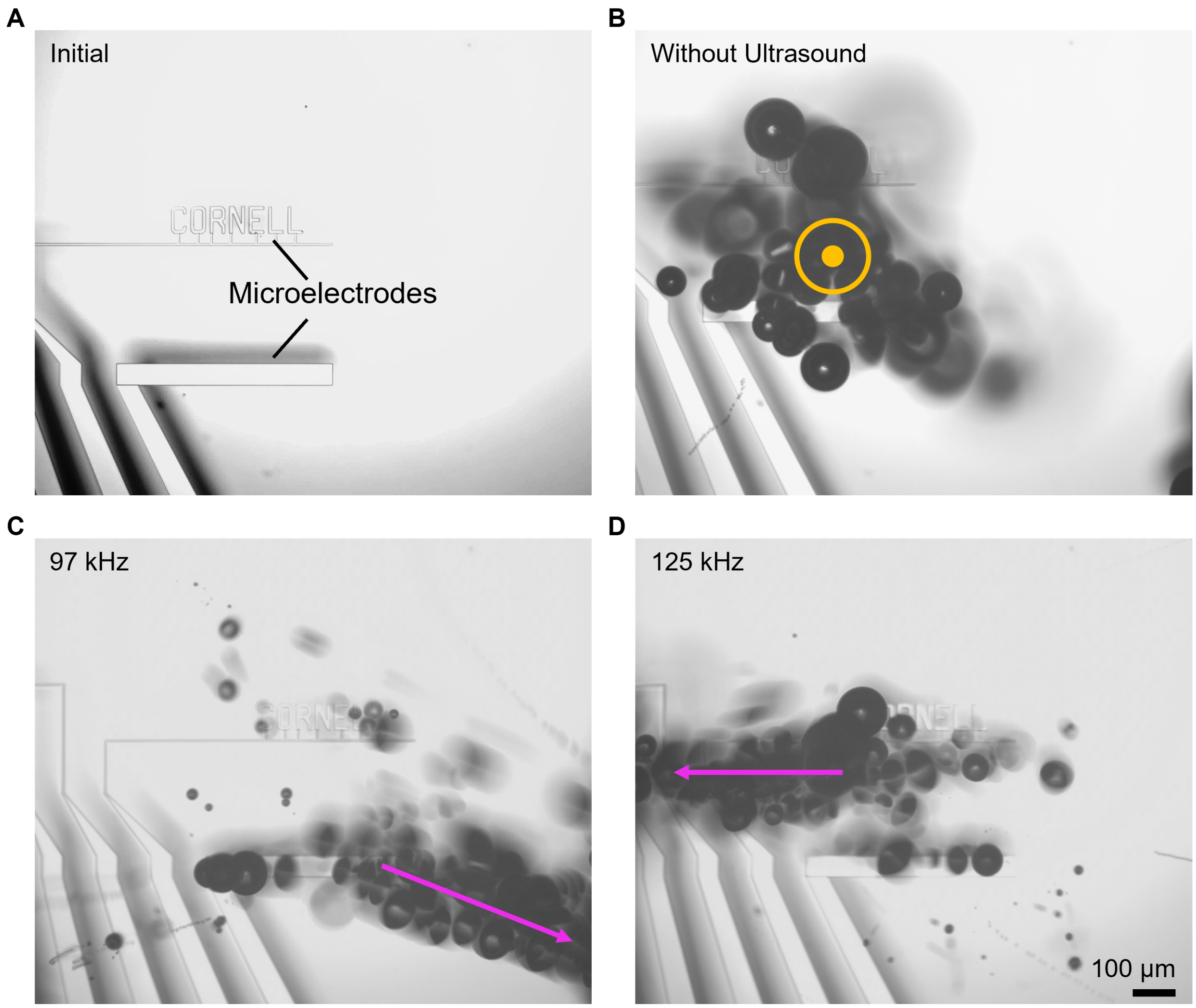

Figure S24: **Directed bubble shooting generated by microelectrodes with different exposed dimensions.** (**A**) Image of microelectrodes with varying exposed dimensions and geometries, generating different bubble detachment conditions and a range of detachment sizes. (**B**) Superimposed images show that in the absence of ultrasound, bubbles generated from all electrodes rose to the liquid-air interface without exhibiting near-wall directional shooting. Superimposed images show that under (**C**) 97 kHz excitation, bubbles detached from different electrodes were shot toward the bottom-right, whereas under (**D**) 125 kHz excitation, bubbles primarily shot toward the left. The ultrasound excitation voltage was fixed at 20 V_{PP}.

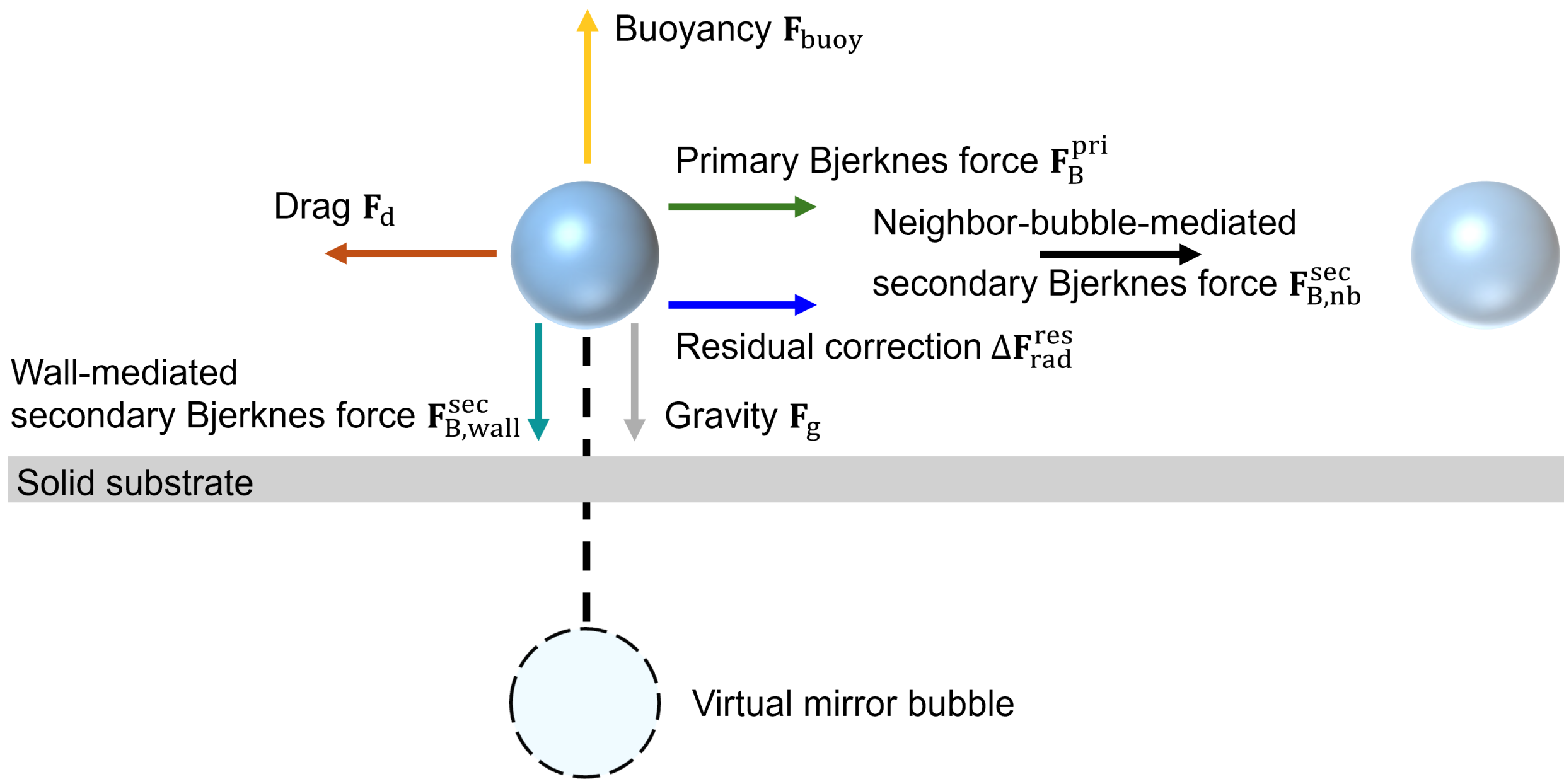

Figure S25: **Schematic force analysis for an ultrasound-excited bubble in liquid near a solid substrate after detachment.**

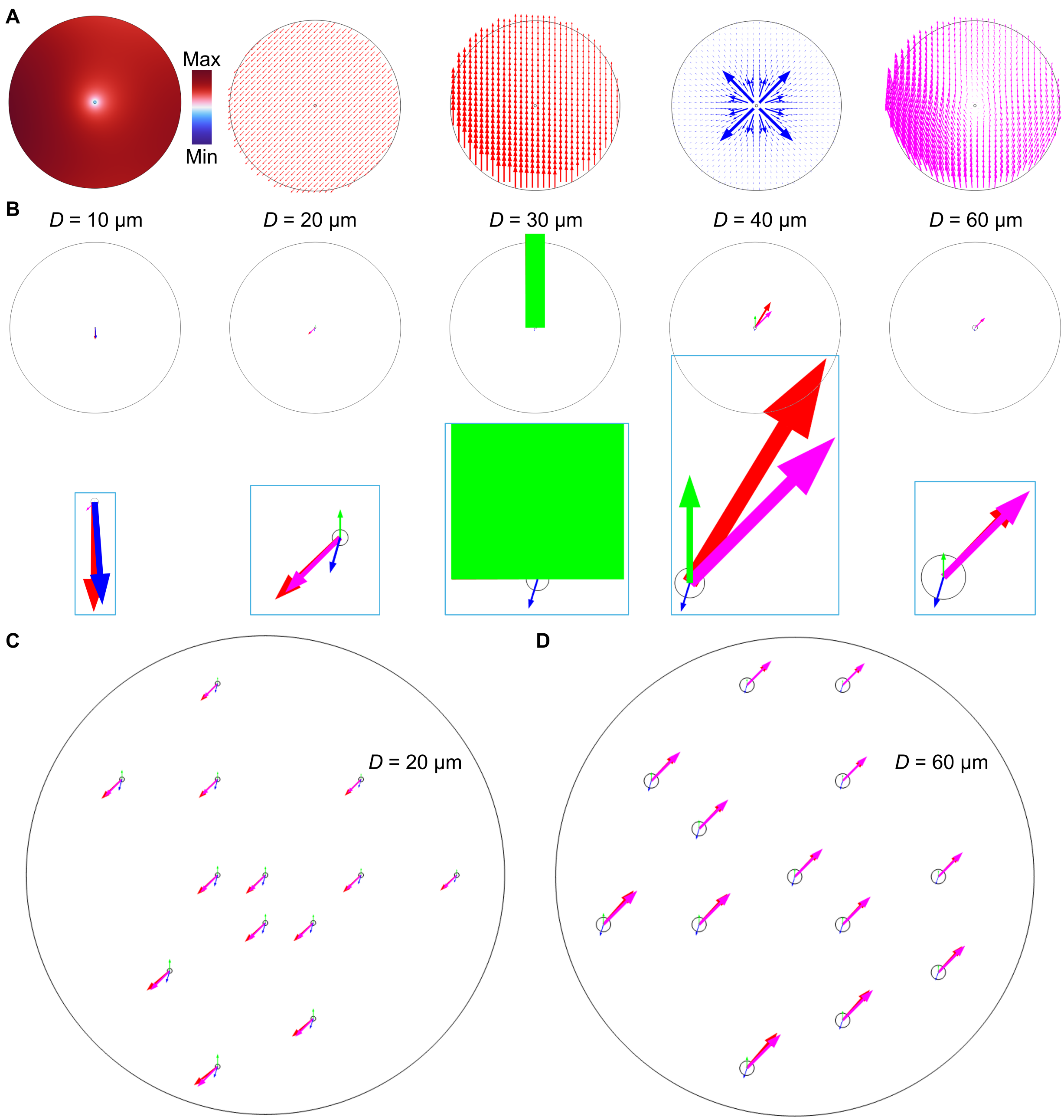
A
Max
Min
B
D = 10 μm
D = 20 μm
D = 30 μm
D = 40 μm
D = 60 μm
C
D = 20 μm
D
D = 60 μm

Figure S26: **Simulations of acoustic fields and force contributions for bubbles in a prescribed traveling wave field. (A)** A wave propagating along $+y$ with the pressure-gradient axis inclined at 45°, by setting $\alpha = -300\ \mathrm{m}^{-1}$, $\theta_k = 90°$, and $\theta_g = 45°$. From left to right, the panels are the acoustic pressure distribution, acoustic pressure gradient, background field acoustic-intensity vectors, scattered field acoustic-intensity vectors, and total field acoustic-intensity vectors. **(B)** Acoustic radiation force analysis. Magenta arrows denote the pressure-gradient component of the primary Bjerknes force, green arrows denote the phase-gradient component of the primary Bjerknes force, blue arrows denote the residual momentum-flux correction, and red arrows denote the resultant total radiation force. Light blue boxes show enlarged views of the force vectors. A diameter of 30 μm corresponds to the resonant diameter. **(C, D)** Resultant force vectors for 20 μm and 60 μm bubbles, respectively. Across the entire sampling region, the direction of the force exerted on bubbles of the same size remains virtually identical.

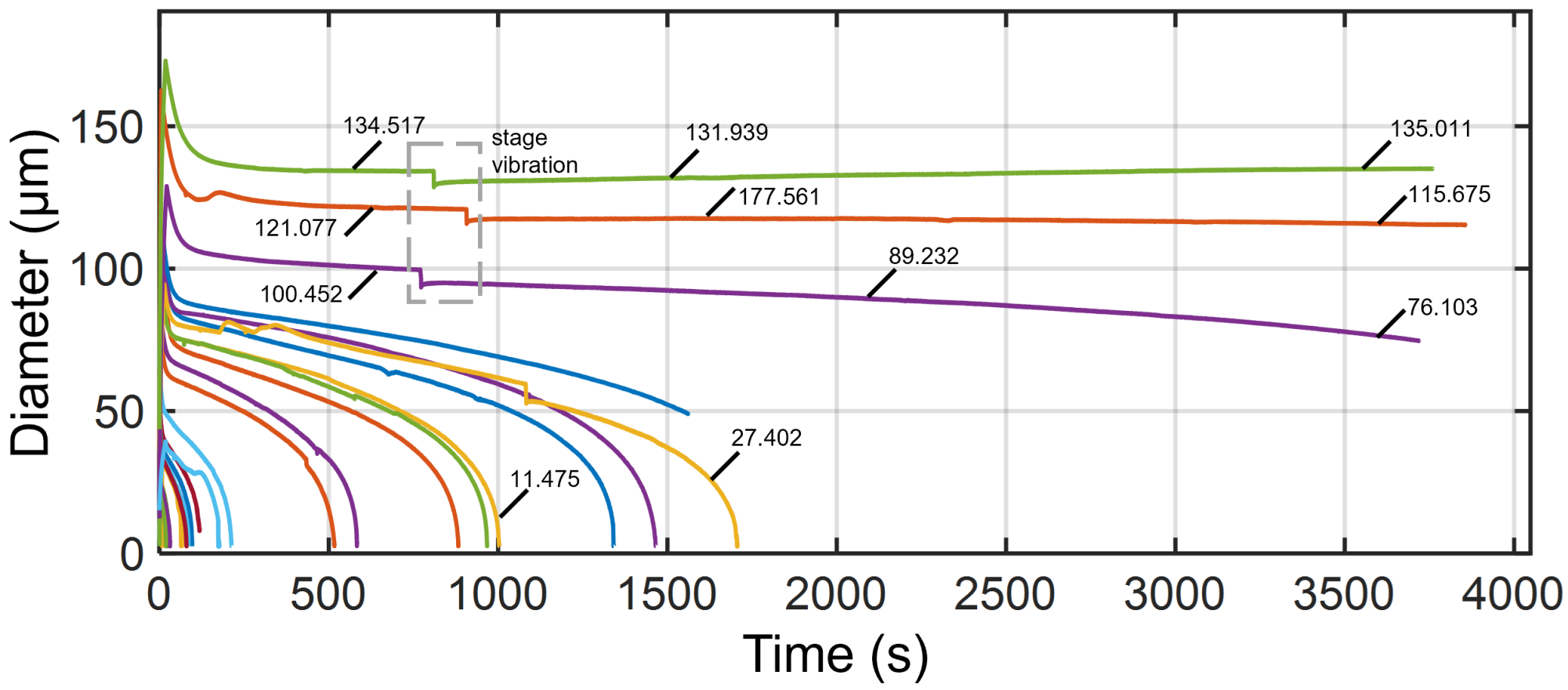

Figure S27: **Bubble diameter as a function of time after electrolysis is turned off.** Without ultrasound excitation, newly generated bubbles underwent size relaxation after electrolysis was stopped. Larger bubbles shrank briefly and then maintained a relatively stable size, whereas smaller bubbles shrank more rapidly and eventually disappeared. The abrupt jumps and fluctuations in some diameter traces are attributed mainly to image-detection errors, stage vibration, and optical distortion caused by liquid evaporation during long-term observation.

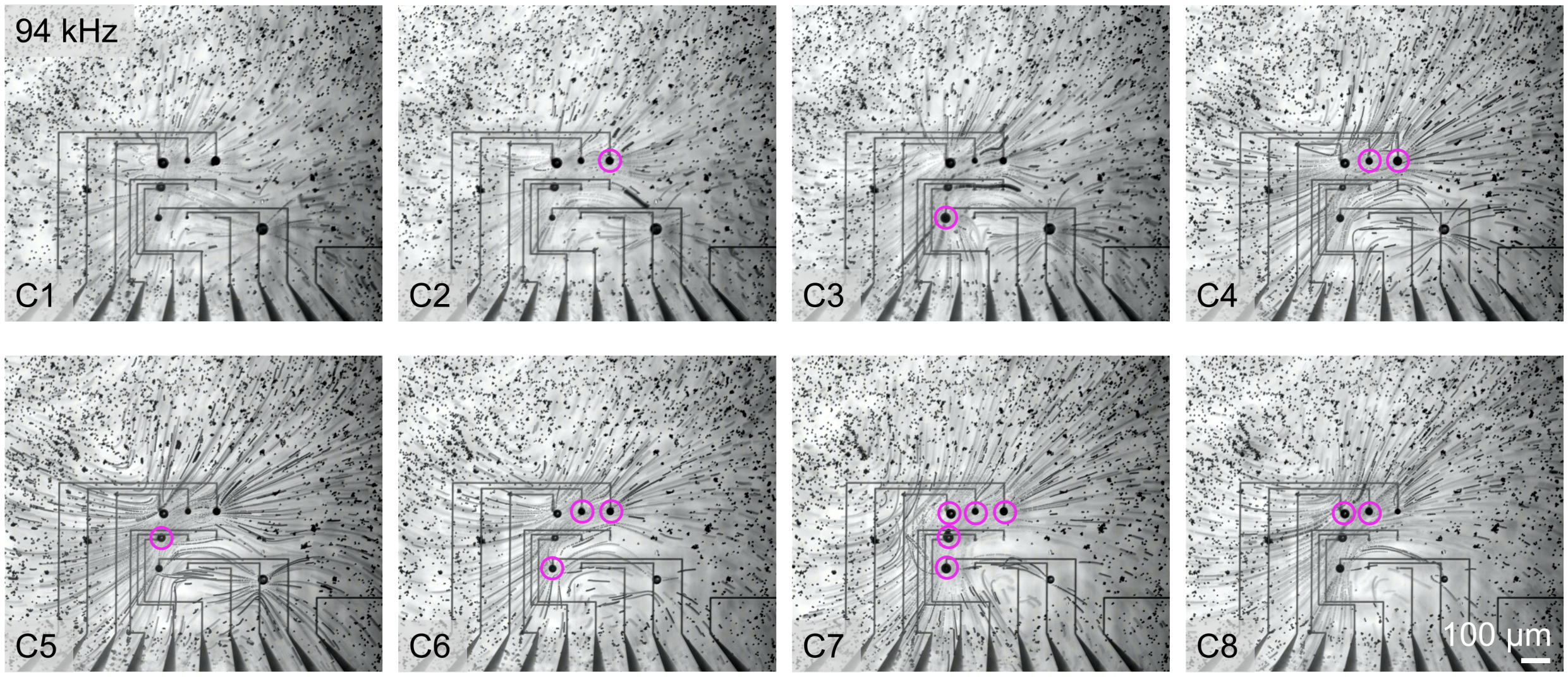

Figure S28: **Maintenance of a programmed flow state through active bubble refreshing by localized electrolysis.** The programmed turning-flow state was maintained by reapplying localized electrolysis to refresh bubbles that had shrunk during operation. Each panel represents a refreshing cycle. Magenta circles indicate the bubble refreshed in the corresponding cycle. Experiments were performed under ultrasound excitation at 94 kHz and 10 V_{PP}. C*i* indicates the current cycle.

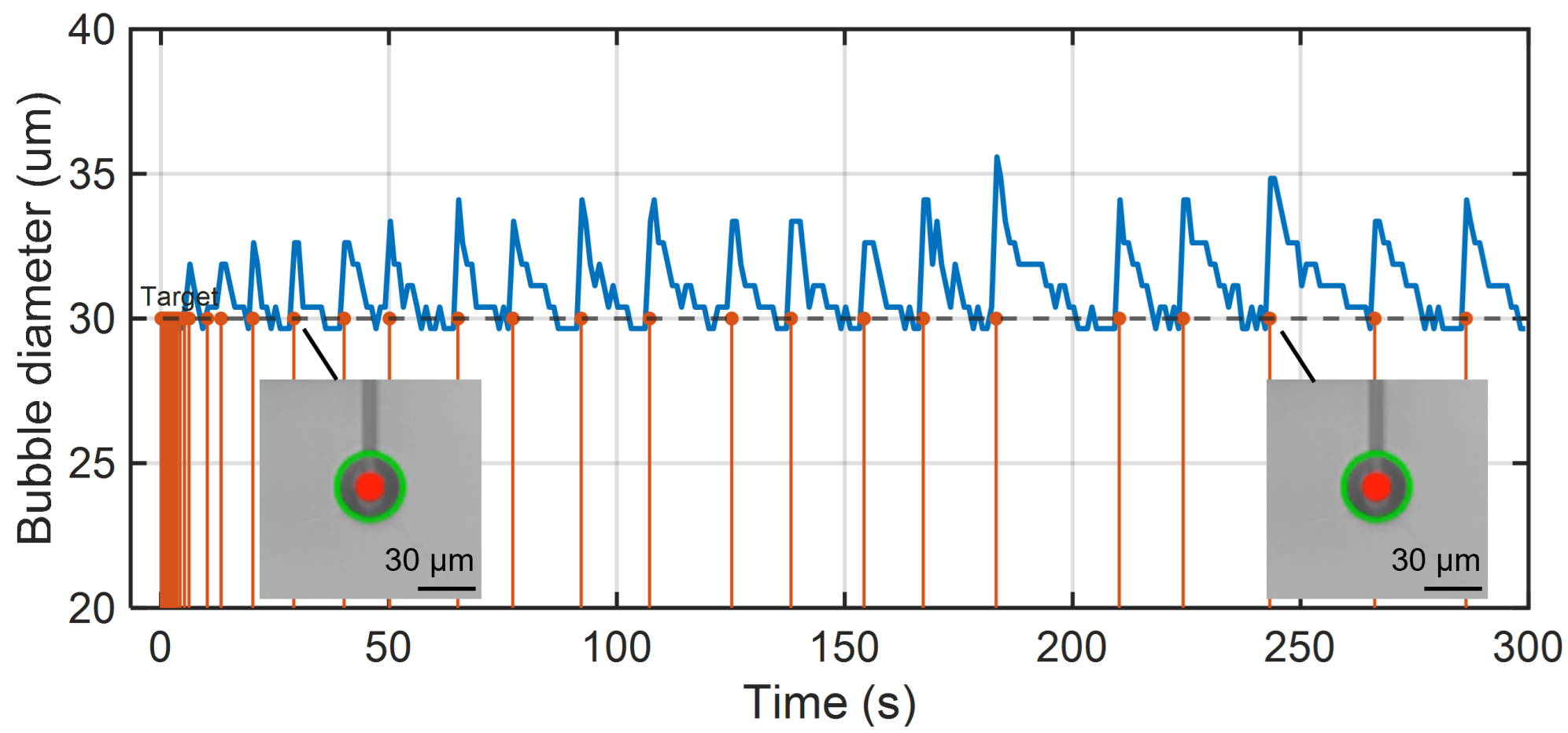

Figure S29: **Image-based closed-loop control of bubble diameter.** The bubble diameter was maintained near a prescribed target value of 30 μm using event-triggered electrolysis pulses based on real-time optical feedback. The insets are real-time views of the bubble diameter detection.

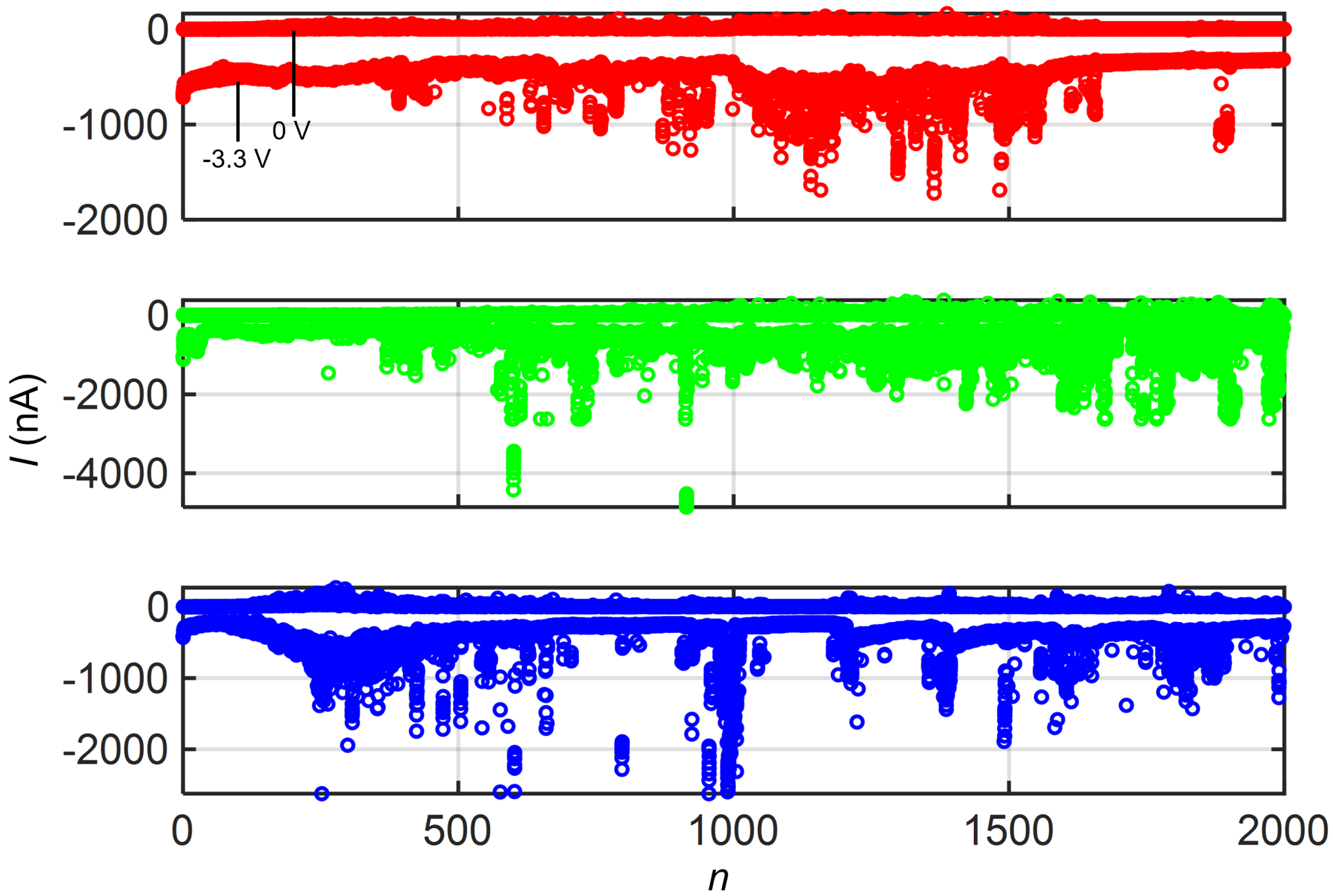

Figure S30: **Electrolysis current as a function of electrolysis cycles at** −3.3 V **for** 1 s **and** 0 V **for** 1 s**.** The cyclic protocol consisted of 1 s at −3.3 V followed by 1 s at 0 V and was repeated for 2000 cycles for each electrode.

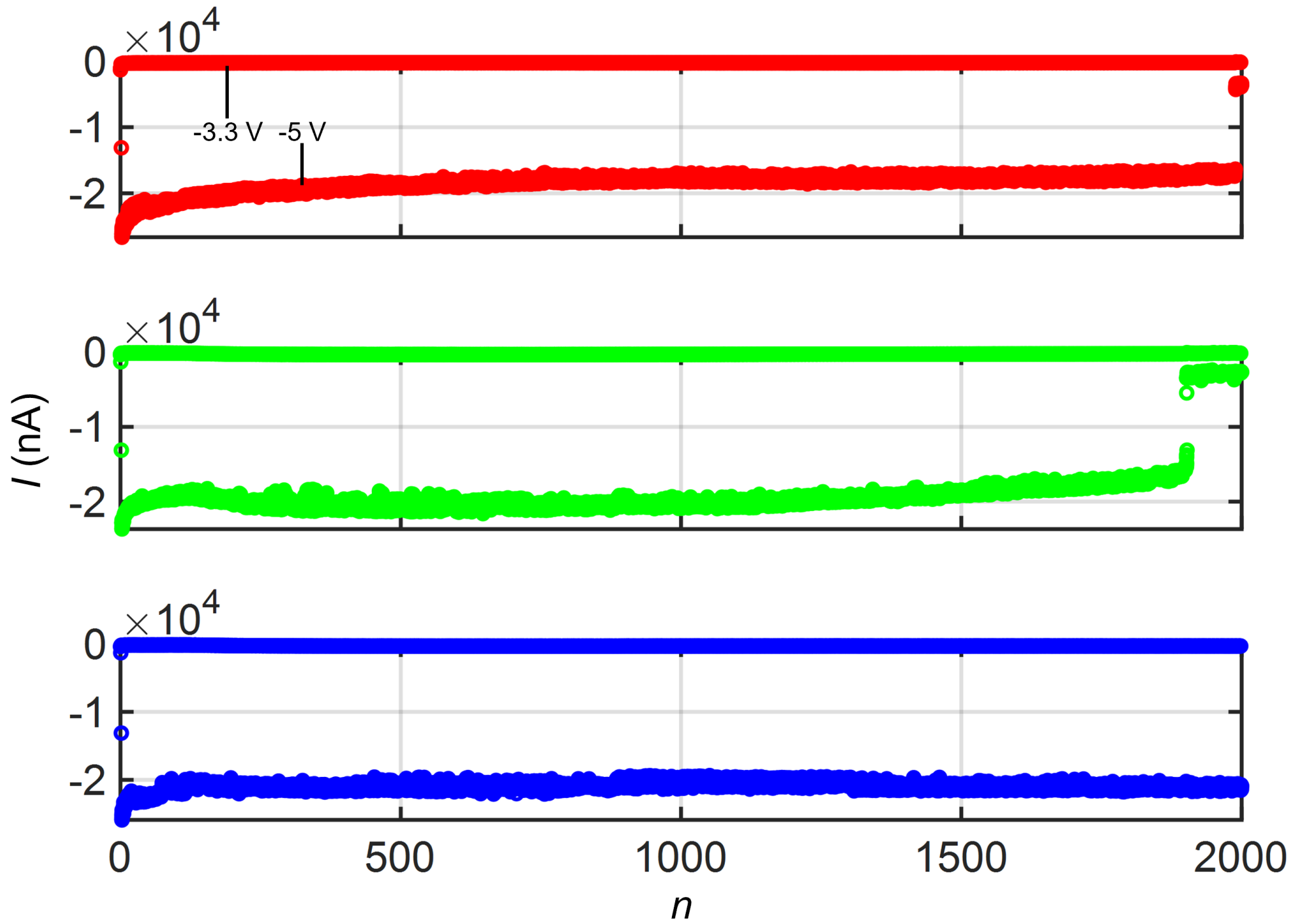

Figure S31: **Electrolysis current as a function of electrolysis cycles at** −3.3 V **for** 1 s **and** −5 V **for** 1 s**.** The cyclic protocol consisted of 1 s at −3.3 V followed by 1 s at −5 V and was repeated for 2000 cycles for each electrode.

Caption for Movie S1. Bubble state switching on an electrode, including bubble generating, bubble oscillating, bubble floating, and bubble shooting. Electrolysis voltage for bubble generation was −3.3 V. Electrolysis voltage for bubbling was −5 V. Ultrasound frequency and voltage for bubble oscillation were 74 kHz and 10 V_{PP}. Ultrasound frequency and voltage for bubble shooting were 74 kHz and 20 V_{PP}. The video was played in real time.

Caption for Movie S2. Demonstrations of programmable flow topologies corresponding to different microbubble arrangements with a 6 × 6 microelectrode array. Electrolysis voltage for bubble generation was −3.3 V. Electrolysis voltage for bubbling was −5 V. Ultrasound frequency and voltage for bubble oscillation were 48 kHz and 8 V_{PP}. Ultrasound frequency and voltage for bubble shooting upward were 57 kHz and 20 V_{PP}. Ultrasound frequency and voltage for bubble shooting downward were 262 kHz (an additional high-frequency shooting example) and 20 V_{PP}. The video was played in real time.

Caption for Movie S3. Stepwise size modulation of a single microbubble led to intensified volume oscillations and enhanced radial acoustic streaming. Electrolysis voltage for bubble generation was −3.3 V. Ultrasound frequency and voltage for bubble oscillation were 62 kHz and 10 V_{PP}. The video was played in real time.

Caption for Movie S4. Controlled bubble interaction. Left panel: Direction shifting of polarized flow. Middle panel: Dynamic double-bubble system. Right panel: Extended triple-bubble system. Electrolysis voltage for bubble generation was −3.3 V. Ultrasound frequency and voltage for bubble oscillation were 90 kHz and 7 V_{PP}. The video was played in real time.

Caption for Movie S5. Active bubble oscillation decoupling. Left panel: Active oscillation decoupling by changing the size of the bottom bubble in real time. Electrolysis voltage for bubble generation was −3.3 V. Ultrasound frequency and voltage for bubble oscillation were 45 kHz and 10 V_{PP}. Right panel: Active oscillation decoupling by controlling the number of bubbles over time. Electrolysis voltage for bubble generation was −3.3 V. Ultrasound frequency and voltage for bubble oscillation were 30 kHz, 45 kHz, 79 kHz, and 10 V_{PP}. The video was played in real time.

Caption for Movie S6. Dynamic acoustofluidic programming. Microbubbles with a similar size were generated and excited at different positions in sequence with the corresponding reconfigured flows. Electrolysis voltage for bubble generation was −3.3 V. Ultrasound frequency and voltage for bubble oscillation were 74 kHz and 10 V_{PP}. The video was played in real time.

Caption for Movie S7. Programmable flow topologies. Various flow topologies corresponding to different microbubble arrangements with a 3 × 3 microelectrode array. Electrolysis voltage for bubble generation was −3.3 V. Ultrasound voltage for bubble oscillation was 10 V_{PP}. The video was played in real time.

Caption for Movie S8. Bubble removal. Left panel: Globally erasure of all bubbles. Electrolysis voltage for bubble generation was −3.3 V. Ultrasound frequency and voltage were 57 kHz and 20 V_{PP}. Right panel: Selective removal of a single bubble. Electrolysis voltage for bubble generation was −3.3 V. Electrolysis voltage for bubbling was −5 V. Ultrasound frequency and voltage for bubble oscillation were 60 kHz and 5 V_{PP}. The video was played in real time.

Caption for Movie S9. Directional bubble shooting by switching the ultrasound excitation frequencies. The ultrasound frequencies were switched in the following order: 74 kHz, 90 kHz, 84 kHz, 88 kHz, 94 kHz, 81 kHz, and 74 kHz. Ultrasound voltage was 20 V_{PP}. Electrolysis voltage for bubbling was −5 V. The video was played in real time.

Caption for Movie S10. Bubble shooting recorded at different enlargement scales. Ultrasound frequency and voltage were 54 kHz and 20 V_{PP}**. For the left and middle panels, the electrolysis voltage for bubbling was** −5 V**. For the right panel, the electrolysis voltage was varied between** −4 V **and** −8 V**. The video was played in real time.**

Caption for Movie S11. Bubble shooting dynamics recorded by a high-speed camera. Electrolysis voltage for bubbling was −5 V. Ultrasound voltage was 20 V_{PP}. The video was recorded at 1,000 fps and was played at 0.1 × real time.

Caption for Movie S12. Selective and coordinated bubble shooting. Left panel: Selective bubble shooting. Ultrasound frequency and voltage for bubble shooting were 68 kHz and 10 V_{PP}. Right panel: Bubble shooting with a 3×3 microelectrode array. Ultrasound frequencies for bubble shooting toward the bottom right and bottom left were 64 kHz and 71 kHz, respectively. Ultrasound voltage was 20 V_{PP}. Electrolysis voltage for bubbling was -5 V. The video was played in real time.

Captions for files S1 to S3. All files are available on Zenodo at the following link: https://zenodo.org/records/22780888.

Caption for COMSOL Output Report S1. Simulations of acoustic streaming and bubble-oscillation phases. Related simulations were shown in **Fig. 3**, **Supplementary Fig. S8**, and **Supplementary Note S1**.

Caption for COMSOL Output Report S2. Simulations of substrate vibration, acoustic pressure, and thermal distribution. Related simulations were shown in **Supplementary Fig. S3** and **Fig. S13**.

Caption for COMSOL Output Report S3. Simulations of prescribed acoustic fields and acoustic radiation forces. Related simulations were shown in **Supplementary Note S3**.

REFERENCES

1. Z. Miao, T. Feng, A. Ren, H. Huang, S. Li, Y. Xue, Y. Shen, X. Liu, Z. Chen, P. Jin, T.-W. Wong, F. Zhou, T. Li, X. Yang, W. Yang, Acoustically powered micro-clampbot for single-particle transportation. *Sci. Adv.* **11**, eady3213 (2025).

2. M. Kaynak, A. Dolev, M. S. Sakar, 3D printed acoustically programmable soft microactuators. *Soft Robot.* **10**, 246–257 (2023).

3. T. Qiu, F. Adams, S. Palagi, K. Melde, A. G. Mark, U. Wetterauer, A. Miernik, P. Fischer, Wireless acoustic-surface actuators for miniaturized endoscopes. *ACS Appl. Mater. Interfaces* **9**, 42536–42543 (2017).

4. N. Mahkam, M. C. Ugurlu, S. K. Kalva, A. Aghakhani, D. Razansky, M. Sitti, Multiorifice acoustic microrobot for boundary-free multimodal 3D swimming. *Proc. Natl. Acad. Sci. U.S.A.* **122**, e2417111122 (2025).

5. C. Dillinger, A. Rasaiah, A. Vogel, C. Bahou, K. Monastyrskaya, A. H. Gheinani, D. Ahmed, Real-time color flow mapping of ultrasound microrobots. *Sci. Adv.* **11**, eadt8887 (2025).

6. L. Chai, D. Kim, S. K. Chung, K. Y. Lee, Magnetically maneuverable micromixer using acoustically excited bubbles. *J. Micromech. Microeng.* **35**, 035001 (2025).

7. H. Han, X. Ma, W. Deng, J. Zhang, S. Tang, O. S. Pak, L. Zhu, E. Criado-Hidalgo, C. Gong, E. Karshalev, J. Yoo, M. You, A. Liu, C. Wang, H. K. Shen, P. N. Patel, C. L. Hays, P. J. Gunnarson, L. Li, Y. Zhang, J. O. Dabiri, L. V. Wang, M. G. Shapiro, D. Wu, Q. Zhou, J. R. Greer, W. Gao, Imaging-guided bioresorbable acoustic hydrogel microrobots. *Sci. Robot.* **9**, eadp3593 (2024).

8. J. Janiak, Y. Li, Y. Ferry, A. A. Doinikov, D. Ahmed, Acoustic microbubble propulsion, train-like assembly and cargo transport. *Nat. Commun.* **14**, 4705 (2023).

9. L. Ren, N. Nama, J. M. McNeill, F. Soto, Z. Yan, W. Liu, W. Wang, J. Wang, T. E. Mallouk, 3D steerable, acoustically powered microswimmers for single-particle manipulation. *Sci. Adv.* **5**, eaax3084 (2019).

10. D. Ahmed, M. Lu, A. Nourhani, P. E. Lammert, Z. Stratton, H. S. Muddana, V. H. Crespi, T. J. Huang, Selectively manipulable acoustic-powered microswimmers. *Sci. Rep.* **5**, 9744 (2015).

11. T. Luo, M. Wu, Biologically inspired micro-robotic swimmers remotely controlled by ultra-sound waves. *Lab Chip* **21**, 4095–4103 (2021).

12. J. Jeong, D. Jang, D. Kim, D. Lee, S. K. Chung, Acoustic bubble-based drug manipulation: Carrying, releasing and penetrating for targeted drug delivery using an electromagnetically actuated microrobot. *Sens. Actuators A Phys.* **306**, 111973 (2020).

13. X. Liu, Y. Li, F. Liu, Q. Shi, L. Dong, Q. Huang, T. Arai, T. Fukuda, μSonic-hand: Biomedical micromanipulation driven by acoustic gas-liquid-solid interactions. *Sci. Adv.* **11**, eads8167 (2025).

14. Z. Zhang, L. K. Allegrini, N. Yanagisawa, Y. Deng, S. C. Neuhauss, D. Ahmed, Sonorotor: An acoustic rotational robotic platform for zebrafish embryos and larvae. *IEEE Robot. Autom. Lett.* **8**, 2598–2605 (2023).

15. D. Ahmed, A. Ozcelik, N. Bojanala, N. Nama, A. Upadhyay, Y. Chen, W. Hanna-Rose, T. J. Huang, Rotational manipulation of single cells and organisms using acoustic waves. *Nat. Commun.* **7**, 11085 (2016).

16. R. Goyal, A. G. Athanassiadis, Z. Ma, P. Fischer, Amplification of acoustic forces using microbubble arrays enables manipulation of centimeter-scale objects. *Phys. Rev. Lett.* **128**, 254502 (2022).

17. T. Qiu, S. Palagi, A. G. Mark, K. Melde, F. Adams, P. Fischer, Wireless actuation with functional acoustic surfaces. *Appl. Phys. Lett.* **109**, 191602 (2016).

18. A. Marzo, B. W. Drinkwater, Holographic acoustic tweezers. *Proc. Natl. Acad. Sci. U.S.A.* **116**, 84–89 (2019).

19. L. Wang, Y. Wu, Y. Tang, H. Wei, T. Lyu, X. Zhang, T.-Y. Huang, Reconfigurable acoustofluidic microvortices for selective microcargo delivery. *Adv. Sci.* **13**, e21612 (2026).

20. M. Medany, L. Piglia, L. Achenbach, S. K. Mukkavilli, D. Ahmed, Model-based reinforcement learning for ultrasound-driven autonomous microrobots. *Nat. Mach. Intell.* **7**, 1076–1090 (2025).

21. L. Meng, X. Liu, Y. Wang, W. Zhang, W. Zhou, F. Cai, F. Li, J. Wu, L. Xu, L. Niu, H. Zheng, Sonoporation of cells by a parallel stable cavitation microbubble array. *Adv. Sci.* **6**, 1900557 (2019).

22. C. B. Dayan, N. Mahkam, N. O. Dogan, V. Liimatainen, D. Hiz, A. Ilgezdi, D. Razansky, A. Aghakhani, M. Sitti, Multiscale acoustic bubble actuators using bioinspired liquid-repellent microstructures. *Adv. Mater. Technol.* **11**, e01532 (2026).

23. C. Bai, Z. Chen, Y. Li, Y. Chen, Q. Shi, Q. Huang, T. Fukuda, T. Arai, X. Liu, Acoustic rising microbubbles for efficient liquid operations. *Cyborg Bionic Syst.* **7**, 0449 (2026).

24. Z. Shi, Z. Zhang, J. Schnermann, S. C. Neuhauss, N. Nama, R. Wittkowski, D. Ahmed, Ultrasound-driven programmable artificial muscles. *Nature* **646**, 1096–1104 (2025).

25. Y. Gao, M. Wu, Y. Lin, W. Zhao, J. Xu, Acoustic bubble-based bidirectional micropump. *Microfluid. Nanofluid.* **24**, 29 (2020).

26. Q. Tang, F. Liang, L. Huang, P. Zhao, W. Wang, On-chip simultaneous rotation of large-scale cells by acoustically oscillating bubble array. *Biomed. Microdevices* **22**, 13 (2020).

27. X. Li, Z. Deng, W. Zhang, W. Zhou, X. Liu, H. Quan, J. Li, P. Li, Y. Li, C. Hu, F. Li, L. Niu, Z. Tian, L. Meng, H. Zheng, Oscillating microbubble array-based metamaterials (OMAMs) for rapid isolation of high-purity exosomes. *Sci. Adv.* **11**, eadu8915 (2025).

28. S. Mohanty, A. Paul, P. M. Matos, J. Zhang, J. Sikorski, S. Misra, CeFlowBot: A biomimetic flow-driven microrobot that navigates under magneto-acoustic fields. *Small* **18**, e2105829 (2022).

29. K. Nguyen, A. Swain, Y. Zheng, A. Vasa, A. Jain, P. Phaneesh, M. Nouri, A. Kuo, "Optothermally driven bubble etching for sustainable and on-demand nanomanufacturing," in *Optical Trapping and Optical Micromanipulation XXII* (SPIE, 2025), PC135800U.

30. H. Joh, B. Lian, S.-i. Hsueh, Z. Ma, K.-J. Lee, S.-Y. Zheng, P. Fischer, D. E. Fan, Massively parallel microbubble nano-assembly. *Nat. Commun.* **16**, 6743 (2025).

31. J. H. Shin, J. Seo, J. Hong, S. K. Chung, Hybrid optothermal and acoustic manipulations of microbubbles for precise and on-demand handling of micro-objects. *Sens. Actuators B Chem.* **246**, 415–420 (2017).

32. L. Lin, X. Peng, Z. Mao, W. Li, M. N. Yogeesh, B. B. Rajeeva, E. P. Perillo, A. K. Dunn, D. Akinwande, Y. Zheng, Bubble-pen lithography. *Nano Lett.* **16**, 701–708 (2016).

33. W. Gao, J. Wang, Synthetic micro/nanomotors in drug delivery. *Nanoscale* **6**, 10486–10494 (2014).

34. Q. Gao, F. Li, J. Gao, E. Zhang, Y. Su, N. Qin, H. Ye, J. Puigmartí, G. Cantarella, S. Schuerle, J. Wu, B. J. Nelson, S. Pané, Reconfigurable magnetic soft microrobot for acoustically triggered targeted bacterial sterilization. *Adv. Funct. Mater.* **36**, e18017 (2026).

35. D. Wang, Z. Liu, H. Zhao, H. Qin, G. Bai, C. Chen, P. Shi, Y. Du, Y. Zhao, W. Liu, D. Wang, G. Zhou, X. He, C. Dai, Launching by cavitation. *Science* **389**, 935–939 (2025).

36. M. Cattaneo, G. Guerriero, G. Shakya, L. A. Krattiger, L. G. Paganella, M. L. Narciso, O. Supponen, Cyclic jetting enables microbubble-mediated drug delivery. *Nat. Phys.* **21**, 590–598 (2025).

37. X. Huang, P.-B. Liu, G.-Y. Niu, H.-B. Hu, Experimental study on the translation behavior of an in-situ bubble pair in the ultrasonic field. *Ultrason. Sonochem.* **112**, 107188 (2025).

38. M. U. Jung, Y. C. Kim, G. Bournival, S. Ata, Tailoring bubble size through acoustic-assisted microbubble generation. *AIChE J.* **71**, e18802 (2025).

39. H. J. Cho, J. P. Mizerak, E. N. Wang, Turning bubbles on and off during boiling using charged surfactants. *Nat. Commun.* **6**, 8599 (2015).

40. C.-H. Choi, M. David, Z. Gao, A. Chang, M. Allen, H. Wang, C. H. Chang, Large-scale generation of patterned bubble arrays on printed bi-functional boiling surfaces. *Sci. Rep.* **6**, 23760 (2016).

41. Y. Lee, V. K. Bandari, J. S. McCaskill, P. Adluri, D. Karnaushenko, D. D. Karnaushenko, O. G. Schmidt, Si chiplet-controlled 3D modular microrobots with smart communication in natural aqueous environments. *Sci. Robot.* **10**, eadu6007 (2025).

42. G. Zhang, S. Yang, J. F. Yang, D. Gonzalez-Medrano, M. Z. Miskin, V. B. Koman, Y. Zeng, S. X. Li, M. Kuehne, A. T. Liu, A. M. Brooks, M. Kumar, M. S. Strano, High energy density picoliter-scale zinc-air microbatteries for colloidal robotics. *Sci. Robot.* **9**, eade4642 (2024).

43. G. Wu, I. Heck, N. Zhang, G. Phaup, X. Zhang, Y. Wu, D. E. Stalla, Z. Weng, H. Sun, H. Li, Z. Zhang, S. Ding, D.-P. Li, Y. Zhang, Wireless, battery-free push-pull microsystem for membrane-free neurochemical sampling in freely moving animals. *Sci. Adv.* **8**, eabn2277 (2022).

44. Z. Ma, K. Melde, A. G. Athanassiadis, M. Schau, H. Richter, T. Qiu, P. Fischer, Spatial ultrasound modulation by digitally controlling microbubble arrays. *Nat. Commun.* **11**, 4537 (2020).

45. K. H. Lee, J. H. Lee, J. M. Won, K. Rhee, S. K. Chung, Micromanipulation using cavitational microstreaming generated by acoustically oscillating twin bubbles. *Sens. Actuators A Phys.* **188**, 442–449 (2012).

46. S. K. Chung, Y. Zhao, S. K. Cho, On-chip creation and elimination of microbubbles for a micro-object manipulator. *J. Micromech. Microeng.* **18**, 095009 (2008).

47. A. Bashkatov, F. Bürkle, Ç. Demirkır, W. Ding, V. Sanjay, A. Babich, X. Yang, G. Mutschke, J. Czarske, D. Lohse, D. Krug, L. Büttner, K. Eckert, Electrolyte droplet spraying in H2 bubbles during water electrolysis under normal and microgravity conditions. *Nat. Commun.* **16**, 4580 (2025).

48. L. Deng, L. Jin, L. Yang, C. Feng, A. Tao, X. Jia, Z. Geng, C. Zhang, X. Cui, J. Shi, Bubble evolution dynamics in alkaline water electrolysis. *eScience* **5**, 100353 (2025).

49. H. Tüysüz, Alkaline water electrolysis for green hydrogen production. *Acc. Chem. Res.* **57**, 558–567 (2024).

50. K. Matis, E. Peleka, Alternative flotation techniques for wastewater treatment: Focus on electroflotation. *Sep. Sci. Technol.* **45**, 2465–2474 (2010).

51. S. Boinpally, A. Kolla, J. Kainthola, R. Kodali, J. Vemuri, A state-of-the-art review of the electrocoagulation technology for wastewater treatment. *Water Cycle* **4**, 26–36 (2023).

52. M. Bharti, P. P. Das, M. K. Purkait, A review on the treatment of water and wastewater by electrocoagulation process: Advances and emerging applications. *J. Environ. Chem. Eng.* **11**, 111558 (2023).

53. F. Sepahi, R. Verzicco, D. Lohse, D. Krug, Mass transport at gas-evolving electrodes. *J. Fluid Mech.* **983**, A19 (2024).

54. X. Yang, F. Karnbach, M. Uhlemann, S. Odenbach, K. Eckert, Dynamics of single hydrogen bubbles at a platinum microelectrode. *Langmuir* **31**, 8184–8193 (2015).

55. M. Minnaert, XVI. On musical air-bubbles and the sounds of running water. *Philos. Mag.* **16**, 235–248 (1933).

56. M. Versluis, D. E. Goertz, P. Palanchon, I. L. Heitman, S. M. van der Meer, B. Dollet, N. de Jong, D. Lohse, Microbubble shape oscillations excited through ultrasonic parametric driving. *Phys. Rev. E* **82**, 026321 (2010).

57. A. A. Doinikov, S. Cleve, G. Regnault, C. Mauger, C. Inserra, Acoustic microstreaming produced by nonspherical oscillations of a gas bubble. I. Case of modes 0 and *m*. *Phys. Rev. E* **100**, 033104 (2019).

58. A. A. Doinikov, T. Combriat, P. A. Thibault, P. Marmottant, Acoustic streaming produced by a cylindrical bubble undergoing volume and translational oscillations in a microfluidic channel. *Phys. Rev. E* **94**, 033109 (2016).

59. J. Mondal, Y. Wu, A. Mishra, F. Akbaridoust, I. Marusic, P. Ghosh, M. Ashokkumar, Bubble oscillations at low frequency ultrasound for biological applications. *Ultrason. Sonochem.* **104**, 106816 (2024).

60. V. Poulichet, A. Huerre, V. Garbin, Shape oscillations of particle-coated bubbles and directional particle expulsion. *Soft Matter* **13**, 125–133 (2017).

61. G. Lajoinie, Y. Luan, E. Gelderblom, B. Dollet, F. Mastik, H. Dewitte, I. Lentacker, N. de Jong, M. Versluis, Non-spherical oscillations drive the ultrasound-mediated release from targeted microbubbles. *Commun. Phys.* **1**, 22 (2018).

62. N. A. Pelekasis, A. Gaki, A. Doinikov, J. A. Tsamopoulos, Secondary Bjerknes forces between two bubbles and the phenomenon of acoustic streamers. *J. Fluid Mech.* **500**, 313–347 (2004).

63. A.-M. Zhang, S.-M. Li, P. Cui, S. Li, Y.-L. Liu, A unified theory for bubble dynamics. *Phys. Fluids* **35**, 033323 (2023).

64. Y. Shen, L. Zhang, Y. Wu, W. Chen, The role of the bubble-bubble interaction on radial pulsations of bubbles. *Ultrason. Sonochem.* **73**, 105535 (2021).

65. F. Mekki-Berrada, T. Combriat, P. Thibault, P. Marmottant, Interactions enhance the acoustic streaming around flattened microfluidic bubbles. *J. Fluid Mech.* **797**, 851–873 (2016).

66. J. Jiao, Y. He, S. E. Kentish, M. Ashokkumar, R. Manasseh, J. Lee, Experimental and theoretical analysis of secondary Bjerknes forces between two bubbles in a standing wave. *Ultrasonics* **58**, 35–42 (2015).

67. T. G. Leighton, A. J. Walton, M. J. W. Pickworth, Primary Bjerknes forces. *Eur. J. Phys.* **11**, 47–50 (1990).

68. S. Park, L. Liu, Ç. Demirkır, O. van der Heijden, D. Lohse, D. Krug, M. T. M. Koper, Solutal Marangoni effect determines bubble dynamics during electrocatalytic hydrogen evolution. *Nat. Chem.* **15**, 1532–1540 (2023).

69. S. Lee, S. Ghajari, S. Sadeghi, Y. Zheng, H. Zahr, A. J. Cortese, W. Gu, K. Choe, A. Mok, M. Wallace, R. Jiao, C. Wu, J. C. Werth, W. Fan, P. Mogalipuvvu, J. U. Park, S. Zhao, C. Smart, T. A. Cleland, M. R. Warden, J. Lammerding, T. Wang, J. H. Goldberg, P. L. McEuen, C. Xu, A. C. Molnar, A subnanolitre tetherless optoelectronic microsystem for chronic neural recording in awake mice. *Nat. Electron.* **8**, 1259–1271 (2025).

70. M. Taghavi, W. Wang, K. Shim, J. Zhang, I. Cohen, A. Apsel, Coordinated behavior of autonomous microscopic machines through local electronic pulse coupling. *Sci. Robot.* **9**, eadn8067 (2024).

71. Y. Deng, A. Paskert, Z. Zhang, R. Wittkowski, D. Ahmed, An acoustically controlled helical microrobot. *Sci. Adv.* **9**, eadh5260 (2023).

72. Z. Liang, M. X. Lim, Q.-Z. Zhu, F. Mottes, J. Z. Kim, L. Guttieres, C. Smart, T. Pearson, C. X. Du, M. Brenner, P. McEuen, I. Cohen, Magnetic decoupling as a proofreading strategy for high-yield, time-efficient microscale self-assembly. *Proc. Natl. Acad. Sci. U.S.A.* **122**, e2502361122 (2025).

73. Q. Liu, W. Wang, H. Sinhmar, I. Griniasty, J. Z. Kim, J. T. Pelster, P. Chaudhari, M. F. Reynolds, M. C. Cao, D. A. Muller, A. B. Apsel, N. L. Abbott, H. Kress-Gazit, P. L. McEuen, I. Cohen, Electronically configurable microscopic metasheet robots. *Nat. Mater.* **24**, 109–115 (2025).

74. W. S. Harley, K. Kolesnik, M. Xu, D. E. Heath, D. J. Collins, 3D acoustofluidics via sub-wavelength micro-resonators. *Adv. Funct. Mater.* **33**, 2211422 (2023).

75. C. Chen, Y. Gu, J. Rufo, J. Zhang, K. Yang, Y. Chen, L. P. Lee, T. J. Huang, Acoustofluidic spin control for 3D particle manipulation in droplets. *Sci. Adv.* **11**, eadx0269 (2025).

76. A. A. Doinikov, Acoustic radiation force on a bubble: Viscous and thermal effects. *J. Acoust. Soc. Am.* **103**, 143–147 (1998).

77. C. P. Lee, T. G. Wang, Acoustic radiation force on a bubble. *J. Acoust. Soc. Am.* **93**, 1637–1640 (1993).

78. O. Louisnard, A simple model of ultrasound propagation in a cavitating liquid. Part II: Primary Bjerknes force and bubble structures. *Ultrason. Sonochem.* **19**, 66–76 (2012).

79. L. A. Crum, Bjerknes forces on bubbles in a stationary sound field. *J. Acoust. Soc. Am.* **57**, 1363–1370 (1975).

80. H. Chen, Z. Chen, Y. Li, Modulation of the secondary Bjerknes force in multi-bubble systems. *Ultrason. Sonochem.* **61**, 104814 (2020).

81. T. Barbat, N. Ashgriz, C.-S. Liu, Dynamics of two interacting bubbles in an acoustic field. *J. Fluid Mech.* **389**, 137–168 (1999).

82. P. Tho, R. Manasseh, A. Ooi, Cavitation microstreaming patterns in single and multiple bubble systems. *J. Fluid Mech.* ***576***, 191–233 (2007).

83. M. S. Plesset, A. Prosperetti, Bubble dynamics and cavitation. *Annu. Rev. Fluid Mech.* **9**, 145–185 (1977).

84. A. Prosperetti, Bubble phenomena in sound fields: Part one. *Ultrasonics* **22**, 69–77 (1984).

85. A. A. Doinikov, S. T. Zavtrak, On the mutual interaction of two gas bubbles in a sound field. *Phys. Fluids* **7**, 1923–1930 (1995).

86. M. Ida, Phase properties and interaction force of acoustically interacting bubbles: A complementary study of the transition frequency. *Phys. Fluids* **17**, 097107 (2005).

87. C. Wang, J. Cheng, Cavitation microstreaming generated by a bubble pair in an ultrasound field. *J. Acoust. Soc. Am.* **134**, 1675–1682 (2013).

88. A. A. Doinikov, A. Bouakaz, Microstreaming generated by two acoustically induced gas bubbles. *J. Fluid Mech.* **796**, 318–339 (2016).

89. S. M. van der Meer, B. Dollet, M. M. Voormolen, C. T. Chin, A. Bouakaz, N. de Jong, M. Versluis, D. Lohse, Microbubble spectroscopy of ultrasound contrast agents. *J. Acoust. Soc. Am.* **121**, 648–656 (2007).

90. A. A. Doinikov, G. Regnault, C. Mauger, P. Blanc-Benon, C. Inserra, Acoustic microstreaming produced by two interacting gas bubbles undergoing axisymmetric shape oscillations. *J. Fluid Mech.* **931**, A19 (2022).

91. Y. Ma, G. Zhang, T. Ma, Interaction of two bubbles with distortion in an acoustic field. *Ultrason. Sonochem.* **84**, 105953 (2022).

92. M. Settnes, H. Bruus, Forces acting on a small particle in an acoustical field in a viscous fluid. *Phys. Rev. E* **85**, 016327 (2012).

93. A. A. Doinikov, Acoustic radiation force on a spherical particle in a viscous heat-conducting fluid. I. General formula. *J. Acoust. Soc. Am.* **101**, 713–721 (1997).

94. K. Yosioka, Y. Kawasima, Acoustic radiation pressure on a compressible sphere. *Acustica* **5**, 167–173 (1955).

95. P. Marmottant, M. Versluis, N. de Jong, S. Hilgenfeldt, D. Lohse, High-speed imaging of an ultrasound-driven bubble in contact with a wall: “Narcissus” effect and resolved acoustic streaming. *Exp. Fluids* **41**, 147–153 (2006).

96. A. A. Doinikov, S. Zhao, P. A. Dayton, Modeling of the acoustic response from contrast agent microbubbles near a rigid wall. *Ultrasonics* **49**, 195–201 (2009).

97. A. O. Maksimov, Radiation force on a bubble located near an interface. *J. Acoust. Soc. Am.* **151**, 1464–1475 (2022).

98. S. A. Suslov, A. Ooi, R. Manasseh, Nonlinear dynamic behavior of microscopic bubbles near a rigid wall. *Phys. Rev. E* **85**, 066309 (2012).
